# Shifting spaces: Which disparity or dissimilarity measurement best summarize occupancy in multidimensional spaces?

**DOI:** 10.1002/ece3.6452

**Published:** 2020-07-05

**Authors:** Thomas Guillerme, Mark N. Puttick, Ariel E. Marcy, Vera Weisbecker

**Affiliations:** ^1^ School of Biological Sciences University of Queensland St. Lucia QLD Australia; ^2^ Department of Animal and Plant Sciences The University of Sheffield Sheffield UK; ^3^ Milner Centre for Evolution University of Bath Bath UK; ^4^ College of Science and Engineering Flinders University Adelaide SA Australia

**Keywords:** disparity, dissimilarity, ecology, evolution, multidimensionality, statistics

## Abstract

Multidimensional analysis of traits are now common in ecology and evolution and are based on trait spaces in which each dimension summarizes the observed trait combination (a morphospace or an ecospace). Observations of interest will typically occupy a subset of this space, and researchers will calculate one or more measures to quantify how organisms inhabit that space. In macroevolution and ecology, these measures called disparity or dissimilarity metrics are generalized as space occupancy measures. Researchers use these measures to investigate how space occupancy changes through time, in relation to other groups of organisms, or in response to global environmental changes. However, the mathematical and biological meaning of most space occupancy measures is vague with the majority of widely used measures lacking formal description. Here, we propose a broad classification of space occupancy measures into three categories that capture changes in size, density, or position. We study the behavior of 25 measures to changes in trait space size, density, and position on simulated and empirical datasets. We find that no measure describes all of trait space aspects but that some are better at capturing certain aspects. Our results confirm the three broad categories (size, density, and position) and allow us to relate changes in any of these categories to biological phenomena. Because the choice of space occupancy measures is specific to the data and question, we introduced https://tguillerme.shinyapps.io/moms/moms, a tool to both visualize and capture changes in space occupancy for any measurement. https://tguillerme.shinyapps.io/moms/moms is designed to help workers choose the right space occupancy measures, given the properties of their trait space and their biological question. By providing guidelines and common vocabulary for space occupancy analysis, we hope to help bridging the gap in multidimensional research between ecology and evolution.

## Graphical abstract

Different measurements of multidimensional space occupancy can give different results and are affected by the multidimensional space properties and biological question. This paper provides a guideline of what different measurements are capturing and in which context they can be useful for answering biological questions.

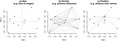

## 1 INTRODUCTION

Groups of species and environments share specific, recognizable, correlated characteristics: guilds or biomes with shared phenotypic, physiological, phylogenetic, or behavioral traits. Organisms or environments should therefore be studied as a set of traits rather than some specific traits in isolation (Donohue et al., 2013; Hopkins & Gerber, 2017). Biologists increasingly been using ordination techniques (see Legendre & Legendre, 2012 for a summary) to create multidimensional trait spaces to either explore properties of data or test hypotheses (e.g., Blonder, 2018; Bonhomme, Picq, Gaucherel, & Claude, 2014; Guillerme, 2018; Oksanen et al., 2007). For example, in palaeobiology, Wright (2017) used trait spaces to study how groups of species' characteristics change through time; in ecology, Jones et al. (2015) studied evidence of competition by looking at trait overlap between two populations. While different fields use a different set of terms for such approaches (Table 1), they actually focus on the same mathematical objects: matrices with columns representing an original or transformed trait value and rows representing observations (taxon, field site, etc.; Guillerme, 2018).

**Table 1:** Different terms are used for equivalent measures in mathematics, ecology and macroevolution

Mathematics	Ecology	Macroevolution	This paper
Matrix (*n* × *d*) with a structural relation between rows and columns	Functional space, morphospace, etc.	Morphospace, traitspace, etc.	Trait space
Rows (*n*)	Taxa, field sites, environments, etc.	Taxa, specimen, populations, etc.	Observations
Columns (*d*)	Traits, Ordination scores, distances, etc.	Traits, ordination scores, distances, etc.	Dimensions
Matrix subset (*m* × *d*; *m* ≤ *n*)	Treatments, phylogenetic group (clade), etc.	Clades, geological stratum, etc.	Group
Statistic (i.e., a measure)	Dissimilarity index or metric, hypervolume, functional diversity, etc.	Disparity metric or index	Space occupancy measure
Multidimensional analysis	Dissimilarity analysis, trait analysis, etc.	Disparity analysis, disparity‐through‐time, etc.	Multidimensional analysis

Ecologists and evolutionary biologists often use trait spaces with respect to the same fundamental questions: are groups occupying the same amount of trait space? Do some groups contain more species than others in the same amount of trait space? Are some specific factors correlated with different patterns of trait space occupancy? Because of the multidimensional nature of these trait spaces, it is often not possible to study them using bi‐ or tri‐variate techniques (Díaz et al., 2016; Hopkins & Gerber, 2017; Mammola, 2019). Studying the occupancy of trait spaces is done using disparity indices in macroevolution (Guillerme, 2018; Hopkins & Gerber, 2017; Wills, 2001) or comparing hypervolumes in ecology (Blonder, 2018; Díaz et al., 2016; Donohue et al., 2013; Mammola, 2019). Despite the commonalities between the measures used in ecology and evolution (which are often metric but do not necessarily need to be), surprisingly, little work has been published on their behavior (but see Ciampaglio, Kemp, & McShea, 2001; Mammola, 2019; Villéger, Mason, & Mouillot, 2008).

Different occupancy measures capture different aspects of trait space (Ciampaglio et al., 2001; Mammola, 2019; Villéger et al., 2008). This may be widely known, but to our knowledge it is infrequently mentioned in peer‐reviewed papers. First, space occupancy measures are often named as the biological aspect they are describing (“disparity” and “functional diversity”) rather than what they are measuring (e.g., the product of ranges), which obscures the differences and similarities between studies. Second, in many studies in ecology and evolution, authors have focused on measuring the size of the trait space (e.g., ellipsoid volume Donohue et al., 2013; hypervolume Díaz et al., 2016; Procrustes variance Marcy, Hadly, Sherratt, Garland, & Weisbecker, 2016; product of variance Wright, 2017). However, the size of the trait space only represents one aspects of occupancy, disregarding other measures such as the density (Harmon, Weir, Brock, Glor, & Challenger, 2008) or position (Ciampaglio et al., 2001; Wills, 2001). For example, if two groups have the same size, this can support certain biological conclusions. Yet, an alternative aspect of space occupancy may indicate that the groups' position are different, leading to a different biological conclusion (e.g., the groups are equally diverse but occupy different niches). Using measures that only capture one aspect of the trait space may restrain the potential of multidimensional analysis (Villéger et al., 2008).

Here, we propose a broad classification of space occupancy measures as used across ecology and evolution and study their power to detect changes in trait space occupancy in simulated and empirical data. Note, this does not account whether or not it is possible for a space to be occupied (e.g., some spaces may represent biologically impossible shapes); this, however, may be important in some cases, such as testing whether a region is infinite or not. We provide an assessment of each broad type of space occupancy measures along with a unified terminology to foster communication between ecology and evolution. Unsurprisingly, we found no one measure describes all changes in space and that the results from each measures are dependent on the characteristics of the space and the hypotheses.

There can be an infinite number of measures and that it is thus impossible to propose a comprehensive analysis for all the measures properties respective to how they measure changes in trait space. We therefore propose https://tguillerme.shinyapps.io/moms/moms, a tool for researchers to design, experiment and visualize their own space occupancy measure tailored for their project. The tool will help researchers understand the “null” behavior of the measures of interest.

### 1.1 Space occupancy measures

**Figure 1:**
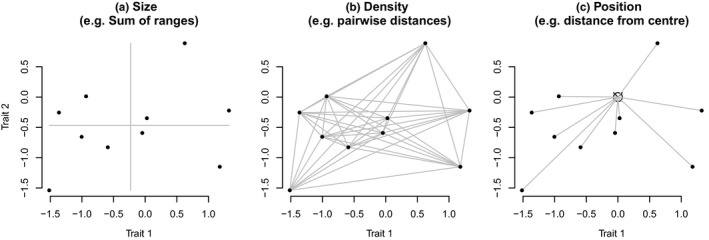
Different type of information captured by space occupancy measures: (a) size, (b) density, and (c) position

In this paper, we define trait spaces as any matrix where rows are observations and columns are traits, where both observations and traits are structurally related (e.g., there is a phylogenetic relation between observations—and traits, etc.). These traits can widely vary in number and types: they could be coded as discrete (e.g., presence or absence of a bone; Beck & Lee, 2014; Wright, 2017), continuous measurements (e.g., leaf area; Díaz et al., 2016) or more sophisticated measures (Fourier ellipses; Bonhomme et al., 2014; e.g., landmark position; Marcy et al., 2016). Traits can also be measured by using relative observations (e.g., community compositions; Jones et al., 2015) or distance between observations (e.g., Close, Friedman, Lloyd, & Benson, 2015). However, regardless of the methodology used to build a trait space, three broad occupancy measures can be used: the *size* which approximates the amount of space occupied, the *density* which approximates the distribution in space and the *position* which approximates the location in space (Figure 1; Villéger et al., 2008). Of course any combination of these three aspects is always possible.

#### 1.1.1 Size

Size captures the spread of a group in the trait space. They can be interpreted as the amount of the trait space that is occupied by observations. Typically, larger values for such measures indicate the presence of more extreme trait combinations. For example, if group A is bigger than B, the observations in A achieve more extreme trait combinations than in B. This type of measure is widely used in both ecology (e.g., the hypervolume; Blonder, 2018) and in evolution (e.g., the sum or product of ranges or variances; Wills, 2001).

Although size measures are suitable indicators of a group's trait space occupancy, they are limited to comparing the range of trait combinations between groups. Size measures do not take into account the distribution of the observations within a group and can often be insensitive to unoccupied “holes” in the trait space (overstimating the size; Blonder, 2018). They can make it difficult to determine whether all the observations are on the edge of the group's distribution or whether the size is simply driven by outliers.

#### 1.1.2 Density

Density gives an indication of the quantity of observations in the trait space. They can be interpreted as the distribution of the observations *within* a group in the trait space. Groups with higher density contain more observations (i.e., more observations per approximation of size) that will tend to be more similar to each other. For example, if group A is greater is size than group B and both have the same density (observations are equally distant within each group), similar mechanisms could be driving both groups' trait space occupancy. Indeed, this pattern could suggest that A is older and has had more time to achieve more extreme trait combinations under essentially the same process as younger, smaller group B (Endler, Westcott, Madden, & Robson, 2005). Note that density based measures can be sensitive to sampling. Density measures are less common compared to size measures, but they are still used in both ecology (e.g., the minimum spanning tree length; Oksanen et al., 2007) and evolution (e.g., the average pairwise distance; Harmon et al., 2008).

#### 1.1.3 Position

Position captures where a group lies in trait space. They can be interpreted as where a group lies in the trait space either relative to the space itself or relative to another group. For example, if group A has a different position than group B, A will have a different trait combination than in B. Position measures may be harder to interpret in multidimensional spaces. In a 2D space, two groups can be equally distant from a fixed point but in different parts of the space (left, right, up, or down—with the amount of parts of space increasing with dimensions). However, when thinking about unidimensional data, this measure is obvious: two groups A or B could have the same variance (size) with the same number of observations (density) but could have two different means and thus be in different positions. These measures are used in ecology to compare the position of two groups relative to each other (Mammola, 2019).

Note that, this classification into size, density, and position bears some similarities with Tucker et al. (2017) classifying phylogenetic diversity measurements into richness, divergence, and regularity categories. However, while Tucker et al. (2017) based their classification on the mathematical operation inherent to each metrics (the sum for richness, the mean for divergence, and the variance for regularity), our three broad classifications are based on their geometric properties regardless of the formula of each metric (e.g., the size of a space can be calculated using a sum, mean, or/and variance).

### 1.2 No measure to rule them all: benefits of considering multiple measures

The use of multiple measurements to assess trait space occupancy provides a more detailed characterization of occupancy changes. If the question is to look at how space occupancy changes in response to mass extinction, using a single space occupancy measure can miss part of the picture: a change in size could be decoupled from a change in position or density in trait space. For example, the Cretaceous‐Paleogene extinction (66 million years ago) shows an increase in size of the mammalian trait space (adaptive radiation; Halliday & Goswami, 2016) but more specific questions can be answered by looking at other aspects of trait space occupancy: does the radiation expand on previously existing morphologies (elaboration, increase in density; Endler et al., 2005) or does it explore new regions of the trait space (innovation, change in position; Endler et al., 2005)? Similarly, in ecology, if two groups have the same trait space size, the differences in density within these two groups is potentially illuminating: different selection pressure can lead to different density within equally sized groups. This can also be extended to more complex ecological concepts such as niche modelling (Qiao, Soberón, & Peterson, 2015).

Here, we provide the first interdisciplinary review of 25 space occupancy measures that uses the broad classification of measures into size, density, and position to capture pattern changes in trait space. We assess the behavior of measures using simulations and six interdisciplinary empirical datasets covering a wide range of potential data types and biological questions. We also introduce a tool for measuring occupancy in multidimensional space (https://tguillerme.shinyapps.io/moms/moms), which is a user‐friendly, open‐source, graphical interface to allow the tailored testing of measurement behavior for any use case. https://tguillerme.shinyapps.io/moms/moms will allow workers to comprehensively assess the properties of their trait space and the measures associated with their specific biological question.

## 2 METHODS

We tested how 25 space occupancy measures relate to each other, are affected by modifications of traits space and affect group comparisons in empirical data:

1. We simulated 13 different spaces with different sets of parameters;
2. We transformed these spaces by removing 50% of the observations following four different scenarios corresponding to different empirical scenarios: randomly, by size (e.g., expansion or reduction of niches), by density (e.g., different degrees of competition within a guild), and by position (e.g., ecological niche shift).
3. We measured occupancy on the resulting transformed spaces using eight different space occupancy measures;
4. We applied the same space occupancy measures to six empirical datasets (covering a range of disciplines and a range of dataset properties).

Note that the paper contains the results for only eight measures which were selected as representative of common measures covering the size, density, and position trait space aspects. The results for an additional 17 measures are available in the Appendix S4.

### 2.1 Generating spaces

We generated trait spaces using the following combinations of size, distributions, variance and correlation (Table 2):

**Table 2:** Different simulated space distribution. *Name* of the simulated space; *dimensions* of the matrix (row*columns); *distribution*(*s*) of the data on each dimensions (for the “Random,” dimensions are randomly chosen between Normal, Uniform or Lognormal); *dimension variance*: distribution of the variance between dimensions (when equal, the dimensions have the same variance); *correlation* between dimensions

Space name	Size	distribution(s)	Dimensions variance	Correlation
3D uniform	200*3	Uniform (min = −0.5, max = 0.5)	Equal	None
15D uniform	200*15	Uniform	Equal	None
50D uniform	200*50	Uniform	Equal	None
150D uniform	200*150	Uniform	Equal	None
50D uniform correlated	200*50	Uniform	Equal	Random (between 0.1 and 0.9)
3D normal	200*3	Normal (mean = 0, *SD* = 1)	Equal	None
15D normal	200*15	Normal	Equal	None
50D normal	200*50	Normal	Equal	None
150D normal	200*150	Normal	Equal	None
50D normal correlated	200*50	Normal	Equal	Random (between 0.1 and 0.9)
50D with random distributions	200*50	Normal, Uniform, Lognormal (meanlog = 0, sdlog = 1)	Equal	None
50D PCA‐like	200*50	Normal	Multiplicative	None
50D PCO‐like	200*50	Normal	Additive	None

The differences in trait space sizes (200 elements for 3, 15, 50 or 150 dimensions) reflects the range found in literature (e.g., Hopkins & Gerber, 2017; Mammola, 2019). We used a range of distributions (uniform, normal or a random combination of uniform, normal, and lognormal) to test the effect of observation distributions on the measurements. We used different levels of variance for each dimensions in the spaces by making the variance on each dimension either equal (*σ*_D1_; *σ*_D2_; *σ*_Di_) or decreasing (*σ*_D1_ < *σ*_D2_ < *σ*_D_ *_i_*) with the decreasing factor being either multiplicative (using the cumulative product of the inverse of the number of dimensions: $\prod_{i}^{d}\left(1/d\right)$ ) or additive ( $\sum_{i}^{d}\left(1/d\right)$ ). Both reductions of variance are used to illustrate the properties of ordinations where the variance decreases per dimensions (and normal win Multidimensional Scaling – MDS, PCO or PCoA; e.g., Close et al., 2015; lognormal in principal components analysis – PCA; e.g., Marcy et al., 2016; Wright, 2017; Healy, Ezard, Jones, Salguero‐Gómez, & Buckley, 2019). Finally, we added a correlation parameter to illustrate the effect of colinearity between traits (especially in nonordinated trait spaces). We repeated the simulation of each trait space 20 times (resulting in 260 spaces).

### 2.2 Spatial occupancy measures

We, then, calculated eight different measures on the resulting transformed spaces, including a new one, the average displacement, which we expect to be influenced by changes in trait space position (Table 3).

**Table 3:** List of measures with *n* being the number of observations, *d* the total number of dimensions, *k* any specific row in the matrix, *Centroid* being their mean and *σ* ^2^ their variance. Γ is the Gamma distribution and *λ_i_* the eigenvalue of each dimension and *q_i_* and *p_i_* are any pairs of coordinates

Name	Definition	Captures	Source	Notes
Average Euclidean distance from centroid	$\frac{\sqrt{\sum_{i}^{n}{\left(k_{n}-{\text{Centroid}}_{k}\right)}^{2}}}{d}$	Size	Laliberté and Legendre (2010)	The functional dispersion (FDis – without abundance)
Sum of variances	$\sum_{i}^{d}\sigma^{2}k_{i}$	Size	Foote (1992)	Common measure used in palaeobiology (Ciampaglio et al., 2001; Wills, 2001)
Sum of ranges	$\sum_{i}^{d}\text{||max}\left(d_{i}\right)-\min\left(d_{i}\right)\left|\right|$	Size	Foote (1992)	More sensitive to outliers than the sum of variances
Ellipsoid volume	$\frac{\pi^{d/2}}{\Gamma \left(\frac{d}{2}+1\right)}\prod_{i}^{d}\left(\lambda_{i}^{0.5}\right)$	Size	Donohue et al. (2013)	Less sensitive to outliers than the convex hull hypervolume (Blonder, 2018; Díaz et al., 2016)
Minimum spanning tree average distance	$\frac{\sum \left(\text{branch}\;\text{length}\right)}{n-1}$	Density	Sedgewick (1990)	Similar to the unscaled functional evenness (Villéger et al., 2008)
Minimum spanning tree distances evenness	$\frac{\sum \min\left(\frac{\text{branch}\;\text{length}}{\sum \text{branch}\;\text{length}}\right)-\frac{1}{n-1}}{1-\frac{1}{n-1}}$	Density	Villéger et al. (2008)	The functional evenness without weighted abundance (FEve; Villéger et al., 2008)
Average nearest neighbor distance	$\sqrt{\sum_{i}^{n}\min{\left(q_{i}-p_{i}\right)}^{2}})\times \frac{1}{n}$	Density	Foote (1992)	The density of pairs of observations
Average displacement	$\frac{\sqrt{\sum_{i}^{n}{\left(k_{n}\right)}^{2}}}{\sqrt{\sum_{i}^{n}{\left(k_{n}-{\text{Centroid}}_{k}\right)}^{2}}}$	Position	This paper	The ratio between the observations' position from their centroid and the center of the trait space (coordinates: 0, 0, 0,…). A value of 1 indicates that the observations' centroid is the center of the trait space

We selected these eight space occupancy measures to illustrate how they capture different aspects of space occupancy (not as an expression of our preference). These measures are specific to Euclidean and isotropic trait spaces (which is not necessary for all measures). The Appendix S4 contains the same analysis as described below, performed on 17 measures. Furthermore, https://tguillerme.shinyapps.io/moms/moms allows exploration into the effect of many more measures as well as the customization of measures by combining them or using user‐designed functions.

### 2.3 Measure comparisons

We compared the space occupancy measures correlations across all simulations between each pair of measures to assess their captured signal (Laliberté & Legendre, 2010; Villéger et al., 2008). We used the measures on the full 13 trait spaces described above. We, then, scaled the results and measured the pairwise Pearson correlation to test whether measures were capturing a similar signals or not using the psych package (Revelle, 2018).

### 2.4 Changing space

**Figure 2:**
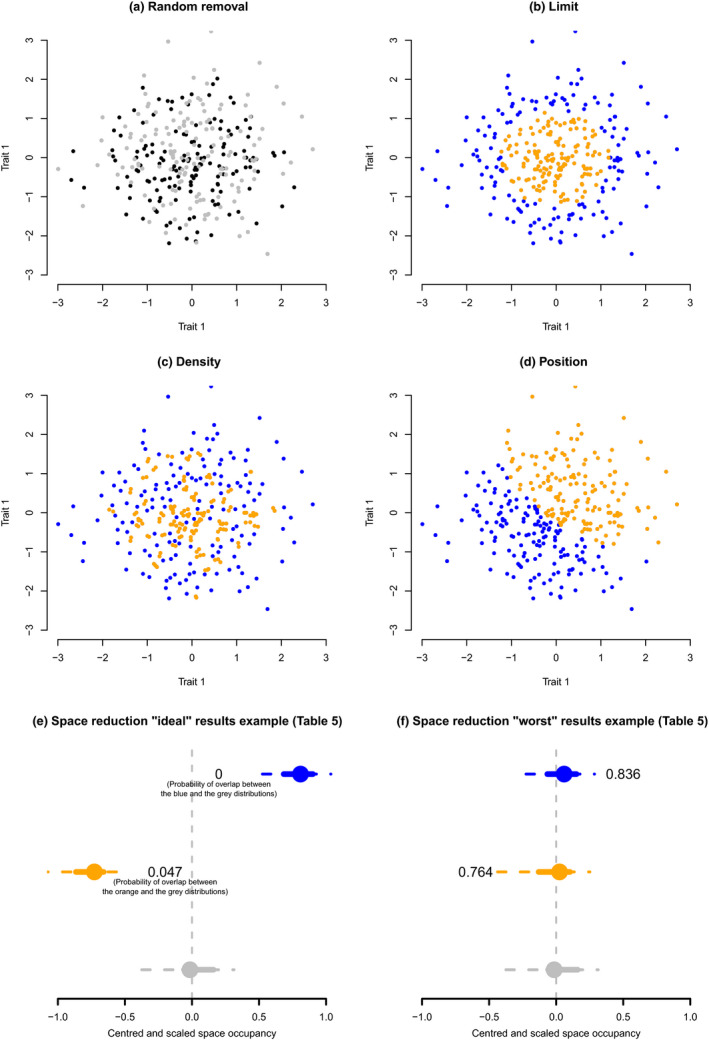
different type of space reduction. Each panel displays two groups of 50% of the data points each. Each group (orange and blue) are generated using the following algorithm: (a) randomly (the removed elements are displayed in black and the analyzed ones in gray); (b) by size (maximum and minimum limit); (c) by density (high and low); and (d) by position (positive and negative). Panel e et f represents two typical display of the reduction results displayed in Table 5: The dots represent the median space occupancy values across all simulations for each scenario of trait space change (Table 2), the solid and dashed line, respectively, the 50% and 95% confidence intervals. Results in gray are the random 50% reduction (panel a). Results in blue and orange represent the opposite scenarios from panels b, c, and d. The displayed value is the amount of overlap (Bhattacharrya Coefficient) between the blue or orange distributions and the gray one. Panel e and f shows respectively the “ideal” and “worst” results for any type of measures, where the space occupancy measurement respectively manages or fails to captures a specific type of reduction (i.e., size, position or density; Table 5)

To assess how the measures responded to changes within trait spaces, we removed 50% of observations each time using the following algorithms:

- *Randomly*: by randomly removing 50% of observations (Figure 2a). This reflects a “null” biological model of changes in trait space: the case when observations are removed regardless of their intrinsic characteristics. For example, if diversity is reduced by 50% but the space size remains the same, there is a decoupling between diversity and space occupancy (Ruta, Angielczyk, Fröbisch, & Benton, 2013). Our selected measures are expected to not be affected by this change.
- *Size*: by removing observations within a distance from the center of the trait space lower or greater than a radius ρ (where *ρ* is chosen such that 50% observations are selected) generating two limit removals: *maximum* and *minimum* (respectively, in orange and blue; Figure 2b). This can reflect a strict selection model where observations with trait values below or above a threshold are removed leading to an expansion or a contraction of the trait space. This type of change could be due to habitat destruction (e.g., Mammola et al., 2019) or to mass extinctions (e.g., Wright, 2017). Size measures are expected to be most affected by this change.
- *Density*: by removing any pairs of point with a distance *D* from each other where (where *D* is chosen such that 50% observations are selected) generating two density removals: *high* and *low* (respectively, in orange and blue; Figure 2c). This can reflect changes within groups in the trait space due to ecological factors (e.g., niche repulsion resulting in lower density; Grant & Grant, 2006). This type of change could be due to accelerated rates of evolution (Close et al., 2015) or to differences in modes of life in macroevolution (e.g., Healy et al., 2019). Density measures are expected to be most affected by this change.
- *Position*: by removing points similarly as for *Size* but using the distance from the furthest point from the center generating two position removals: *positive* and *negative* (respectively, in orange and blue; Figure 2d). This can reflect global changes in trait space (e.g., if an entire group remaining diverse but occupying a different niche). This type of change could be due changes in evolutionary trajectories (Endler et al., 2005) or to differences in ecosystem compositions (e.g., Jones et al., 2015). Position measures are expected to be most affected by this change.

The algorithm to select *ρ* or *D* is described in the Appendix S1.

Because occupancy measures are dependent on the space, we scaled and centered them between −1 and 1 to make them comparable (by subtracting the observed occupancy without reduction to all the measures of the reduced spaces and then divided it by the maximum observed occupancy). A value of 0 indicates no effect of the space reduction and >0 and <0, respectively, indicates an increase or decrease in the measure value. We, then, measured the amount of overlap between the nonrandom removals (size, density, and position) and the random removals using the Bhattacharrya Coefficient (Bhattacharyya, 1943).

#### 2.4.1 Measuring the effect of space and dimensionality

**Figure 3:**
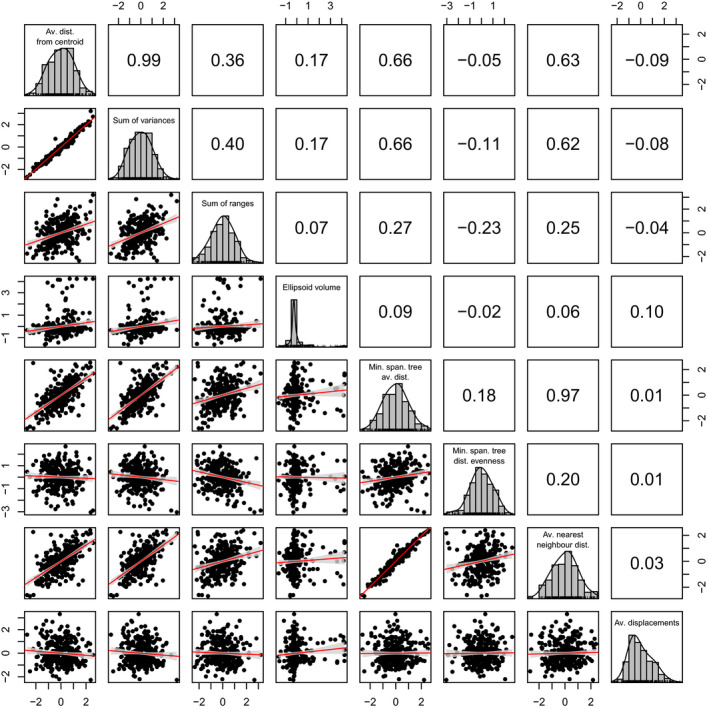
pairwise correlation between the scaled measures. Numbers on the upper right corner are the Pearson correlations. The red line are linear regressions (with the confidence intervals in gray). Av., average; dist., distance; min., minimum; span., spanning

Distribution differences and the number of dimensions can have an effect on the measure results. For example, in a normally distributed space, an increase in density can often lead to a decrease in size (though this is not necessarily true if the space is lognormal or uniform). High dimensional spaces (>10) are subject to the “curse of multidimensionality” (Bellman, 1957): Data become sparser with increasing number of dimensions. This can have two main consequences: (a) the probability of overlap between two groups decreases as a product of the number of dimensions; and (b) the amount of samples needed to “fill” the spaces increases exponentially see this interactive illustration by Toph Tucker. The “curse” can make the interpretation of high dimensional data counter‐intuitive. For example, if a group expands in multiple dimensions (i.e., increase in size), the actual hypervolume ( $\prod_{i}^{d}{\text{range}}_{Di}$ ) can decrease (Figure 3 and Tables 5 and 6).

We measured the effect of space distribution and dimensionality using an ANOVA (*occupancy*:*distribution* and *occupancy*:*dimensions*) by using all spaces with 50 dimensions and the uniform and normal spaces with equal variance and no correlation with 3, 15, 50, 100, and 150 dimensions (Table 2) for testing, respectively, the effect of distribution and dimensions. The results of the ANOVAs (*F* and *p*‐values) are reported in Table 5 (full results in Appendix S3).

### 2.5 Empirical examples

We analyzed the effect of the different space occupancy measures on six different empirical studies covering a range of fields that employ trait space analyses. For each of these studies, we generated trait spaces from the data published with the papers. We divided each trait spaces into two biologically relevant groups and tested whether the measures differentiated the groups in different ways. Both the grouping and the questions were based on a simplified version of the topics of these papers (with no intention to reanalyze the data and questions). The procedures to generate the data and the groups varies between studies and is detailed in the Appendix S2 (Table 4).

**Table 4:** Details of the six empirical trait spaces

Study	Field	Taxonomic group	Traits	Trait space	Size	Groups	Question
Beck and Lee (2014)	Palaeontology	Mammalia	Discrete morphological phylogenetic data	Ordination of a distance matrix (PCO)	106*105	52 crown vs. 54 stem	Are crown mammals more disparate than stem mammals?
Wright (2017)	Palaeontology	Crinoidea	discrete morphological phylogenetic data	Ordination of a distance matrix (PCO)	42*41	16 before vs. 23 after	Is there a difference in disparity before and after the Ordovician mass extinction?
Marcy et al. (2016)	Evolution	Rodentia	skull 2D landmark coordinates	Ordination of a Procrustes Superimposition (PCA)	454*134	225 *Megascapheus* vs. 229 *Thomomys*	Are two genera of gopher morphologically distinct?
Hopkins and Pearson (2016)	Evolution	Trilobita	3D landmark coordinates	Ordination of a Procrustes Superimposition (PCA)	46*46	36 adults vs. 10 juveniles	Are juvenile trilobites a subset of adult ones in trait space?
Jones et al. (2015)	Ecology	Plantae	Communities species compositions	Ordination of a Jaccard distance matrix (PCO)	48*47	24 aspens vs. 24 grasslands	Is there a difference in species composition between aspens and grasslands?
Healy et al. (2019)	Ecology	Animalia	Life history traits	Ordination of continuous traits (PCA)	285*6	83 ecthotherms vs. 202 endotherms	Do endotherms have more diversified life history strategies than ectotherms?

For each empirical trait space, we bootstrapped each group 500 times (Guillerme, 2018) and applied the eight space occupancy measure to each pairs of groups. We, then, compared the means of each groups using the Bhattacharrya Coefficient (Bhattacharyya, 1943).

## 3 RESULTS

### 3.1 Measure comparisons

Most measures of space were positively correlated (Pearson correlation of 0.99 for the average Euclidean distance from centroid and sum of variance or 0.97 for the average nearest neighbor distance and minimum spanning tree average length; Figure 3). The remaining measures were either somewhat correlated or had a negative pairwise distribution (ranging from 0.66 for the sum of variances and the ellipsoid volume to −0.09 between the average displacement and the average Euclidean distance from centroid; Figure 3). All measures but the ellipsoid volume were normally (or nearly normally) distributed (Figure 3).

### 3.2 Space shifting

As expected, some different measures capture different aspects of space occupancy. However, it can be hard to predict the behavior of each measure when 50% of the observations are removed. We observe a clear decrease in the median measure value in less than a third of the space reductions (10/36). In terms of change in size, only the average Euclidean distance from centroid and the sum of variances seem to capture a clear change in both directions. In terms of change in density, only the minimum spanning tree average distance and the average nearest neighbor distance seem to capture a clear change in both directions. And in terms of change in position, only the average displacement metric seems to capture a clear change in direction (albeit not in both directions). This is not surprising, since the notion of positions becomes more and more complex to appreciate as dimensionality increases (i.e., beyond left/right, up/down, and front/back).

### 3.3 Empirical example

As with the as for the simulations, there is no measure that summarizes all the aspects of distributions for empirical data. For all eight measures (except the ellipsoid volume) we see either one group or the other having a bigger mean than the other and no consistent case where a group has a bigger mean than the other for all the measures. For example, in the Beck and Lee (2014)'s dataset, there is a clear difference in size using the average Euclidean distance from centroid or the sum of variances (overlaps of, respectively, 0.175 and 0.159) but no overlap when measuring the size using the sum of ranges (0.966). However, for the Hopkins and Pearson (2016)'s dataset, this pattern is reversed (no clear differences for the average Euclidean distance from centroid or the sum of variances—0.701 and 0.865, respectively—but a clear difference for the sum of ranges (0). For each dataset, the absolute differences between each groups is not consistent depending on the measures. For example, in Hopkins and Pearson (2016)'s dataset, the orange group's mean is clearly higher than the blue one when measuring the sum of ranges (0) and the inverse is true when measuring the average displacement (0).

## 4 DISCUSSION

Here, we tested 25 measures of trait space occupancy on simulated and empirical datasets to assess how each measure captures changes in trait space size, density, and position. Our results show that the correlation between measures can vary both within and between measure categories (Figure 3), highlighting the importance of understanding the measure classification for the interpretation of results. Our simulations show that different measures capture different types of trait space change (Table 5), meaning that the use of multiple measures is important for comprehensive interpretation of trait space occupancy. We also show that the choice of measure impacts the interpretation of group differences in empirical datasets (Table 6).

**Table 5:**
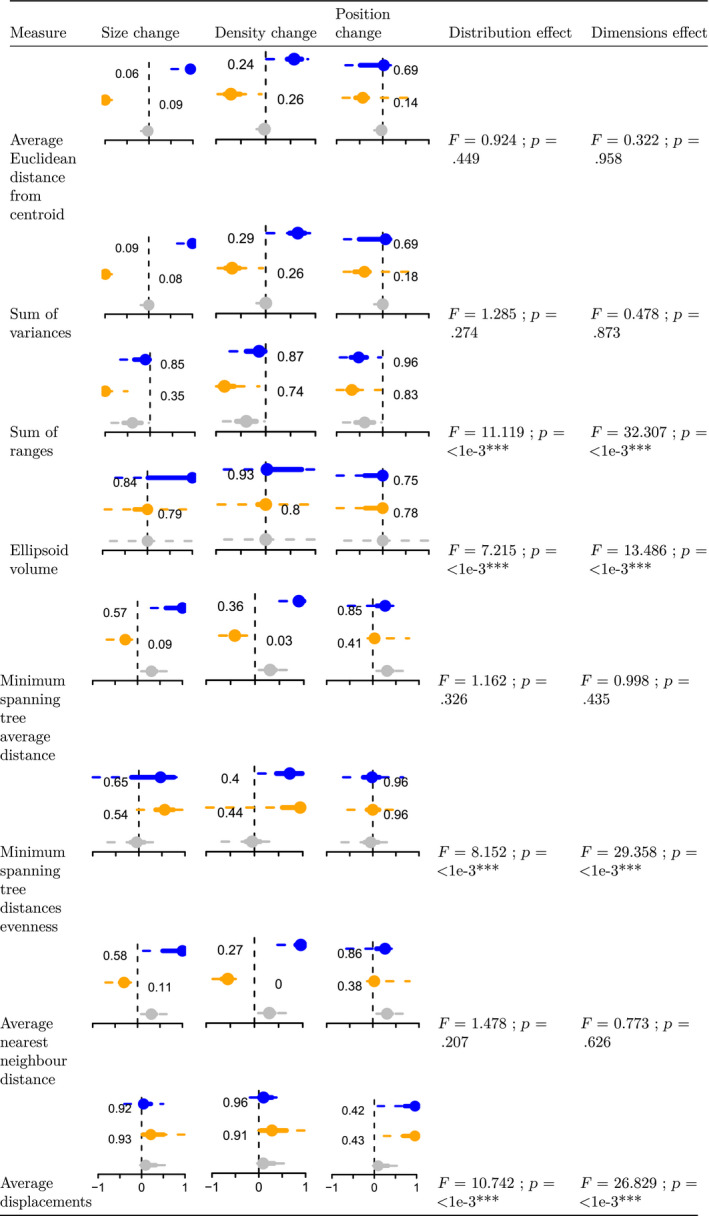
Results of the effect of space reduction, space dimension distributions and dimensions number of the different space occupancy measures. The dots represent the median space occupancy values across all simulations for each scenario of trait space change (Table 2), the solid and dashed line respectively the 50% and 95% confidence intervals. See Fig. 2 for details on the interpretation of the figures distributions and values. F‐values for distribution effect and dimensions effect represents respectively the effect of the ANOVAs space occupancy ~ distributions and space occupancy ~ dimension represent the ratio of sum squared difference within and between groups (the higher, the more the factor has an effect on the measure) and associated p‐values (0 ‘***’ 0.001 ‘**’ 0.01 ‘*’ 0.05 ‘.’ 0.1 ’’ 1). This figure illustrates how different measures can be influenced by different aspects of changes in the trait space. E.g. the Average Euclidean distance from centroid (row 1) captures mainly changes in size (column 1), but also captures changes in density (column 2) but does not capture changes in position (column 3)

**Table 6:**
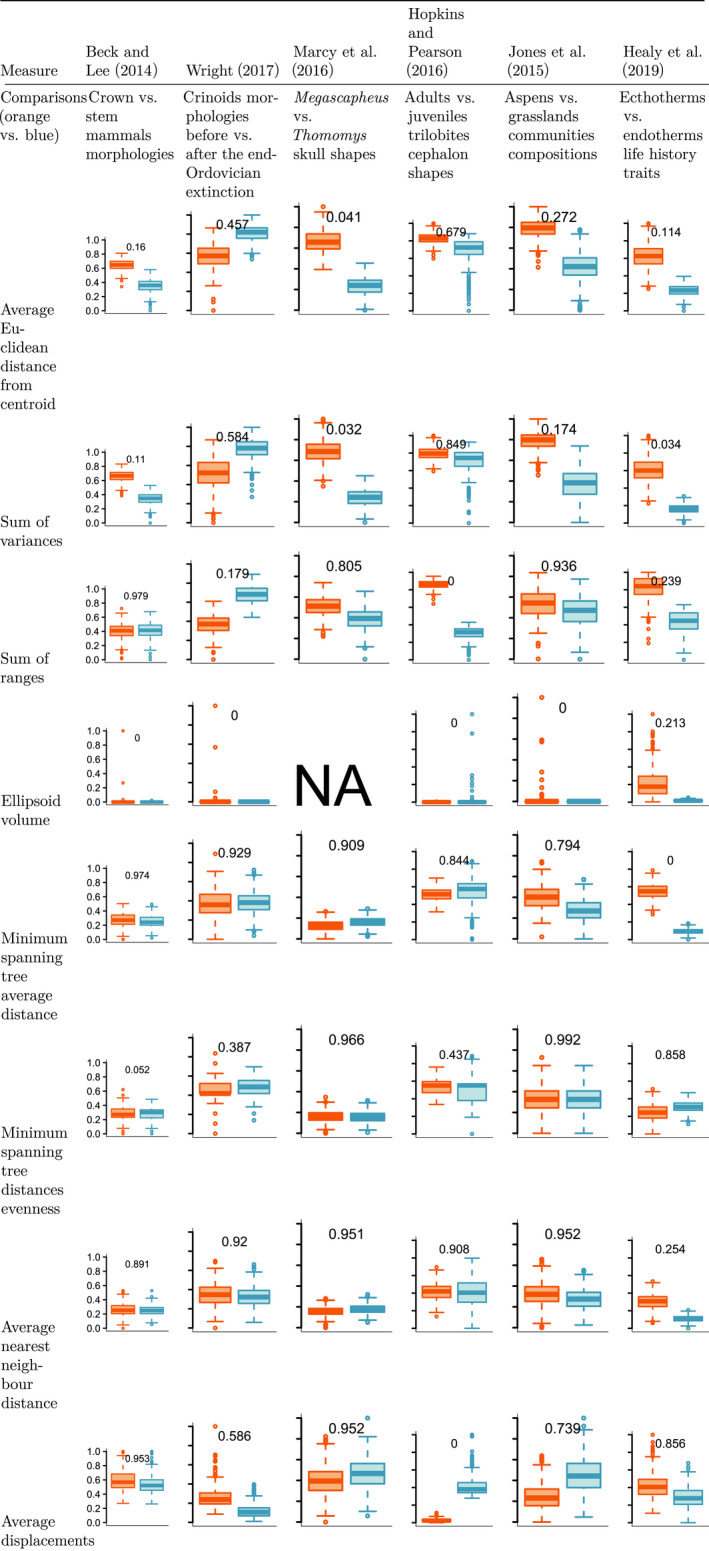
Comparisons of pairs of groups in different empirical trait spaces. NAs are used for cases where space occupancy could not be measured due to the curse of multidimensionality. The displayed values are the amount of overlap between both groups (Bhattacharrya Coefficient)

Note

NAs are used for cases where space occupancy could not be measured due to the curse of multidimensionality. The displayed values are the amount of overlap between both groups (Bhattacharrya Coefficient).

### 4.1 Measures comparisons

Measures within the same category of trait space occupancy (size, density, or position) do not have the same level of correlation with each other. For example, the average Euclidean distance from centroid (size) is highly correlated to the sum of variances (size – correlation of 0.99) and somewhat correlated with the minimum spanning tree average distance (density – correlation of 0.66) but poorly with the ellipsoid volume (size – correlation of 0.17) and the minimum spanning tree distances evenness (density – correlation of −0.05). Furthermore, the fact that we have such a range of correlations for normal distributions suggests that each measure can capture different summaries of space occupancy ranging from obvious differences (for measures not strongly correlated) to subtle ones (for measures strongly correlated).

### 4.2 Space shifting

Most measures capture no changes in space occupancy for the “null” (random) space reduction (in gray in Table 5). This is a desirable behavior for space occupancy measures since it will likely avoid false positive errors in studies that estimate biological processes from space occupancy patterns (e.g., convergence, Marcy et al., 2016; life history traits, Healy et al., 2019). However, the average nearest neighbor distance and the sum of ranges have a respectively positive and negative “null” median. In itself, this is not necessarily a negative property but it should be kept in mind that even random processes can increase or decrease these measures' values.

For changes in size, the sum of variances and the average Euclidean distance from centroid are good descriptors (Table 5). However, as illustrated in the 2D examples in Figure 2b only the blue change results (Table 5) should not result in a direct change in overall size because the trait space is merely “hollowed” out. That said, “hollowing” is harder to conceptualize in many dimensions and the measures can still be interpreted for comparing groups (orange has a smaller volume than blue).

The average nearest neighbor distance and the minimum spanning tree average distance consistently detect changes in density with more precision for low density trait spaces (in blue in Table 5). However, we can observe some degree of correlation between the changes in density and the changes in size for most measure picking either signal. This could be due to the use of normally distributed spaces where a change in density often leads to a change in size. This is not necessarily the case with empirical data.

Regarding the changes in position, only the average displacement measure seems able to distinguish between a random change and a displacement of the trait space (Table 5). However, the average displacement measure does not distinguish between positive or negative displacement: This might be due to the inherent complexity of *position* in a multidimensional trait space.

### 4.3 Empirical examples

Although most differences are fairly consistent within each dataset with one group having a higher space occupancy score than the other for multiple measures, this difference can be more or less pronounced within each dataset (ranging from no to nearly full overlap – BC $\in \left(0;\;0.995\right)$ ) and sometimes even reversed. This indicates that opposite conclusions can be drawn from a dataset depending on which space occupancy measure is considered. For example, in Wright (2017), crinoids after the Ordovician mass extinction have a higher median measure value for all measures but for the average displacement. These differences depending on the measures are also more pronounced in the empirical datasets where the observations per group are unequal (Healy et al., 2019; Hopkins & Pearson, 2016).

### 4.4 Caveats

While our simulations are useful to illustrate the behavior of diverse space occupancy measures, they have several caveats. First, the simulated observations in the trait spaces are independent. This is not the case in biology where observations can be spatially (Jones et al., 2015) or phylogenetically correlated (e.g., Beck & Lee, 2014). Second, the algorithm used to reduce the trait spaces might not always accurately reflect changes. This might favor some specific measures over others, in particular for the changes in density that modify the nearest neighbor density rather than changing the global density. This algorithmic choice was made in order to not confound changes in density along with changes in size. However, the results presented here probably capture the general behavior of each measure since results are consistent between the simulated and empirical analysis.

Furthermore, we did not take into account the effect of sampling on space occupancy measurements (but see additional results with 80% and 20% space reduction in the Appendix S4). In fact, sampling has been previously shown to have an effect on measurements depending on range or volumes (e.g., the sum of ranges or the hypervolume, Ciampaglio et al., 2001; Mammola, 2019). This effect is especially expected to be acerbated in macroevolutionary studies when using the fossil record (Brocklehurst, Kammerer, & Fröbisch, 2013) but can be tackled using rarefaction and bootstrapping techniques (Guillerme, 2018).

### 4.5 Using moms to choose the appropriate measurements Using moms to choose the appropriate measurements

Therefore, we propose the https://tguillerme.shinyapps.io/moms/moms shiny app to allow workers to help them choose their set of space occupancy measurements (and test the caveats mentioned above). Moms is an online graphical user interface to help analyze multidimensional data. It allows users to upload their dataset of interest (or simulate one with specific parameters) and measure space occupancy using a variety of implemented measures (namely, but not only, the ones used in this study). Furthermore, the package allows simulation of shifts in trait space occupancy as also presented in this paper to test whether some measures capture specific changes in space. However, moms is not a tool for analyzing multidimensional data per se but rather for helping workers to chose the space occupancy measure most appropriated to their data and question. To run multidimensional analysis, we suggest using dedicated R packages (such as – but not limited to: Oksanen et al. (2007), Bonhomme et al. (2014), Cardoso, Rigal, and Carvalho (2015), Guillerme (2018).

## 5 CONCLUSIONS

We insist that although no measure is objectively better than the next one, some can be more problematic than other in specific contexts. For example, the results for the sum of ranges, minimum spanning tree average distances, and to a lesser extent average nearest neighbor distances produced results in the reduced space often similar to the randomly reduced spaces (Table 5). This does not make them “bad” measures but rather heavily context dependent. Regardless, we believe that workers should identify the most appropriate measures based on their trait space properties as well as their specific biological question. We believe this could be fostered by following these several suggestions:

First, we suggest using multiple measures to tackle different aspects of the trait space. This follows the same logical thinking that the mean might not be sufficient to describe a distribution (e.g., the variance might be a good additional descriptor). Although using multiple measures is not uncommon in macroevolutionary studies (e.g., Halliday & Goswami, 2016) or in ecology (Mammola, 2019), they often do no cover more than one of the three categories of trait space measures (but see the recent work of Carmona, Bello, Mason, & Lepš, 2019 and Mammola & Cardoso, 2020).

Second, we suggest selecting the measures that best address the biological question at hand. If one studies an adaptive radiation in a group of organisms, it is worth thinking what would be the expected null model: would the group's size increase (radiation in all directions), would it increase in density (niche specialization) or would it shift in position (radiation into a new set of niches)?

Third, we suggest not naming measures after the biological aspect they describe which can be vague (e.g., “disparity” or “functional dispersion”) but rather after what they are measuring and why (e.g., “we used sum of ranges to measure the space size”). We believe this will support both a clearer understanding of what *is* measured as well as better communication between ecology and evolution research where measures can be similar but have different names.

Multidimensional analyses have been acknowledged as essential tools in modern biology but they can often be counter‐intuitive (Bellman, 1957). It is thus crucial to accurately describe patterns in multidimensional trait spaces to be able to link them to biological processes. When summarizing trait spaces, it is important to remember that a pattern captured by a specific space occupancy measure is often dependent on the properties of the space and of the particular biological question of interest. We believe that having a clearer understanding of both the properties of the trait space and the associated space occupancy measures (e.g., using https://tguillerme.shinyapps.io/moms/moms) as well as using novel space occupancy measures to answer specific questions will be of great use to study biological processes in a multidimensional world.

## CONFLICT OF INTEREST

None declared.

## AUTHOR CONTRIBUTIONS

**Thomas Guillerme:** Conceptualization (lead); Formal analysis (lead); Methodology (lead); Software (lead); Writing‐original draft (lead); Writing‐review & editing (lead). **Ariel Marcy:** Conceptualization (equal); Formal analysis (equal); Methodology (equal); Writing‐original draft (equal); Writing‐review & editing (equal). **Mark N Puttick:** Conceptualization (equal); Formal analysis (equal); Methodology (equal); Writing‐original draft (equal); Writing‐review & editing (equal). **Vera Weisbecker:** Conceptualization (equal); Funding acquisition (lead); Methodology (equal); Writing‐original draft (equal); Writing‐review & editing (equal).

### Open Research Badges

This article has been awarded Open Data, Open Materials, Preregistered Badges. All materials and data are publicly accessible via the Open Science Framework at https://doi.org/10.6084/m9.figshare.9943181.v1, https://doi.org/10.6084/m9.figshare.9943181.v1; https://zenodo.org/record/3818337#.Xra9TMZ7lTY, https://doi.org/10.5281/zenodo.3818337; https://github.com/TGuillerme/moms or https://zenodo.org/record/3818337#.Xra9TMZ7lTY, https://doi.org/10.5281/zenodo.3818337.

## Supporting information

App S1

App S2

App S3

App S4

## Data Availability

The raw empirical data are available from the original papers (Beck & Lee, 2014; Healy et al., 2019; Hopkins & Pearson, 2016; Jones et al., 2015; Marcy et al., 2016; Wright, 2017). The subsets of the empirical data used in this analysis are available on figshare https://doi.org/10.6084/m9.figshare.9943181.v1: 10.6084/m9.figshare.9943181.v1. The modified empirical data are available in the package accompanying this manuscript (data(moms::demo_data)). This manuscript (including the figures, tables and Appendices [Link], [Link], [Link], [Link]) is repeatable and reproducible by compiling the vignette of the https://github/TGuillerme/momsGitHub moms R package.
