# Supplementary material for: Shifting spaces: Which disparity or dissimilarity measurement best summarize occupancy in multidimensional spaces?
Source: Ecol Evol. 2020 Jul 5;10(14):7261–75. doi: 10.1002/ece3.6452 (PMC7391566; doi:10.1002/ece3.6452)
Supplement: Supplementary file 1 — App S1 [file ECE3-10-7261-s001.pdf]

Shifting spaces: which disparity or dissimilarity measurement best summarise occupancy in multidimensional spaces?

Thomas Guillerme, Mark N. Puttick, Ariel E. Marcy, Vera Weisbecker

2020-02-12

Supplementary material 1: simulations

```
## Loading required package: knitr

## Loading required package: rmarkdown

## Loading required package: dispRity

## Registered S3 method overwritten by 'geiger':
##   method            from
##   unique.multiPhylo ape

## Loading required package: moms
```

Algorithm for selecting the parameters to reduce the space (*radius*, *displacement*, *density*)

We used a recursive algorithm for selecting the parameter that removes $P = 50\%$ elements.

1. Select a random reduction parameters R.
2. Remove elements from the space using R resulting in P' removed elements. If the remaining number of elements is equal to the required proportion P ; exit the algorithm; Else go to 3.
3. Get the different between the proportion of removed elements P' and P. If the difference is positive set the increment parameter R to $R = 1.1 \times R$, then go to 2. Else set $R = 0.9 \times R$, then go to 2.

The algorithm is implemented in the `optimise.parameter` function in reduce.space_fun.R.

Simulation data

This section contains all the results and calculations (and plots them). The whole analysis takes approximatively 30 minutes to run (single core 2.2 GHz).

Measurements analysed

The measurements analysed are the Average Euclidean distance from centroid, Sum of variances, Sum of ranges, Ellipsoid volume, Minimum spanning tree average distance, Minimum spanning tree distances evenness, Average nearest neighbour distance, Average displacements.

Generating the data

This section runs and summarises the simulations. The simulations are run silently in this markdown file (and are not printed on the compiled version of this document). The code is available within this R markdown file (un-compiled).

```
## Set the overall number of replicates
n_replicates <- 20

## Set the overall number of elements
#elements <- function() sample(20:200) #possibility to use a variable number of elements
elements <- function() 200

## Simulation seed
set.seed(42)
```

```
## List of distributions
distributions_list <- list(
    "unifor" = list(distribution = runif, arguments = list(list("min" = -0.5,
                                                                "max" = 0.5))),
    "normal" = list(distribution = rnorm),
    "random" = list(distribution = "random"),
    "unicor" = list(distribution = runif, arguments = list(list("min" = -0.5,
                                                                "max" = 0.5)),
                    cor.matrix = "random"),
    "norcor" = list(distribution = rnorm, cor.matrix = "random"),
    "pcalik" = list(distribution = rnorm, scree = "lognormal"),
    "pcolik" = list(distribution = rnorm, scree = "normal")
    )

## Generate all 13 spaces
all_spaces <- list(uniform3 = space.simulation(distributions_list$unifor,
                                              dimensions = 3,
                                              elements = elements(),
                                              replicates = n_replicates),
                   uniform15 = space.simulation(distributions_list$unifor,
                                              dimensions = 15,
                                              elements = elements(),
                                              replicates = n_replicates),
                   uniform50 = space.simulation(distributions_list$unifor,
                                              dimensions = 50,
                                              elements = elements(),
                                              replicates = n_replicates),
                   uniform100 = space.simulation(distributions_list$unifor,
                                              dimensions = 100,
                                              elements = elements(),
                                              replicates = n_replicates),
                   uniform150 = space.simulation(distributions_list$unifor,
                                              dimensions = 150,
                                              elements = elements(),
                                              replicates = n_replicates),
                   uniformc50 = space.simulation(distributions_list$unicor,
                                              dimensions = 50,
                                              elements = elements(),
                                              replicates = n_replicates),
```

```r
                   normal3 = space.simulation(distributions_list$normal,
                                              dimensions = 3,
                                              elements = elements(),
                                              replicates = n_replicates),
                   normal15 = space.simulation(distributions_list$normal,
                                              dimensions = 15,
                                              elements = elements(),
                                              replicates = n_replicates),
                   normal50 = space.simulation(distributions_list$normal,
                                              dimensions = 50,
                                              elements = elements(),
                                              replicates = n_replicates),
                   uniform100 = space.simulation(distributions_list$unifor,
                                              dimensions = 100,
                                              elements = elements(),
                                              replicates = n_replicates),
                   normal150 = space.simulation(distributions_list$normal,
                                              dimensions = 150,
                                              elements = elements(),
                                              replicates = n_replicates),
                   normalc50 = space.simulation(distributions_list$norcor,
                                              dimensions = 50,
                                              elements = elements(),
                                              replicates = n_replicates),
                   random50 = space.simulation(distributions_list$random,
                                              dimensions = 50,
                                              elements = elements(),
                                              replicates = n_replicates),
                   pca_like = space.simulation(distributions_list$pcalik,
                                              dimensions = 50,
                                              elements = elements(),
                                              replicates = n_replicates),
                   pco_like = space.simulation(distributions_list$pcolik,
                                              dimensions = 50,
                                              elements = elements(),
                                              replicates = n_replicates))

#TG: Warnings are due to inexact correlations
```

```r
## Shifting each space
shift_groups <- lapply(all_spaces, lapply, shift.group.simulation, remove = 0.5)
```

```r
## Measuring disparity
remove_05 <- mapply(metrics.simulation, all_spaces, shift_groups,
                  MoreArgs = list(metrics = metrics_list, rare.dim = NULL,
                                  verbose = TRUE),
                  SIMPLIFY = FALSE)

## Saving the results
save.results(remove_05)
```
