# Supplementary material for: Shifting spaces: Which disparity or dissimilarity measurement best summarize occupancy in multidimensional spaces?
Source: Ecol Evol. 2020 Jul 5;10(14):7261–75. doi: 10.1002/ece3.6452 (PMC7391566; doi:10.1002/ece3.6452)
Supplement: Supplementary file 2 — App S2 [file ECE3-10-7261-s002.pdf]

Shifting spaces: which disparity or dissimilarity measurement best summarise occupancy in multidimensional spaces?

Thomas Guillerme, Mark N. Puttick, Ariel E. Marcy, Vera Weisbecker

2020-02-12

Supplementary material 2: empirical data

```
## Loading required package: knitr

## Loading required package: rmarkdown

## Loading required package: dispRity

## Registered S3 method overwritten by 'geiger':
##   method            from
##   unique.multiPhylo ape

## Loading required package: geomorph

## Loading required package: RRPP

## Loading required package: rgl

##
## Attaching package: 'geomorph'

## The following object is masked from 'package:dispRity':
##
##     combine.subsets

## Loading required package: moms
```

Empirical data

Creating the trait spaces

The following sections contain the methodology and the code to generate the six empirical trait-spaces analysed in the paper. The protocol for creating each trait-space is detailed below.

Palaeontological (discrete) data

For the discrete morphological data, we calculated a distance matrix from the original discrete morphological matrices from Beck and Lee (2014) and Wright (2017) using the maximum observable distance (Lloyd 2016, Lehmann et al.) that we then ordinated using a classical multidimensional scaling (MDS; (Torgerson 1965, gower1966, cailliez1983)) by adding a constant **c*** to make the distances Euclidean ($d_{MORD} + c* = d_{Euclidean}; Cailliez (1983)). This resulted in $\boldsymbol{k}$ eigenvectors for each matrix where $\boldsymbol{k}$ is equal to $n - 1$ taxa.

```r
## Creating the trait-spaces
beck_traitspace <- Claddis.ordination("../data/raw/Beck2014.nex", distance = "MORD")
wright_traitspace <- Claddis.ordination("../data/raw/Wright2017.nex", distance = "MORD")
```

For both datasets we created two groups, the crown versus the stem mammals for Beck and Lee (2014) and the crinoids occuring before and after the Ordovician-Silurian boundary (455-430 million years ago - Mya).

```r
## Loading the tree from Beck and Lee 2014
beck_tree <- read.nexus("../data/raw/Beck2014.tre")

## Selecting the crown and stem mammals
groups_beck <- crown.stem(beck_tree)

## Adding the four stem mammals not in the tree
missing_stem <- rownames(beck_traitspace)[which(is.na(match(rownames(beck_traitspace),
                                                            beck_tree$tip.label)))]
groups_beck$stem <- c(groups_beck$stem, missing_stem)

## Loading the first and last occurence data from Wright 2017
wright_ages <- read.csv("../data/raw/Wright2017.csv")

## Separating taxa that went extinct half way through the Ordovician-Silurian extinction
## (442.5 million years ago)
groups_wright <- ifelse(wright_ages$LAD < 422.5, TRUE, FALSE)
groups_wright <- list(#"all" = as.character(wright_ages$species),
                      "before" = as.character(wright_ages$species[which(groups_wright)]),
                      "after" =  as.character(wright_ages$species[which(!groups_wright)]))

## Creating the dispRity objects
beck_traitspace <- custom.subsets(beck_traitspace, group = groups_beck)
wright_traitspace <- custom.subsets(wright_traitspace, group = groups_wright)

## Saving the data for the main analysis
palaeo <- list("beck" = beck_traitspace, "wright" = wright_traitspace)
save.results(palaeo)
```

Geometric morphometric (landmark) data

For the continuous geometric morphometric data, we performed a generalised Procrustes analysis (Adams and Otárola-Castillo 2013), using 2D (Marcy et al. 2016) or 3D (Hopkins and Pearson 2016) landmark coordinates sets (with semi-landmarks, i.e. sliders). We then ordinated the resulted Procrustes superimposed data using a principal component analysis (PCA; Hotelling 1933) and divided the data into two groups for each dataset (see below and in the main text).

Marcy et al. 2016

This dataset consists 2D landmarks for 552 specimens (25 fixed landmarks and 42 semi-landmark curves; Marcy et al. 2016). The dataset is then divided into two genera(Marcy et al. 2016).

```r
## Read in TPS coordinates
marcy_data <- readland.tps("../data/raw/Marcy2016.TPS", specID = "imageID",
                           warnmsg = FALSE)

## Remove duplicated points for ventral crania semi-landmarks (artefact of the landmarking
```

```
## process)
marcy_data <- marcy_data[-c(1, 2, 26, 29, 30, 41, 42, 45, 46, 51, 58, 60, 61, 68, 69, 78,
                            79, 85), , ]

## Read in semilandmark definitions from Marcy et al 2016
sliders <- read.csv("../data/raw/Marcy2016.csv", header = TRUE, colClasses = "integer")

## Get the specimens names
marcy_names <- strsplit(names(marcy_data[1, 1, ]), "_")

## Find non-unique specimens (they have a suffix, i.e. 7 elements)
duplicated <- ifelse(unlist(lapply(marcy_names, length)) == 7, TRUE, FALSE)

## Remove duplicated specimens
marcy_data <- marcy_data[, , !duplicated]
marcy_names <- marcy_names[!duplicated]

## Procrustes superimposition
marcy_gpa <- gpagen(marcy_data, Proj = TRUE, ProcD = TRUE, curves = sliders,
                    print.progress = FALSE)
```

Hopkins and Pearson 2016 (ontogenic sequences)

This dataset consists of 63 3D landmarks for 46 specimens (from 23 individuals, 3 of which have estimated landmarks, Hopkins and Pearson 2016). The data is then separated into juveniles and adults (based on the specimens centroids sizes; Hopkins and Pearson 2016).

```
## Read in TPS coordinates
hopkins_data <- readland.tps("../data/raw/Hopkins2016.tps", specID = "ID",
                             warnmsg = FALSE)
```

```
##
## Negative landmark coordinates have been identified and imported as such.
## If you want to treat them as NAs please set negNA = TRUE
```

```
## Remove duplicated points (repeated coordinates reported in Appendix 3 of original
## paper)
hopkins_data <- hopkins_data[-c(63, 67, 55, 65), , ]

## Estimate missing landmarks using TPS method (used in orignial paper)
hopkins_data <- estimate.missing(hopkins_data, "TPS")

## Procrustes superimposition
hopkins_gpa <- gpagen(hopkins_data, print.progress = FALSE)
```

Groups

We divided the dataset from Marcy et al. (2016) into two groups corresponding to the two genera: *Thomomys* and *Megascapheus*; and the dataset from Hopkins and Pearson (2016) into two groups, adult and juvenile based on the log centroid size threshold of 2.3 (i.e. juveniles have a log centroid size < 2.3; Hopkins and Pearson 2016).

```
## Selecting the generas (first element in the name list)
marcy_groups <- unlist(lapply(marcy_names, function(x) x[[1]]))
```

```r
## Renaming the generas
marcy_groups <- as.factor(ifelse(marcy_groups == "T", "Thomomys", "Megascapheus"))

## Creating the geomorph data.frames
marcy_data <- geomorph.data.frame(marcy_gpa, marcy_groups)

## Setting the threshold for distinguising adults/juveniles in the hopkins data
size_threshold <- 2.3

## Creating the groupins
hopkins_groups <- ifelse(log(hopkins_gpa$Csize) > size_threshold, "adult", "juvenile")

## Creating the geomorph data.frames
hopkins_data <- geomorph.data.frame(hopkins_gpa, hopkins_groups)
```

```r
## Creating the trait space
marcy_traitspace <- dispRity::geomorph.ordination(marcy_data)
hopkins_traitspace <- dispRity::geomorph.ordination(hopkins_data)

## Saving the data for the main analysis
gmm <- list("marcy" = marcy_traitspace, "hopkins" = hopkins_traitspace)
save.results(gmm)
```

Ecological data

Jones et al. 2015 data (species composition data)

For the Jones et al. (2015) dataset, we converted the spreadsheet "aspen & grass richness & abund" from the data from Jones et al. (2015) into a .csv file (now available in `/data/raw`). We then selected only the `wholeplot` entries and separated the studied plot between `aspen` and `grassland` and converted the species presence data into a presence/absence matrix. From this matrix we calculated a Jaccard distance matrix (Oksanen et al. 2007) that we then ordinated using a classical multidimensional scaling (MDS; (Torgerson 1965, cailliez1983)) by adding a constant **c*** to make the distances Euclidean ($d_{MORD} + c* = d_{Euclidean}; Cailliez (1983)). This resulted in $\boldsymbol{k}$ eigenvectors for each matrix where $\boldsymbol{k}$ is equal to $n-1$ field sites.

```r
## Reading the community composition data from Jones et al 2015
jones_communities <- read.csv("../data/raw/Jones2015.csv", stringsAsFactors = FALSE)

## Selecting only the whole plots and the plot number, habitat and species
jones_whole_com <- jones_communities[which(jones_communities$mptype == "wholeplot"),
                                     c(1,2,3)]

## Separate each plot into aspen/grassland
jones_whole_com$plot <- paste(jones_whole_com[,1], jones_whole_com$habitat, sep = ".")

## Making the community matrix
plot_names <- unique(jones_whole_com$plot)
sp_codes <- unique(jones_whole_com$species.code)
community <- matrix(NA, nrow = length(plot_names),
                        ncol = length(sp_codes),
                        dimnames = list(plot_names, sp_codes))
```

```r
## Populate the matrix
for(one_plot in rownames(community)) {
    ## Get the community composition
    composition <- match(colnames(community),
                         jones_whole_com[which(jones_whole_com$plot == one_plot), 3])
    ## Fill the plot row with presence/absence data
    community[which(rownames(community) == one_plot), ] <- ifelse(is.na(composition),
                                                                   0, 1)
}

## Calculate the distance matrix
distance_matrix <- as.matrix(vegan::vegdist(community, method = "jaccard"))

## Ordinating the data
jones_ordination <- stats::cmdscale(distance_matrix,
                                    k = ncol(distance_matrix)- 1, add = TRUE)$points
```

Healy et al. 2019 data (life history traits data)

For the Healy et al. (2019) dataset, we used a processed version of the data available on the GitHub repository associated to the paper.

The procedure from Healy et al. (2019) consists of analysing matrix population models and extracting life history traits (survival rate, age at first reproduction, generation time, reproduction rate, life expectancy post sexual maturity and reproduction spread). To take into account the effect of body mass and phylogeny, Healy et al. (2019) used the residual of the six traits from general linear mixed models (Hadfield and others 2010; Guillerme and Healy 2014). See Healy et al. (2019) for the exact description of the procedure.

We used the PCA_main_2019_may_28 script to obtain the data corrected for body mass and phylogeny with six variables: the survival residuals (`"SD_mort"`), the age at first reproduction (`"La_r"`), the generation time (`"gen_r"`), the mean reproductive rate (`"M_repo"`), the life expectancy post sexual maturity (`"M_suv"`) and the spread of reproduction (`"gini_r"`).

```r
## Load the processed data
healy_data <- read.csv("../data/raw/Healy2019.csv")

## Cleaning up the data
healy_traits <- healy_data[, -c(1, 8, 9)]

## Ordinating the traits
healy_ordination <- prcomp(healy_traits)$x
```

Making the ecological trait-space groups

We separated the Jones et al. (2015) dataset into `aspen` and `grassland` groups and we separated the Healy et al. (2019) dataset into `ectotherms` `endotherms`:

```r
## Making the groups
jones_groups <- list("aspen" = grep("aspen", rownames(jones_ordination)),
                     "grassland" = grep("grassland", rownames(jones_ordination)))
healy_groups <- list("ectotherms" = which(healy_data[, 9] == "ecto"),
                     "endotherms" = which(healy_data[, 9] == "endo"))
```

```r
## Making the dispRity objects
jones_traitspace <- custom.subsets(jones_ordination, group = jones_groups)
healy_traitspace <- custom.subsets(healy_ordination, group = healy_groups)

## Saving the data for the main analysis
ecol <- list("jones" = jones_traitspace, "healy" = healy_traitspace)
save.results(ecol)
```

Exporting the datasets for shiny

```r
## The list of datasets
demo_data <- c(palaeo, gmm, ecol)
save(demo_data, file = "../data/demo_data.rda")
```

Running the disparity calculations

```r
## Loading the list of metrics
source("list.of.metrics.R")
```

```r
## Loading the data
trait_spaces <- demo_data

## Measuring space occupancy for all spaces and all metrics
apply.disparity <- function(space, metrics) {
    lapply(metrics, function(metric, space) dispRity(boot.matrix(space, bootstraps = 500),
                                                      metric), space)
}
empirical_results <- lapply(trait_spaces, apply.disparity, metrics_list)

## Results list
save.results(empirical_results)
```

References

Adams D.C., Otárola-Castillo E. 2013. Geomorph: An R package for the collection and analysis of geometric morphometric shape data. Methods in Ecology and Evolution. 4:393–399.

Beck R.M.D., Lee M.S.Y. 2014. Ancient dates or accelerated rates? Morphological clocks and the antiquity of placental mammals. Proceedings of the Royal Society B: Biological Sciences. 281:20141278.

Cailliez F. 1983. The analytical solution of the additive constant problem. Psychometrika. 48:305–308.

Guillerme T., Healy K. 2014. mulTree: a package for running MCMCglmm analysis on multiple trees. Zenodo.

Hadfield J.D., others. 2010. MCMC methods for multi-response generalized linear mixed models: The mcmcglmm r package. Journal of Statistical Software. 33:1–22.

Healy K., Ezard T.H.G., Jones O.R., Salguero-G'omez R., Buckley Y.M. 2019. Animal life history is shaped by the pace of life and the distribution of age-specific mortality and reproduction. Nature Ecology & Evolution.

2397-334X.

Hopkins M., Pearson K. 2016. Non-linear ontogenetic shape change in cryptolithus tesselatus (trilobita) using three-dimensional geometric morphometrics. Palaeontologia Electronica. 19:1–54.

Hotelling H. 1933. Analysis of a complex of statistical variables into principal components. Journal of educational psychology. 24:417.

Jones N.T., Germain R.M., Grainger T.N., Hall A.M., Baldwin L., Gilbert B. 2015. Dispersal mode mediates the effect of patch size and patch connectivity on metacommunity diversity. Journal of Ecology. 103:935–944.

Lehmann O.E.R., Ezcurra M.D., Butler R.J., Lloyd G.T. Biases with the generalized euclidean distance measure in disparity analyses with high levels of missing data. Palaeontology. 0.

Lloyd G.T. 2016. Estimating morphological diversity and tempo with discrete character-taxon matrices: Implementation, challenges, progress, and future directions. Biological Journal of the Linnean Society. 118:131–151.

Marcy A.E., Hadly E.A., Sherratt E., Garland K., Weisbecker V. 2016. Getting a head in hard soils: Convergent skull evolution and divergent allometric patterns explain shape variation in a highly diverse genus of pocket gophers (thomomys). BMC evolutionary biology. 16:207.

Oksanen J., Kindt R., Legendre P., O'Hara B., Stevens M.H.H., Oksanen M.J., Suggests M. 2007. The vegan package. Community ecology package. 10:631–637.

Torgerson W.S. 1965. Multidimensional scaling of similarity. Psychometrika. 30:379–393.

Wright D.F. 2017. Phenotypic innovation and adaptive constraints in the evolutionary radiation of palaeozoic crinoids. Scientific Reports. 7:13745.
