# Supplementary material for: Shifting spaces: Which disparity or dissimilarity measurement best summarize occupancy in multidimensional spaces?
Source: Ecol Evol. 2020 Jul 5;10(14):7261–75. doi: 10.1002/ece3.6452 (PMC7391566; doi:10.1002/ece3.6452)
Supplement: Supplementary file 3 — App S3 [file ECE3-10-7261-s003.pdf]

Shifting spaces: which disparity or dissimilarity measurement best summarise occupancy in multidimensional spaces?

Thomas Guillerme, Mark N. Puttick, Ariel E. Marcy, Vera Weisbecker

2020-02-12

Supplementary material 3: supplementary results

ANOVA tables

Table 1: Full ANOVA results

metric		Df	Sum Sq	Mean Sq	F value	Pr(>F)
func.disp	all spaces	14	6.056	0.433	1.011	0.439
	Residuals	2085	892.020	0.428	NA	NA
sum.var	all spaces	14	10.254	0.732	1.622	0.066
	Residuals	2085	941.398	0.452	NA	NA
sum.range	all spaces	14	31.924	2.280	22.356	0.000
	Residuals	2085	212.674	0.102	NA	NA
ellips.vol	all spaces	14	42.360	3.026	13.093	0.000
	Residuals	2085	481.831	0.231	NA	NA
span.tree	all spaces	14	5.811	0.415	1.613	0.068
	Residuals	2085	536.551	0.257	NA	NA
func.eve	all spaces	14	69.619	4.973	22.855	0.000
	Residuals	2085	453.657	0.218	NA	NA
ave.neigh	all spaces	14	6.429	0.459	1.576	0.078
	Residuals	2085	607.543	0.291	NA	NA
av.displa	all spaces	14	39.188	2.799	22.580	0.000
	Residuals	2085	258.470	0.124	NA	NA

Table 2: ANOVA results for the dimension effect

metric		Df	Sum Sq	Mean Sq	F value	Pr(>F)
func.disp	dimensions	8	1.056	0.132	0.322	0.958
	Residuals	1251	513.546	0.411	NA	NA
sum.var	dimensions	8	1.651	0.206	0.478	0.873
	Residuals	1251	540.336	0.432	NA	NA
sum.range	dimensions	8	23.376	2.922	32.307	0.000
	Residuals	1251	113.146	0.090	NA	NA
ellips.vol	dimensions	8	29.790	3.724	13.486	0.000
	Residuals	1251	345.432	0.276	NA	NA
span.tree	dimensions	8	1.923	0.240	0.998	0.435
	Residuals	1251	301.181	0.241	NA	NA
func.eve	dimensions	8	46.179	5.772	29.358	0.000
	Residuals	1251	245.970	0.197	NA	NA
ave.neigh	dimensions	8	1.733	0.217	0.773	0.626

metric		Df	Sum Sq	Mean Sq	F value	Pr(>F)
	Residuals	1251	350.289	0.280	NA	NA
av.displa	dimensions	8	23.095	2.887	26.829	0.000
	Residuals	1251	134.610	0.108	NA	NA

Table 3: ANOVA results for the dimension effect (in uniform spaces)

metric		Df	Sum Sq	Mean Sq	F value	Pr(>F)
func.disp	dimensions (uniform)	4	0.468	0.117	0.315	0.868
	Residuals	695	258.379	0.372	NA	NA
sum.var	dimensions (uniform)	4	1.101	0.275	0.701	0.592
	Residuals	695	273.075	0.393	NA	NA
sum.range	dimensions (uniform)	4	15.826	3.957	46.782	0.000
	Residuals	695	58.779	0.085	NA	NA
ellips.vol	dimensions (uniform)	4	20.784	5.196	19.936	0.000
	Residuals	695	181.133	0.261	NA	NA
span.tree	dimensions (uniform)	4	0.601	0.150	0.712	0.584
	Residuals	695	146.517	0.211	NA	NA
func.eve	dimensions (uniform)	4	30.954	7.739	37.525	0.000
	Residuals	695	143.326	0.206	NA	NA
ave.neigh	dimensions (uniform)	4	0.344	0.086	0.331	0.857
	Residuals	695	180.497	0.260	NA	NA
av.displa	dimensions (uniform)	4	11.435	2.859	26.265	0.000
	Residuals	695	75.647	0.109	NA	NA

Table 4: ANOVA results for the dimension effect (in normal spaces)

metric		Df	Sum Sq	Mean Sq	F value	Pr(>F)
func.disp	dimensions (normal)	3	0.206	0.069	0.149	0.930
	Residuals	556	255.168	0.459	NA	NA
sum.var	dimensions (normal)	3	0.508	0.169	0.352	0.788
	Residuals	556	267.261	0.481	NA	NA
sum.range	dimensions (normal)	3	6.627	2.209	22.591	0.000
	Residuals	556	54.367	0.098	NA	NA
ellips.vol	dimensions (normal)	3	8.678	2.893	9.789	0.000
	Residuals	556	164.300	0.296	NA	NA
span.tree	dimensions (normal)	3	0.123	0.041	0.147	0.932
	Residuals	556	154.664	0.278	NA	NA
func.eve	dimensions (normal)	3	1.550	0.517	2.799	0.039
	Residuals	556	102.644	0.185	NA	NA
ave.neigh	dimensions (normal)	3	0.717	0.239	0.783	0.504
	Residuals	556	169.792	0.305	NA	NA
av.displa	dimensions (normal)	3	11.570	3.857	36.368	0.000
	Residuals	556	58.963	0.106	NA	NA

metric		Df	Sum Sq	Mean Sq	F value	Pr(>F)

Table 5: ANOVA results for the space distribution types

metric		Df	Sum Sq	Mean Sq	F value	Pr(>F)
func.disp	space type	4	1.743	0.436	0.924	0.449
	Residuals	695	327.753	0.472	NA	NA
sum.var	space type	4	2.546	0.637	1.285	0.274
	Residuals	695	344.358	0.495	NA	NA
sum.range	space type	4	4.763	1.191	11.119	0.000
	Residuals	695	74.433	0.107	NA	NA
ellips.vol	space type	4	7.102	1.776	7.215	0.000
	Residuals	695	171.023	0.246	NA	NA
span.tree	space type	4	1.416	0.354	1.162	0.326
	Residuals	695	211.696	0.305	NA	NA
func.eve	space type	4	6.742	1.685	8.152	0.000
	Residuals	695	143.693	0.207	NA	NA
ave.neigh	space type	4	1.981	0.495	1.478	0.207
	Residuals	695	232.839	0.335	NA	NA
av.displa	space type	4	5.856	1.464	10.742	0.000
	Residuals	695	94.727	0.136	NA	NA
