# Supplementary material for: Shifting spaces: Which disparity or dissimilarity measurement best summarize occupancy in multidimensional spaces?
Source: Ecol Evol. 2020 Jul 5;10(14):7261–75. doi: 10.1002/ece3.6452 (PMC7391566; doi:10.1002/ece3.6452)
Supplement: Supplementary file 4 — App S4 [file ECE3-10-7261-s004.pdf]

Shifting spaces: which disparity or dissimilarity measurement best summarise occupancy in multidimensional spaces?

Thomas Guillerme, Mark N. Puttick, Ariel E. Marcy, Vera Weisbecker

2020-04-27

Supplementary material 4: full results

```
## Loading required package: knitr
```

```
## Loading required package: rmarkdown
```

```
## Loading required package: dispRity
```

```
## Registered S3 methods overwritten by 'geiger':
##   method            from
##   logLik.gfit       phytools
##   unique.multiPhylo ape
```

```
## Loading required package: moms
```

Simulation data

This section contains all the results and calculations (and plots them). The whole analysis takes approximatively 30 minutes to run (single core 2.2 GHz).

Measurements analysed

The measurements analysed are the Average squared pairwise distance (dtt::dtt), Average pairwise manhattan distance (dtt::dtt), Median pairwise distance, Median pairwise manhattan distance, Procrustes variances (geomorph::morpho.disparity), Median distance from centroid, Median manhattan distance from centroid, Sum of variances, Sum of ranges, Sum of quantiles, Product of variances, Product of ranges, Product of quantiles, Ellipsoid volume, nBall volume, Minimum spanning tree average length, Average minimum neighbours distance, Average minimum neighbours manhattan distance, Function diversity, Functional evenness, Functional dispersion, Average displacement, Average manhattan displacement, Median distance from centre, Median manhattan distance from centre.

Running the simulations for the 25 metrics

Running the empirical data for the 25 metrics

Load all the data

```
## Load all the data
all_metrics_remove_02 <- load.results("all_metrics_remove_02")
all_metrics_remove_05 <- load.results("all_metrics_remove_05")
```

```r
all_metrics_remove_08 <- load.results("all_metrics_remove_08")
all_metrics_empirical_results <- load.results("all_metrics_empirical_results")
```

Measurements pairwise comparisons

```r
## getting the results to loop through (independent of the removal)
results_pairwise <- all_metrics_remove_02

## Plotting the pairwise results
for(metric_ID in 1:length(all_metrics_names)) {
  pairwise.plot(results_pairwise, scale = TRUE, type = "base", plot = metric_ID)
  cat(paste0(all_metrics_names[metric_ID], "\n"))
}
```

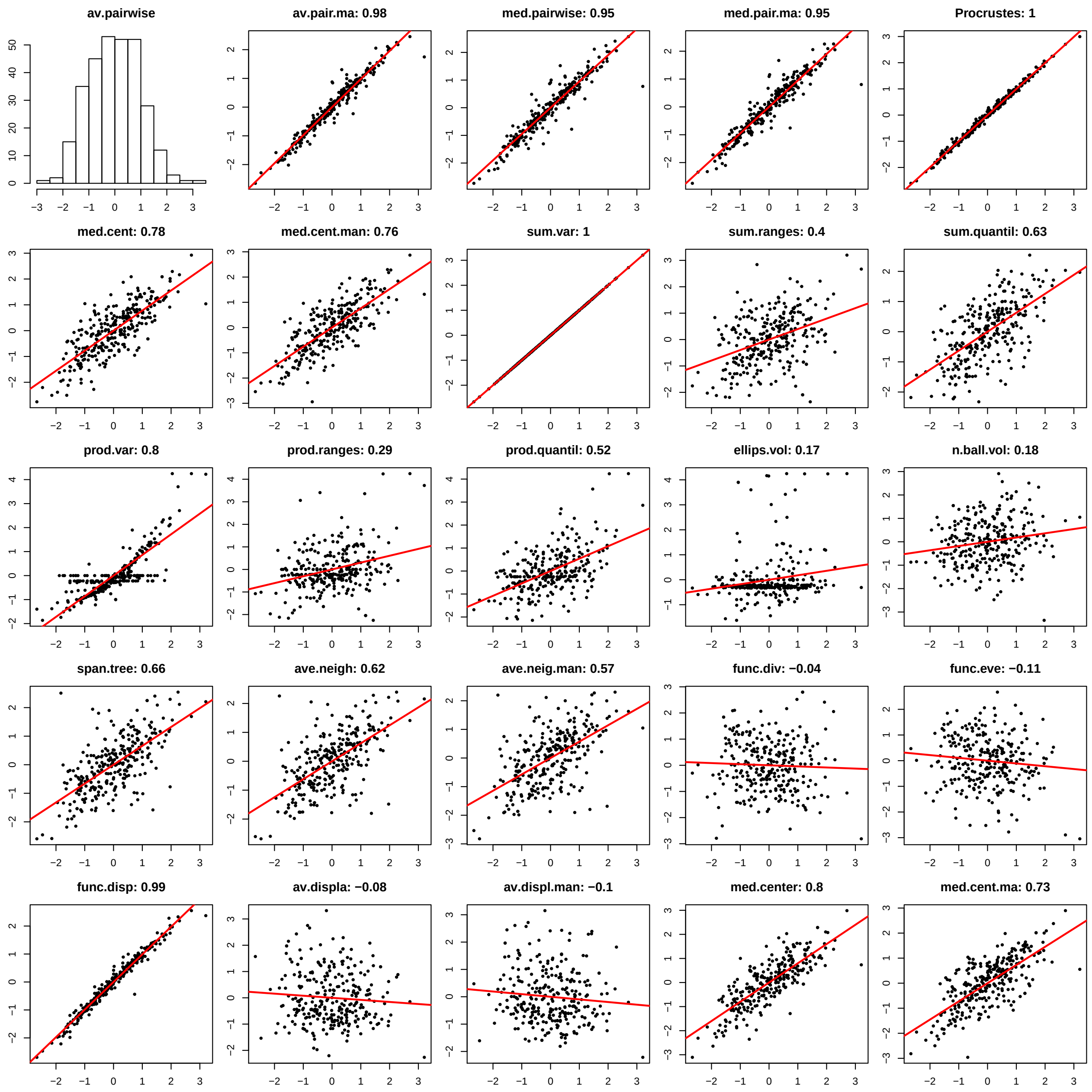


```
## Average squared pairwise distance (dtt::dtt)
```

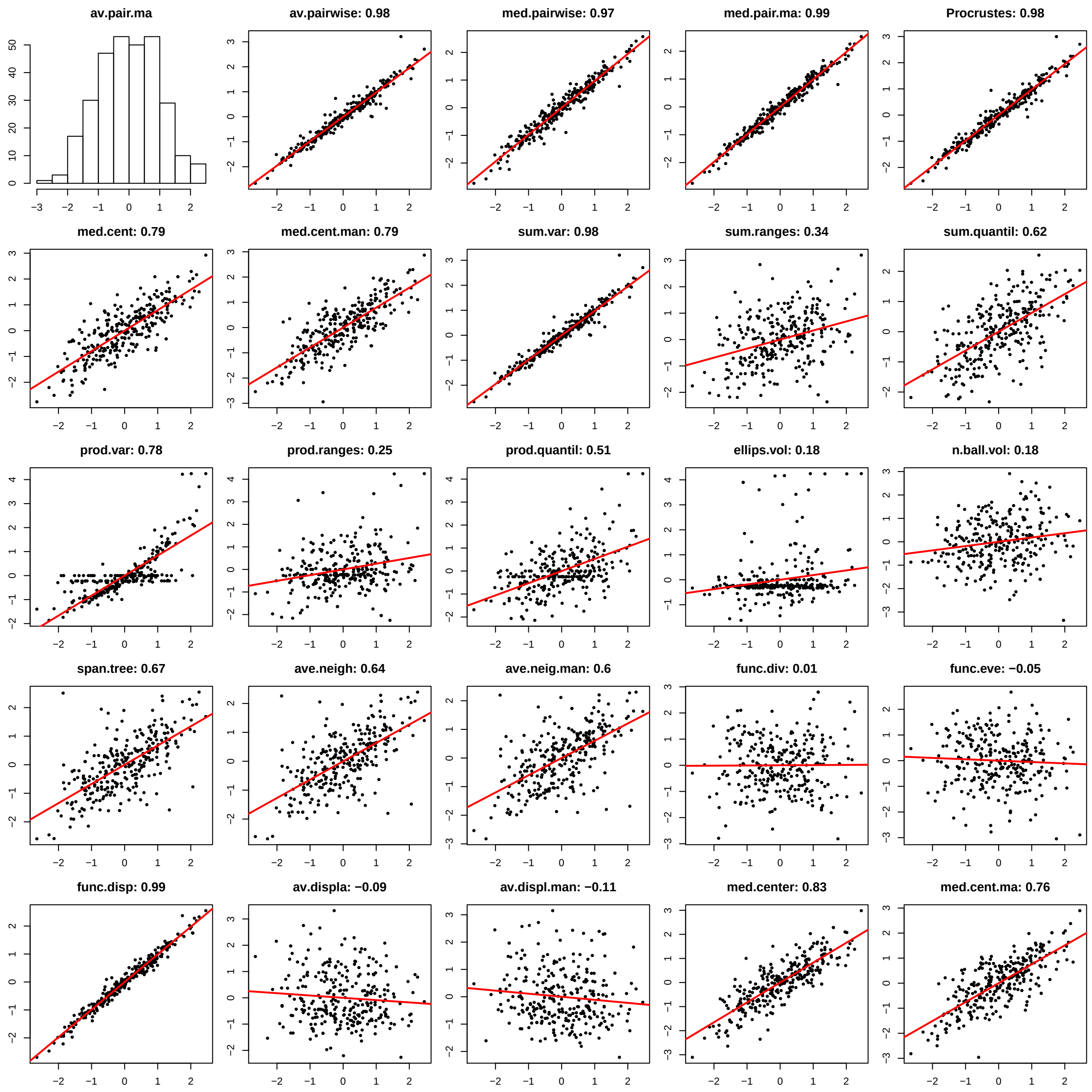


```
## Average pairwise manhattan distance (dtt::dtt)
```

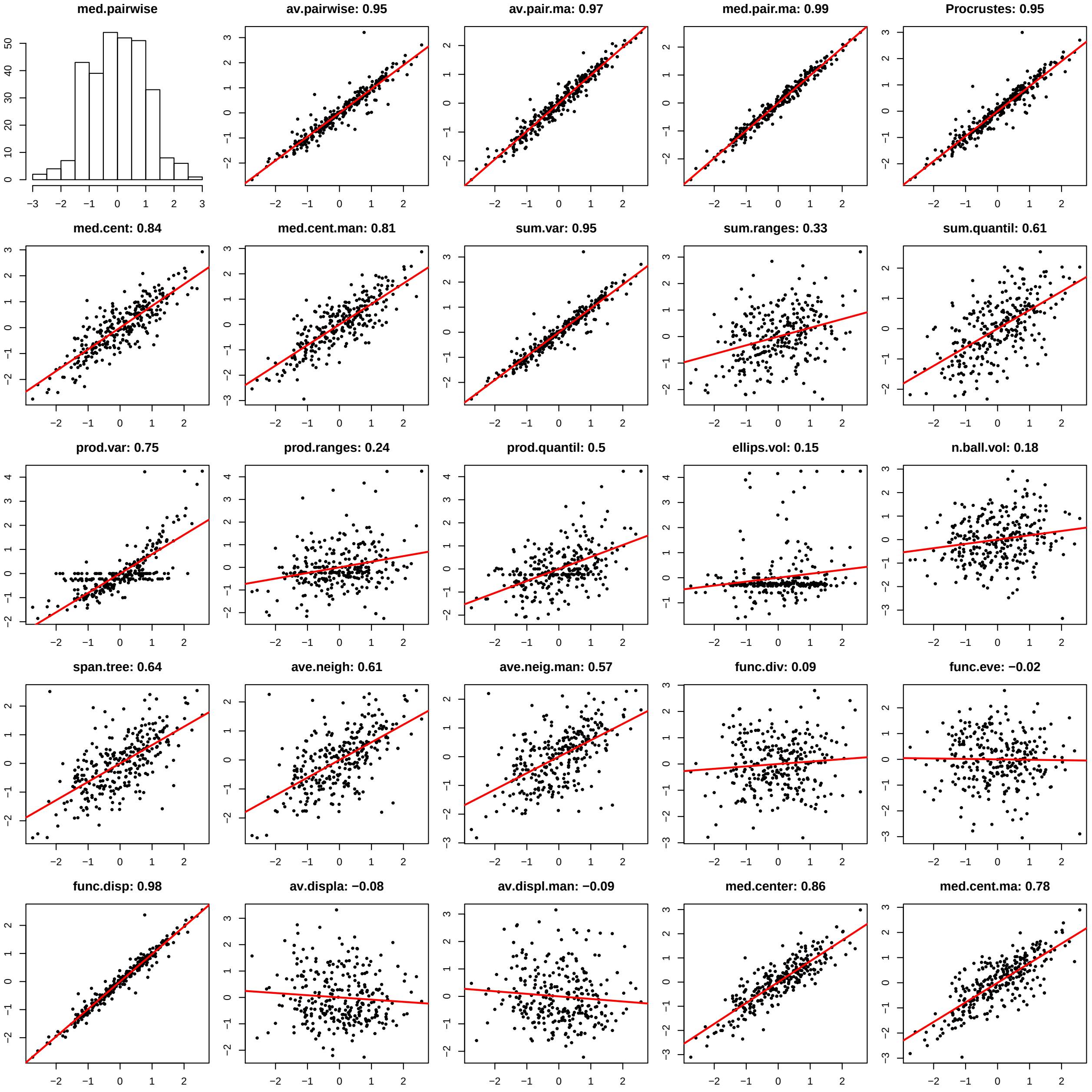

Median pairwise distance

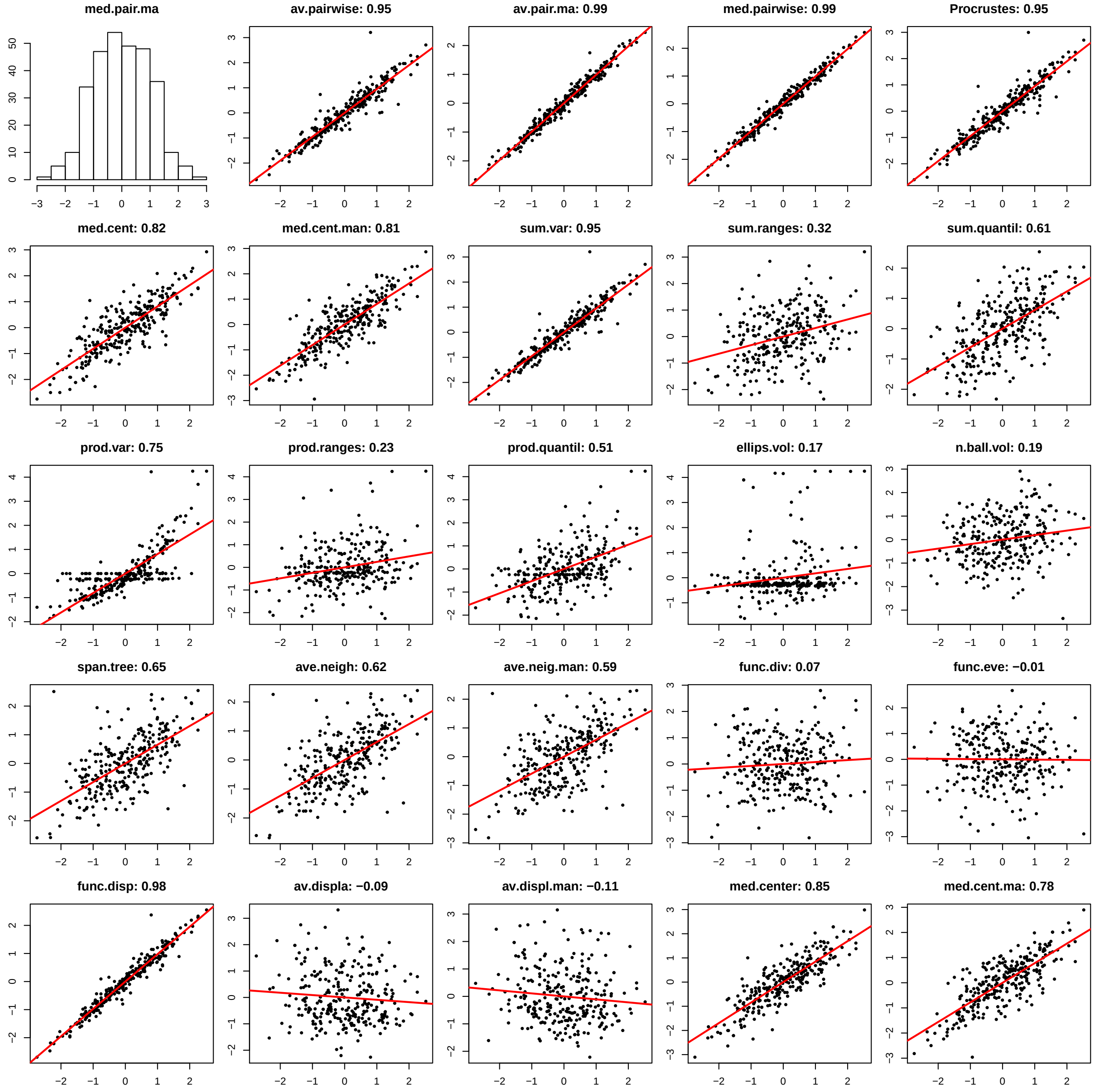


```
## Median pairwise manhattan distance
```

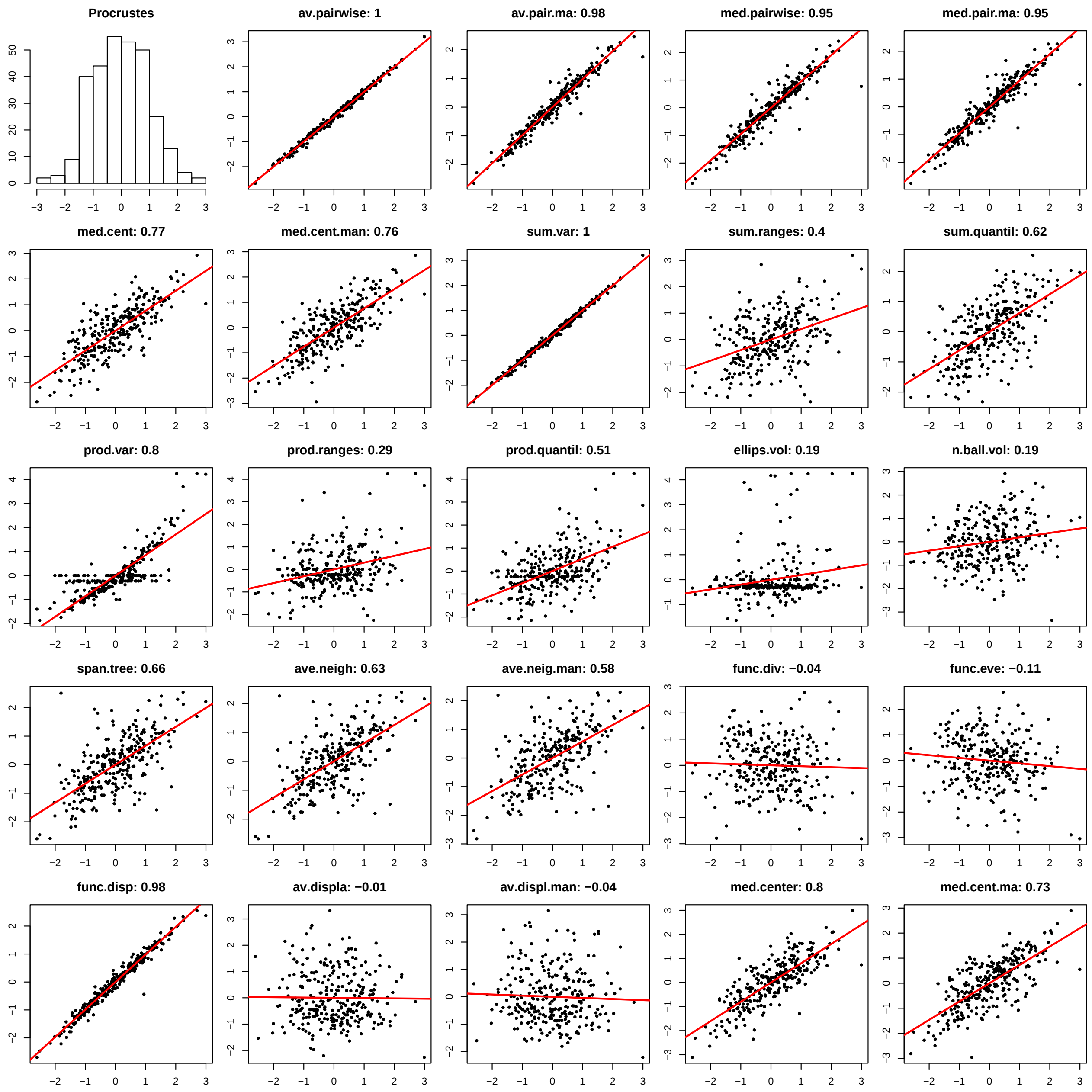


```
## Procrustes variances (geomorph::morpho.disparity)
```

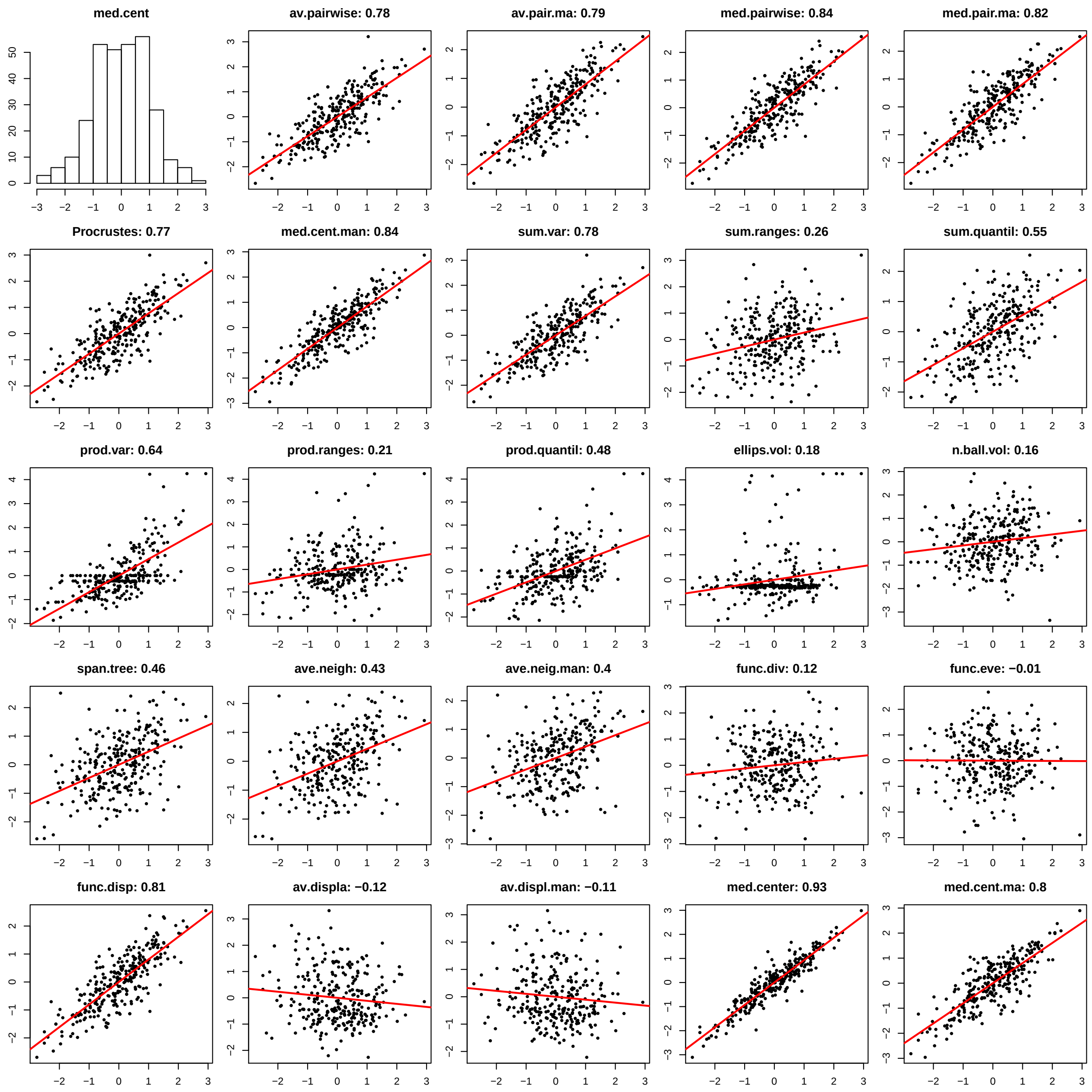


```
## Median distance from centroid
```

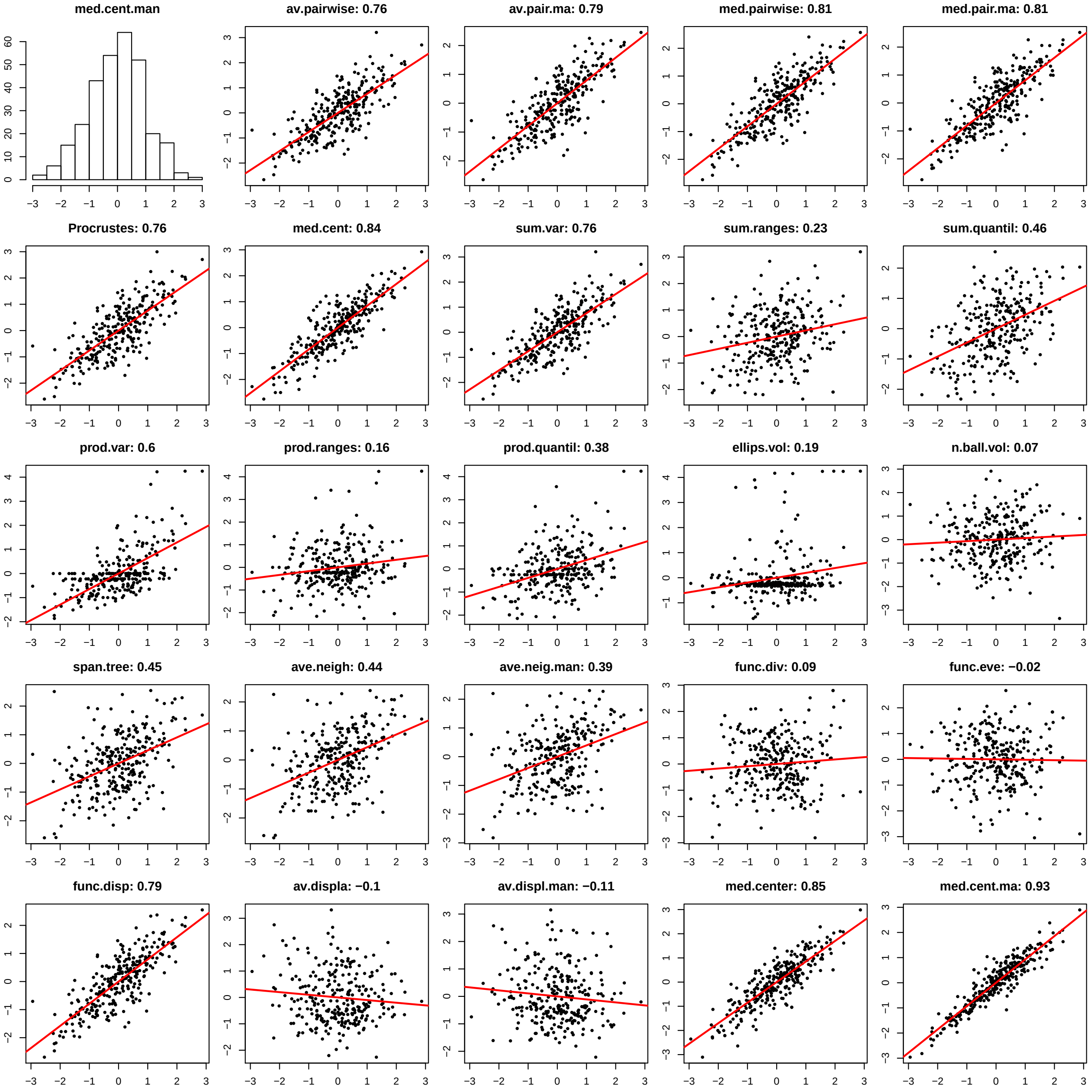

Median manhattan distance from centroid

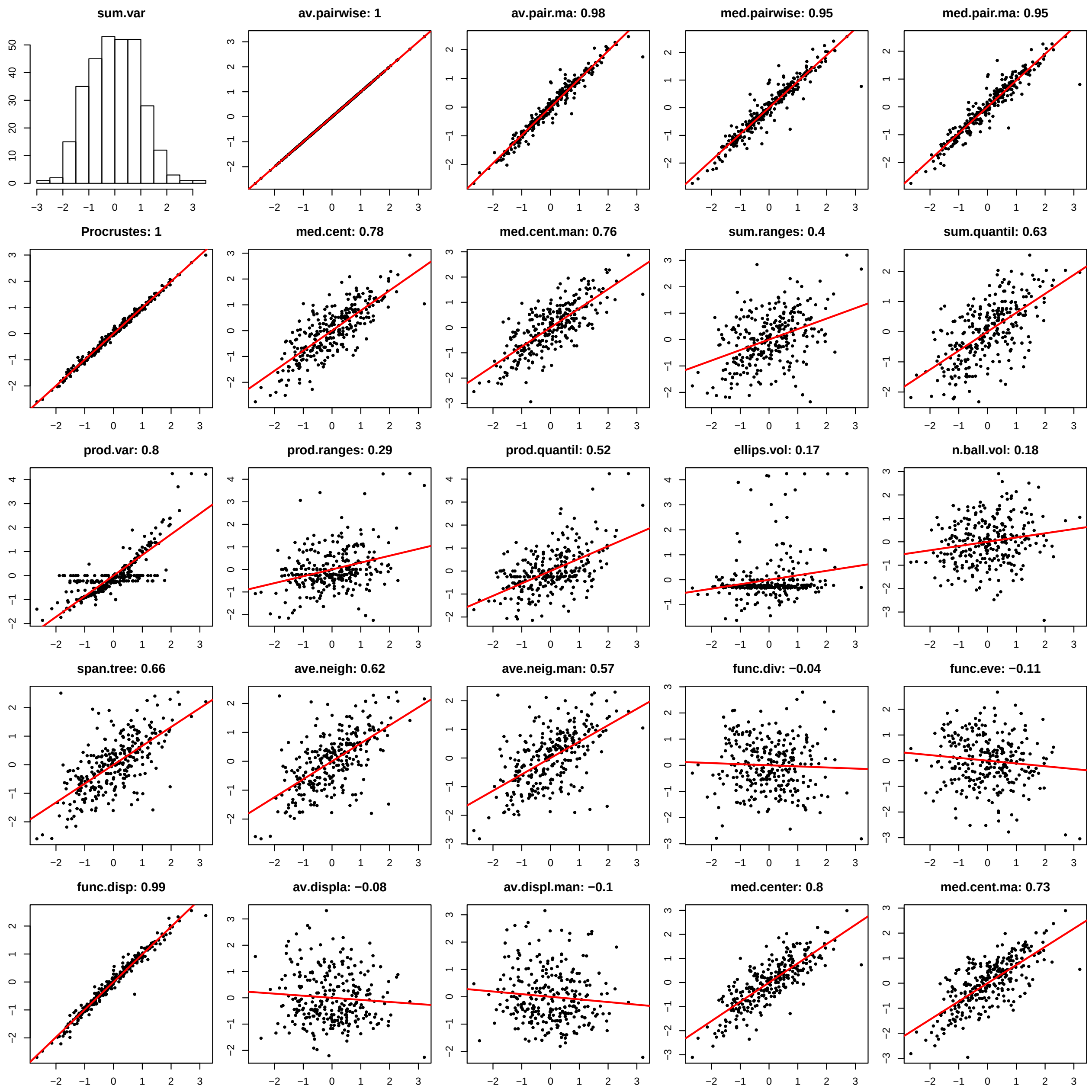


```
## Sum of variances
```

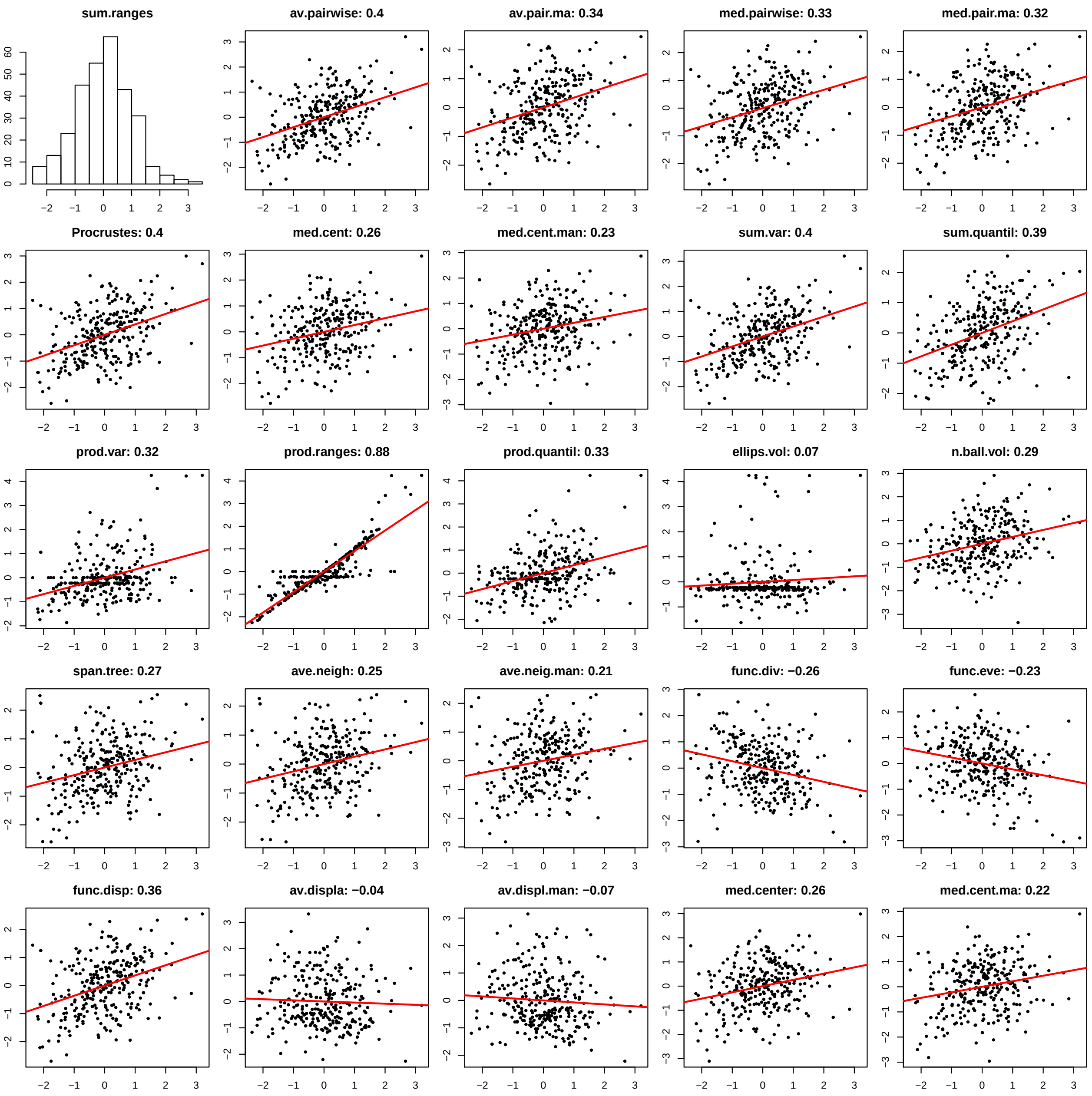

Sum of ranges

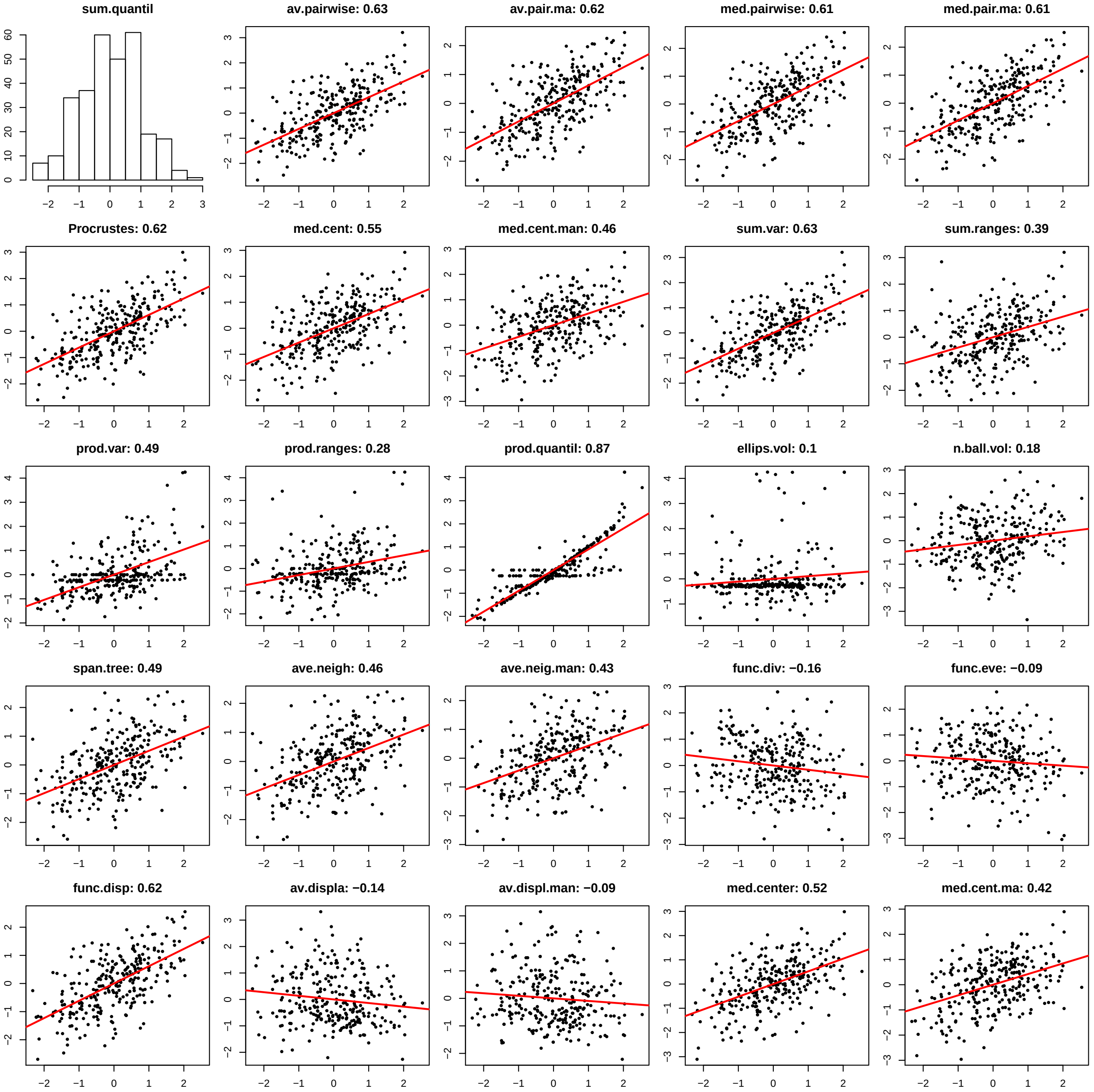


```
## Sum of quantiles
```

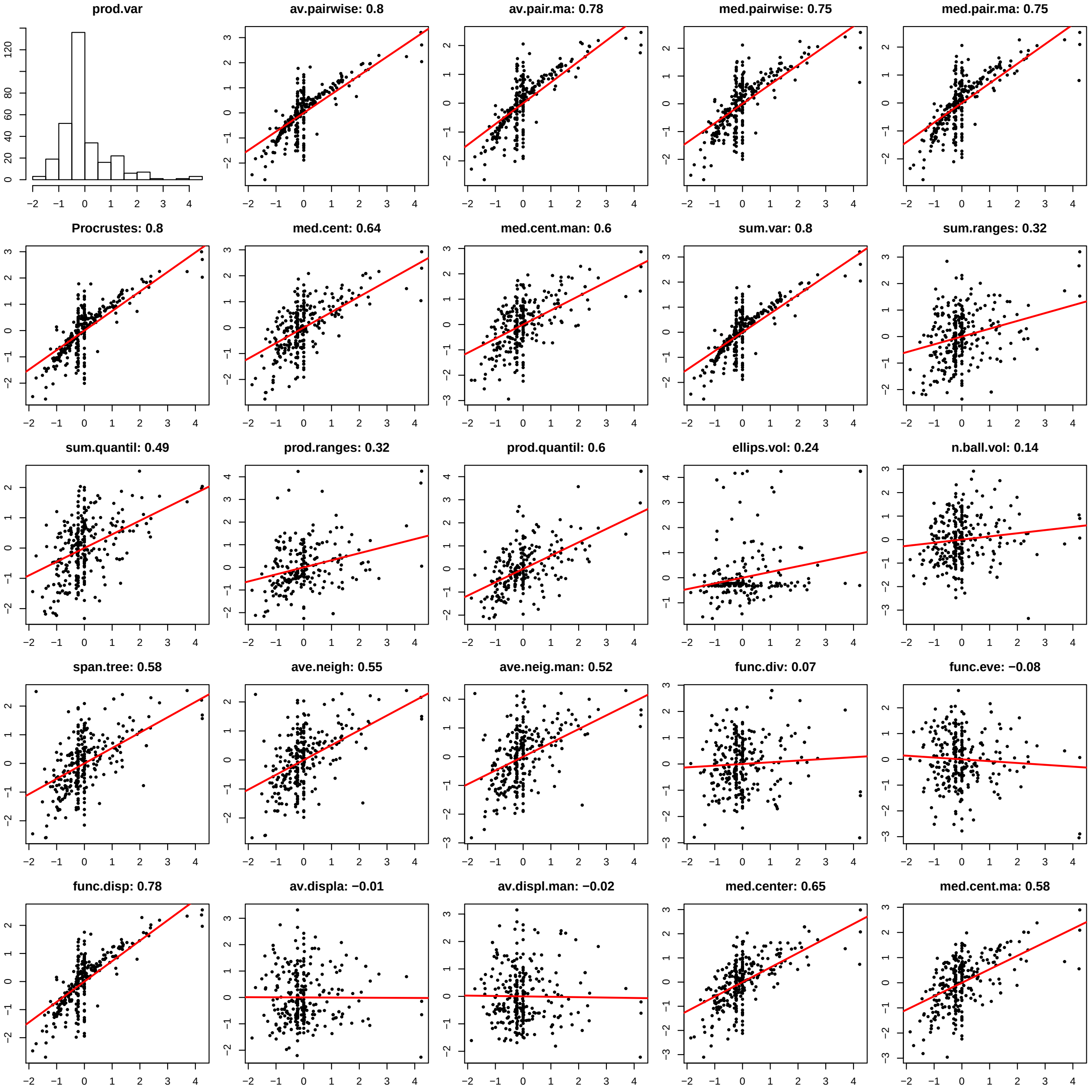


```
## Product of variances
```

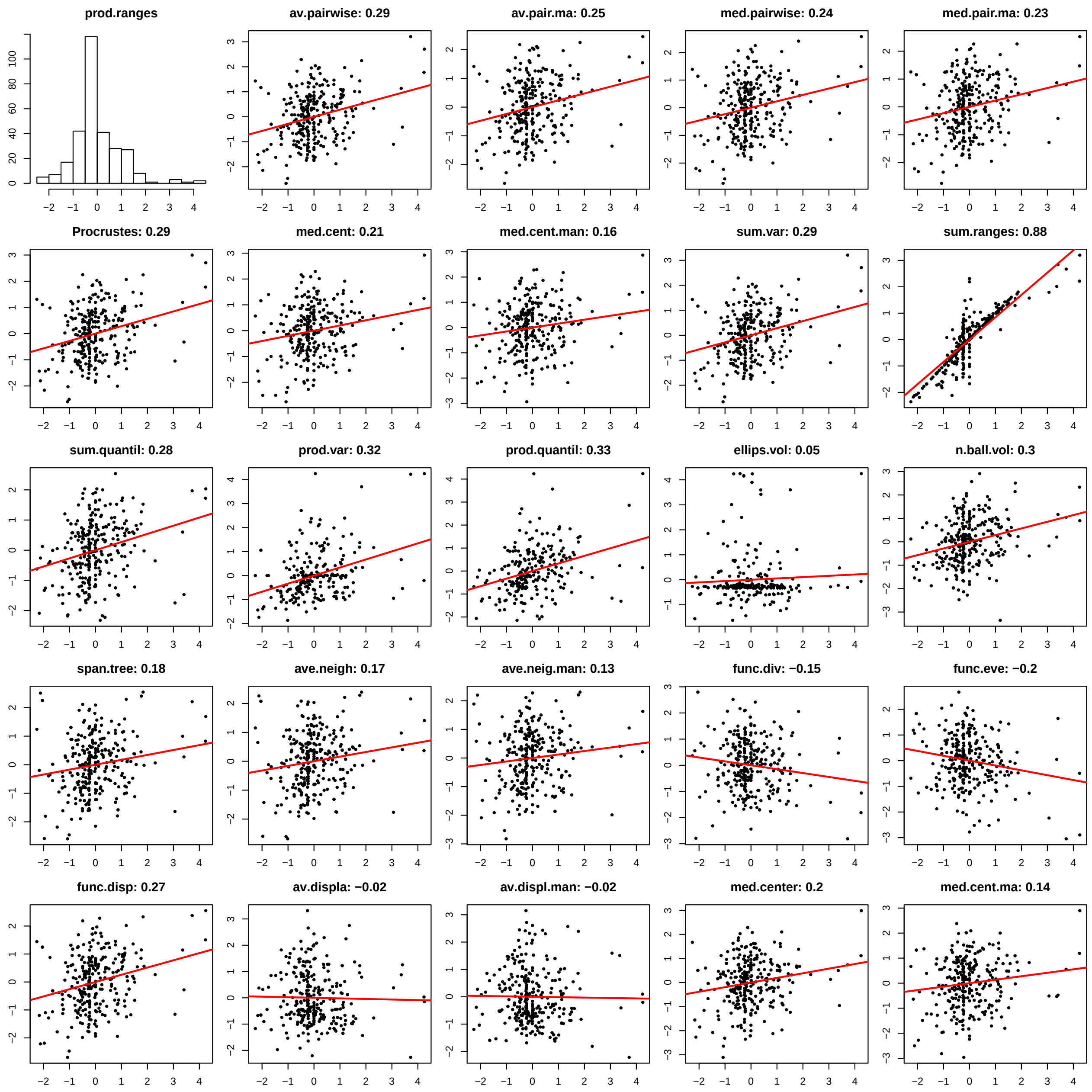


```
## Product of ranges
```

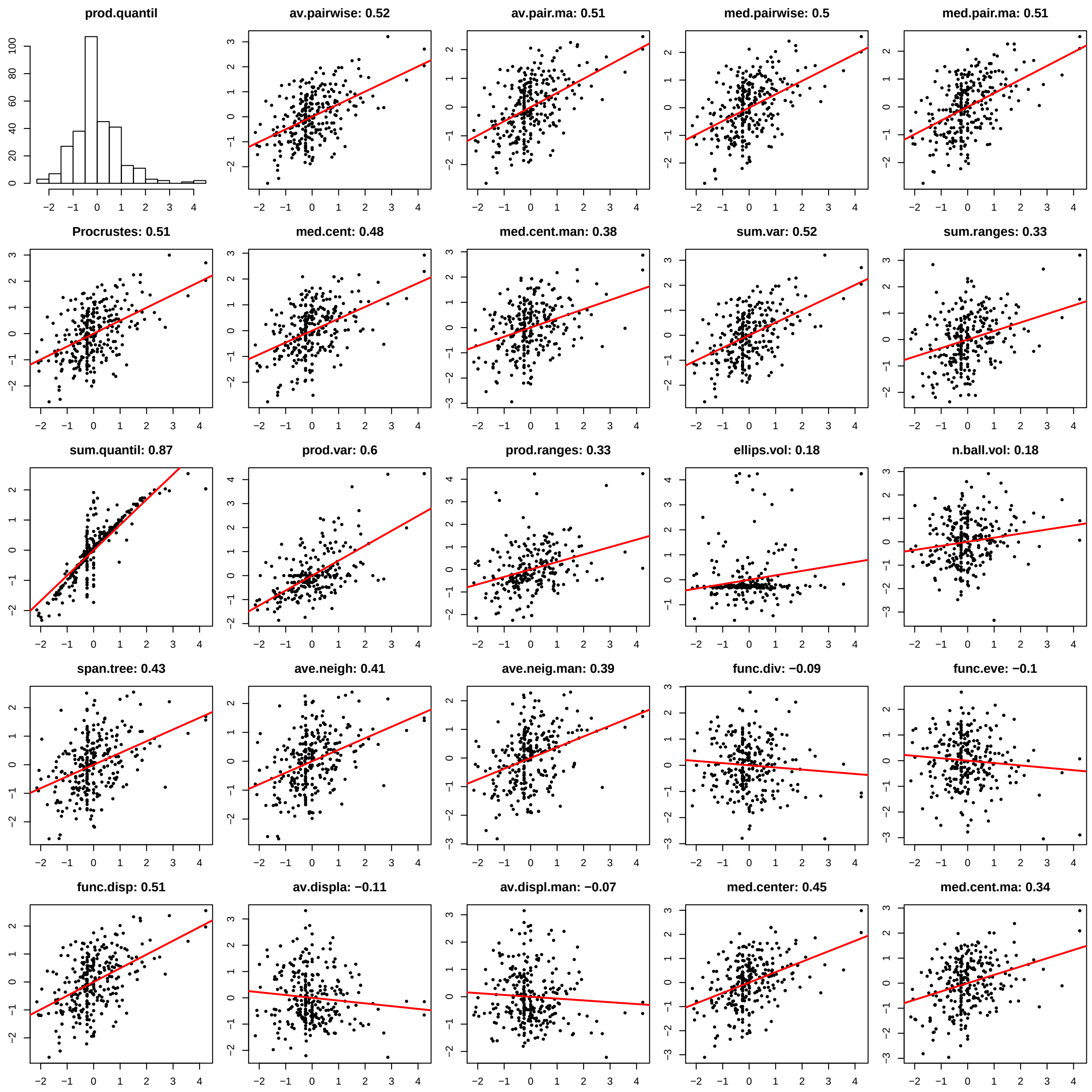


```
## Product of quantiles
```

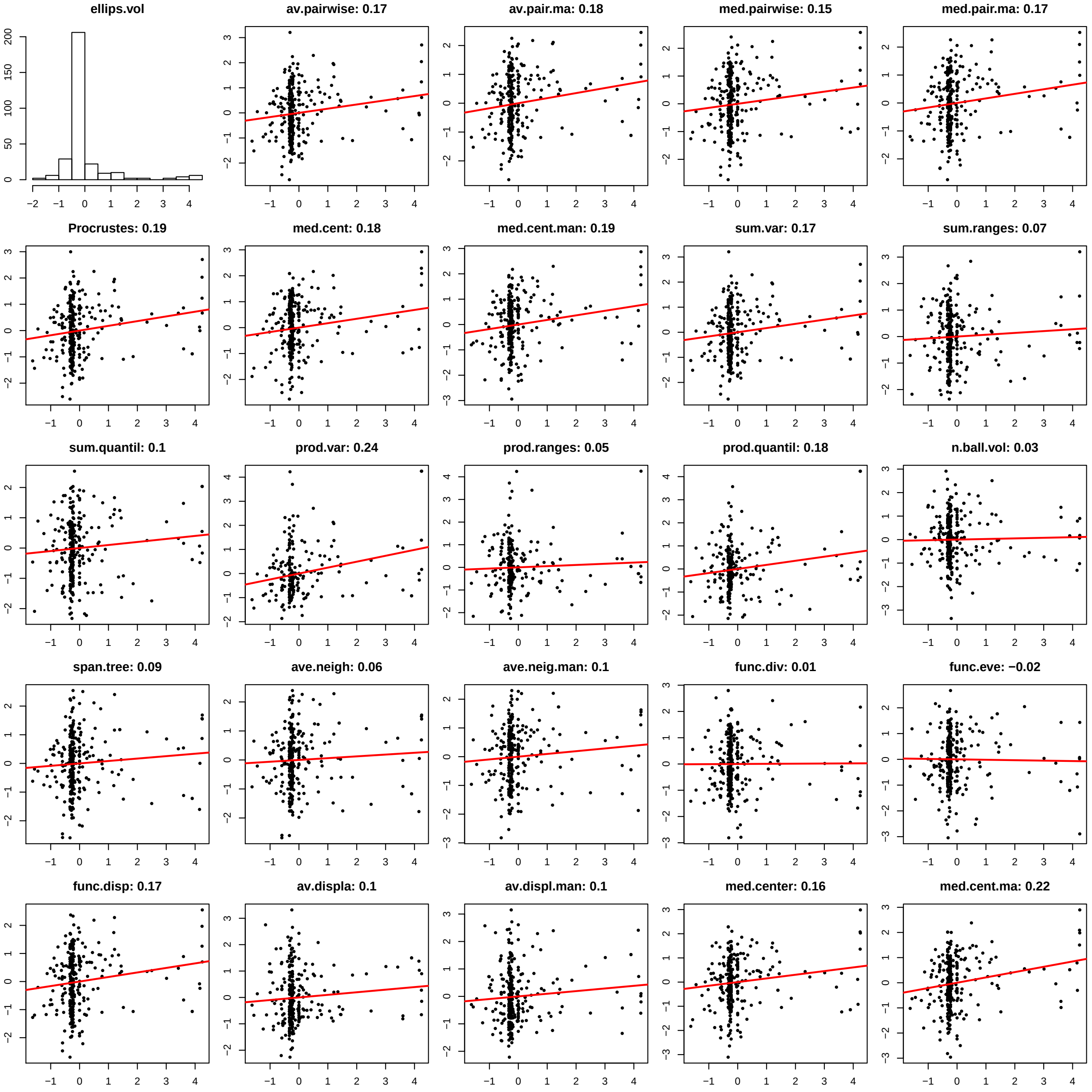


```
## Ellipsoid volume
```

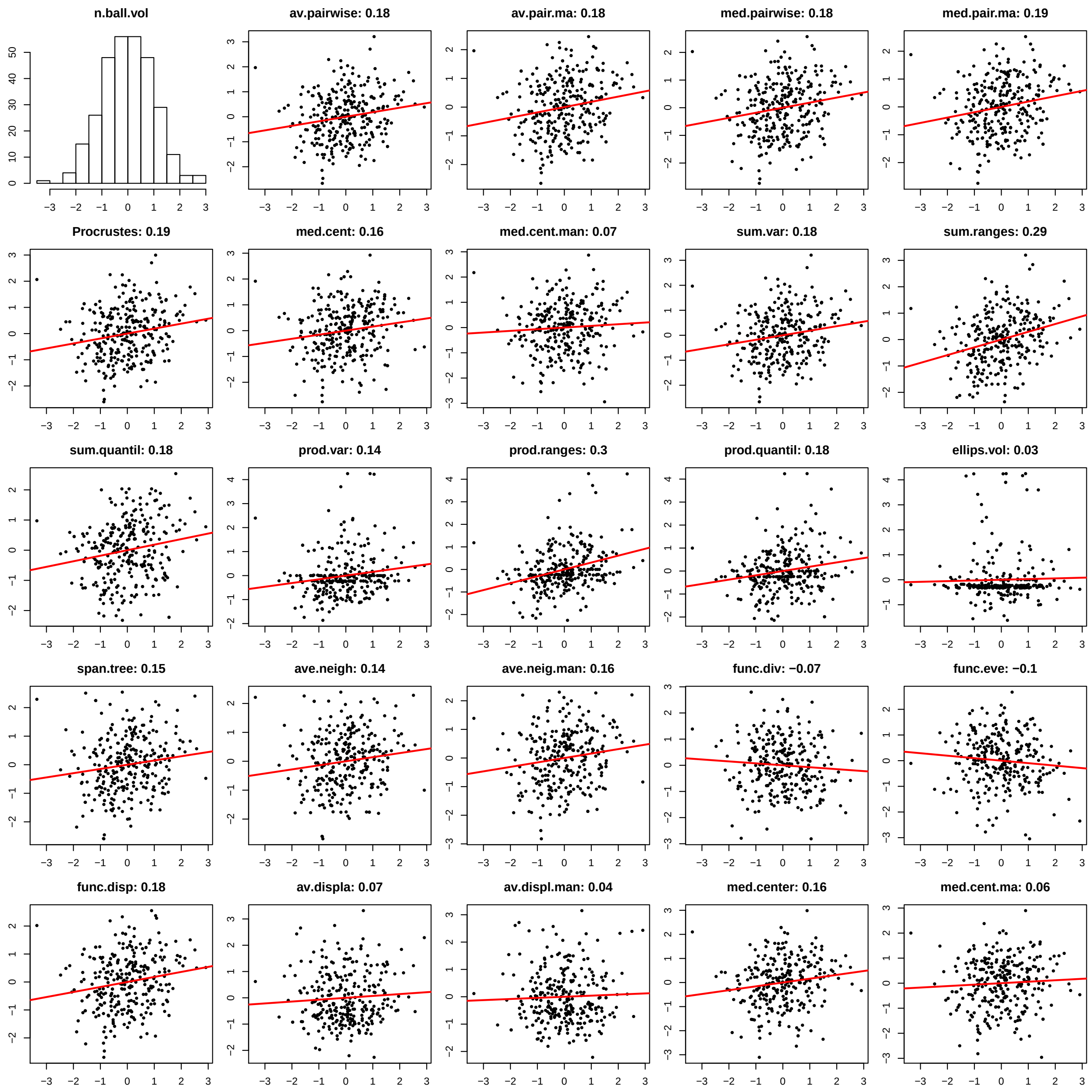


```
## nBall volume
```

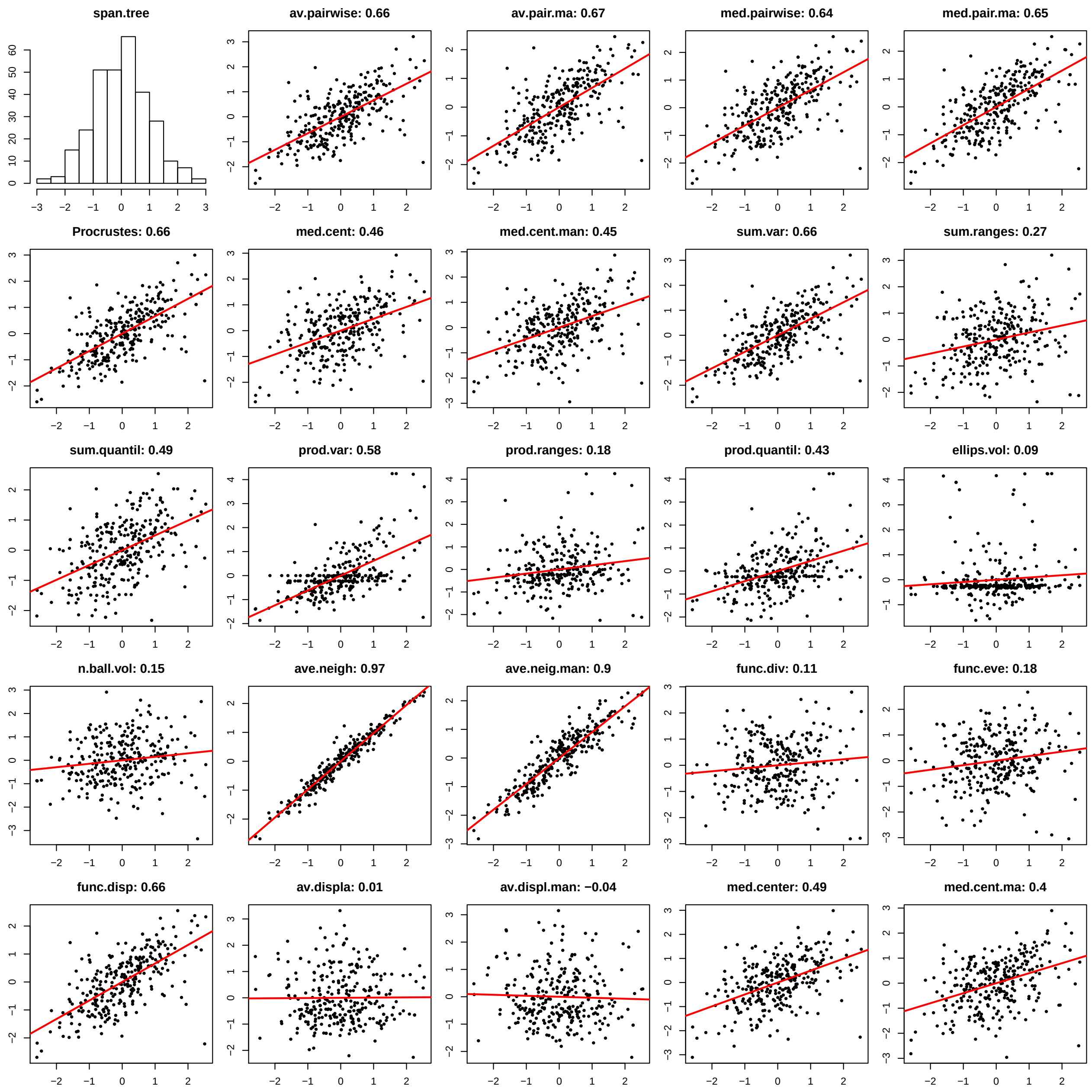


```
## Minimum spanning tree average length
```

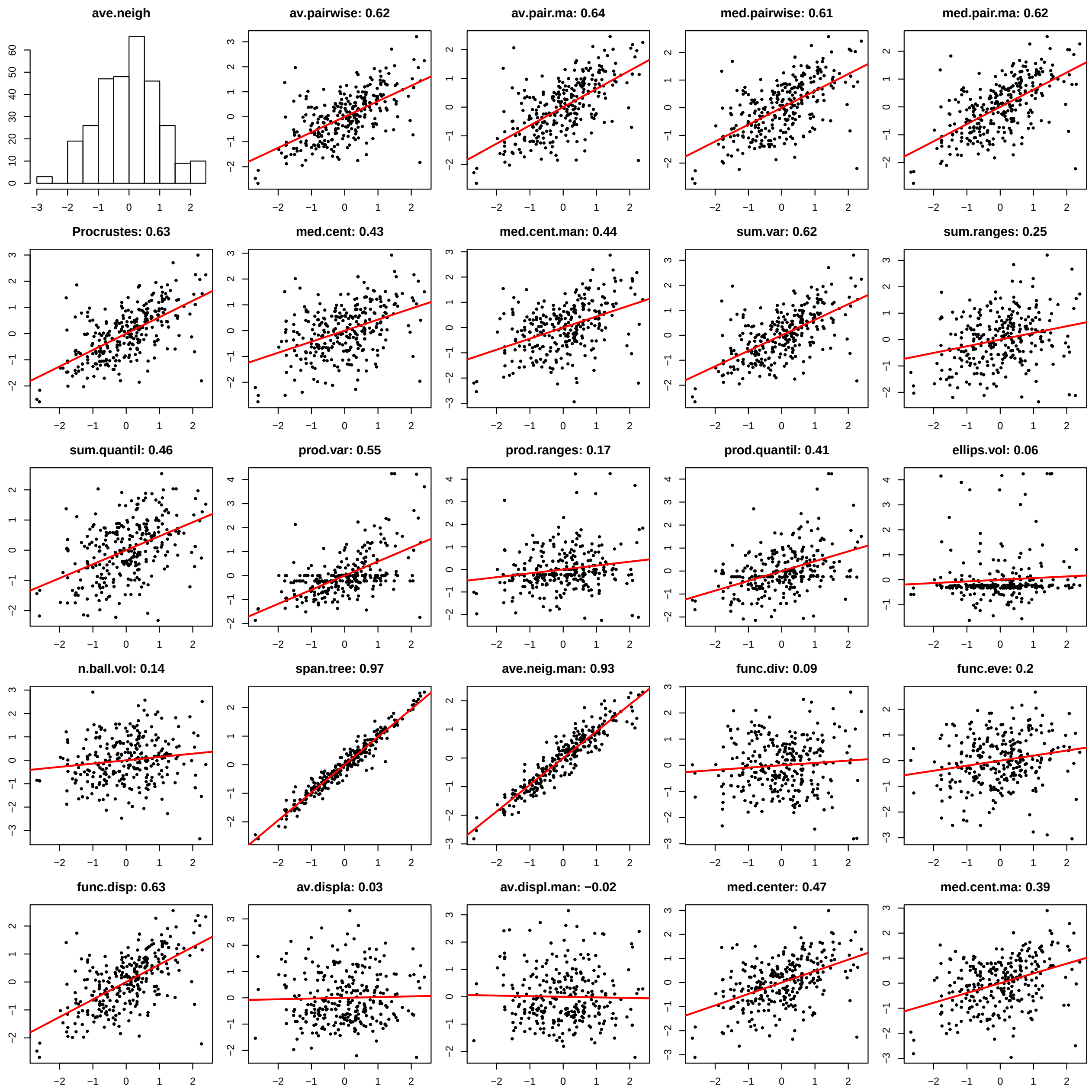


```
## Average minimum neighbours distance
```

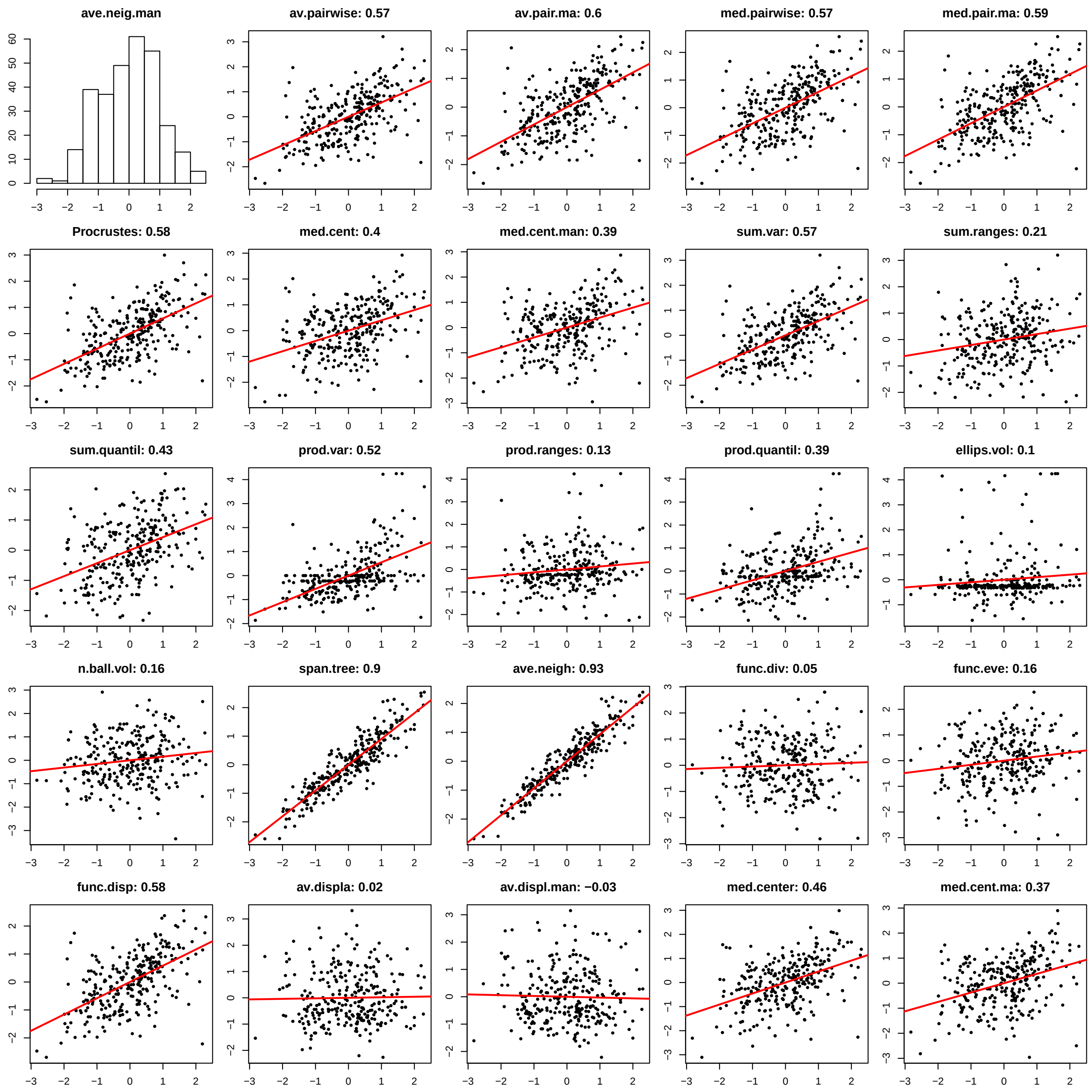


```
## Average minimum neighbours manhattan distance
```

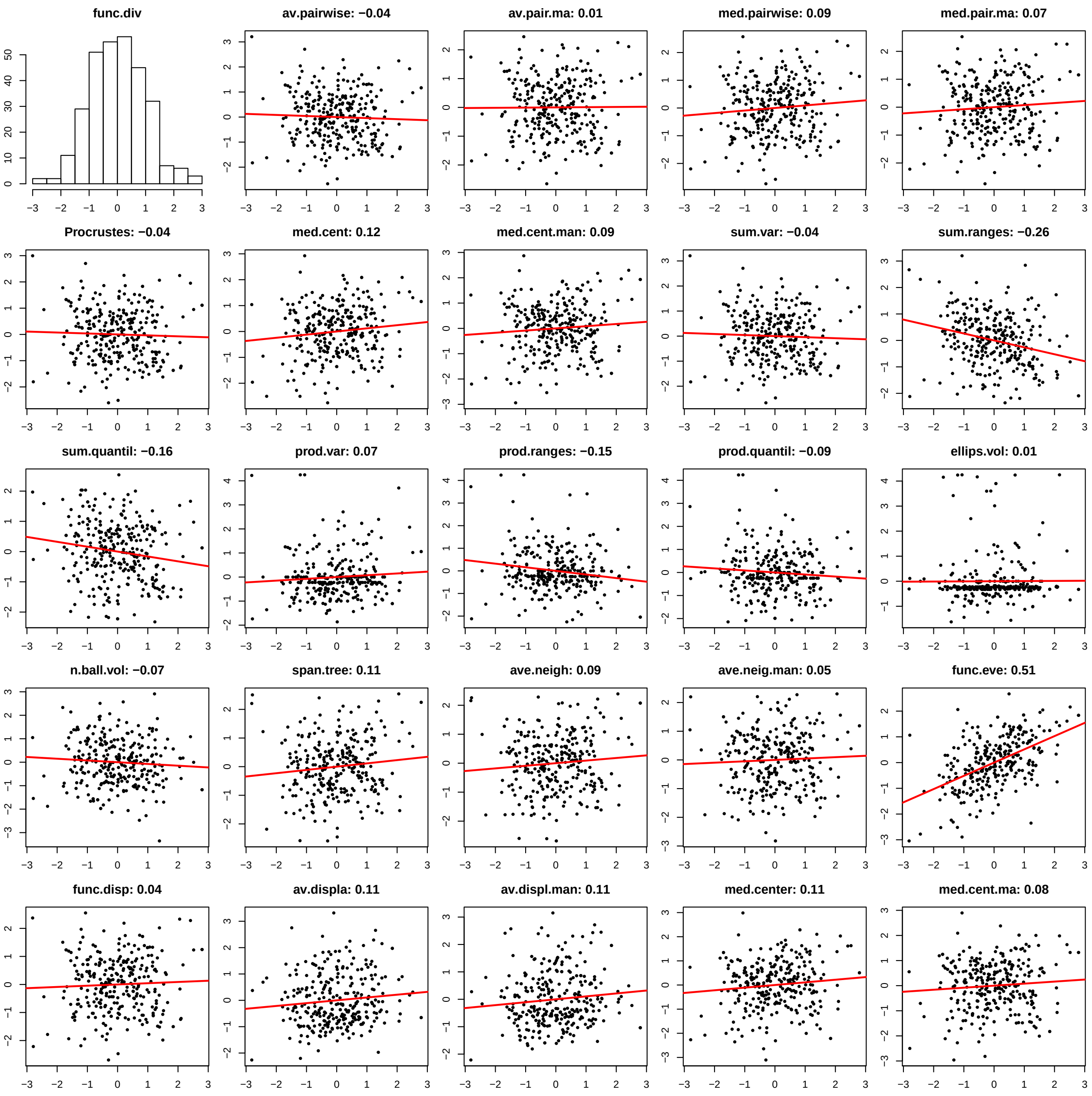


```
## Function diversity
```

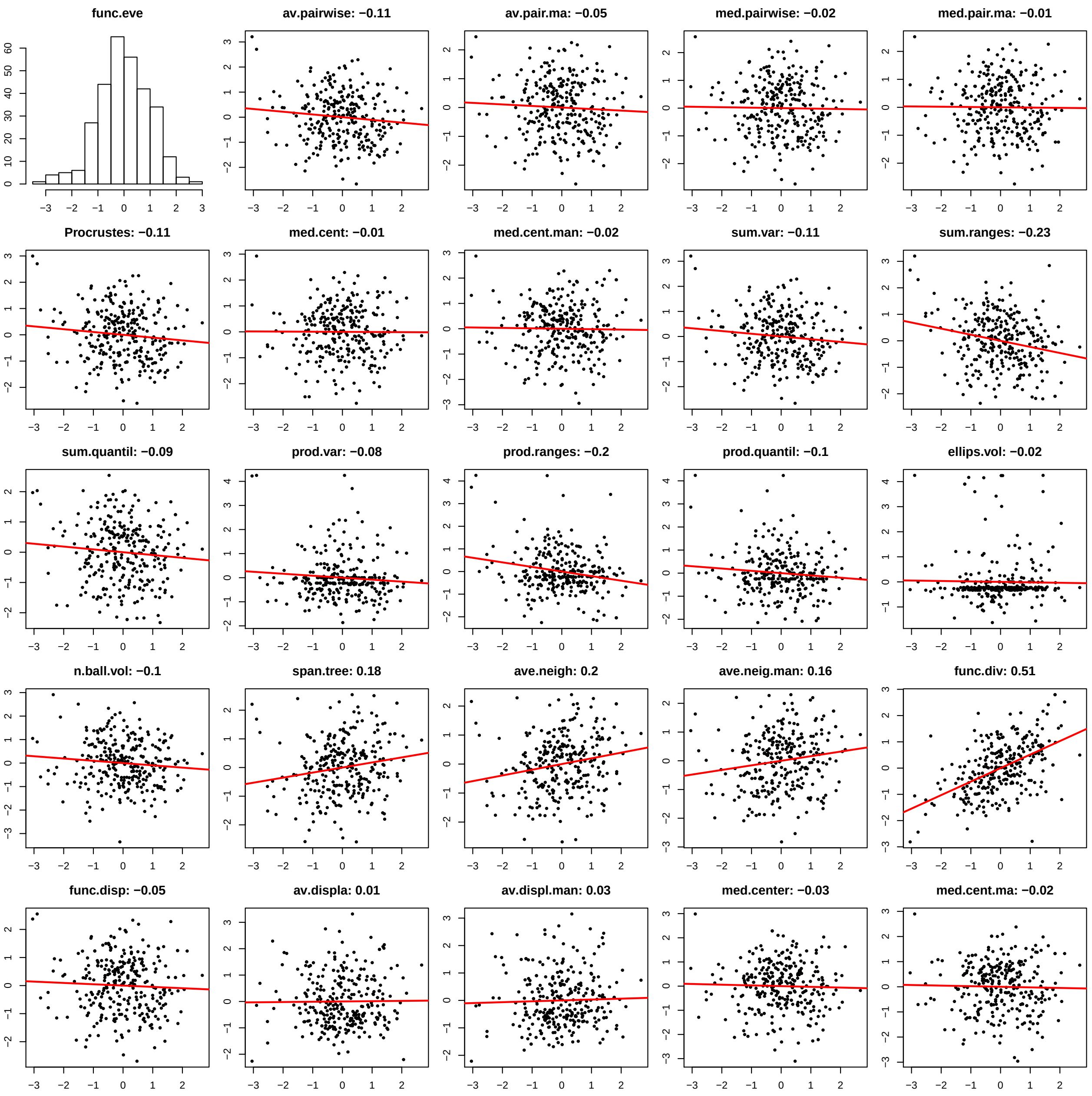


```
## Functional evenness
```

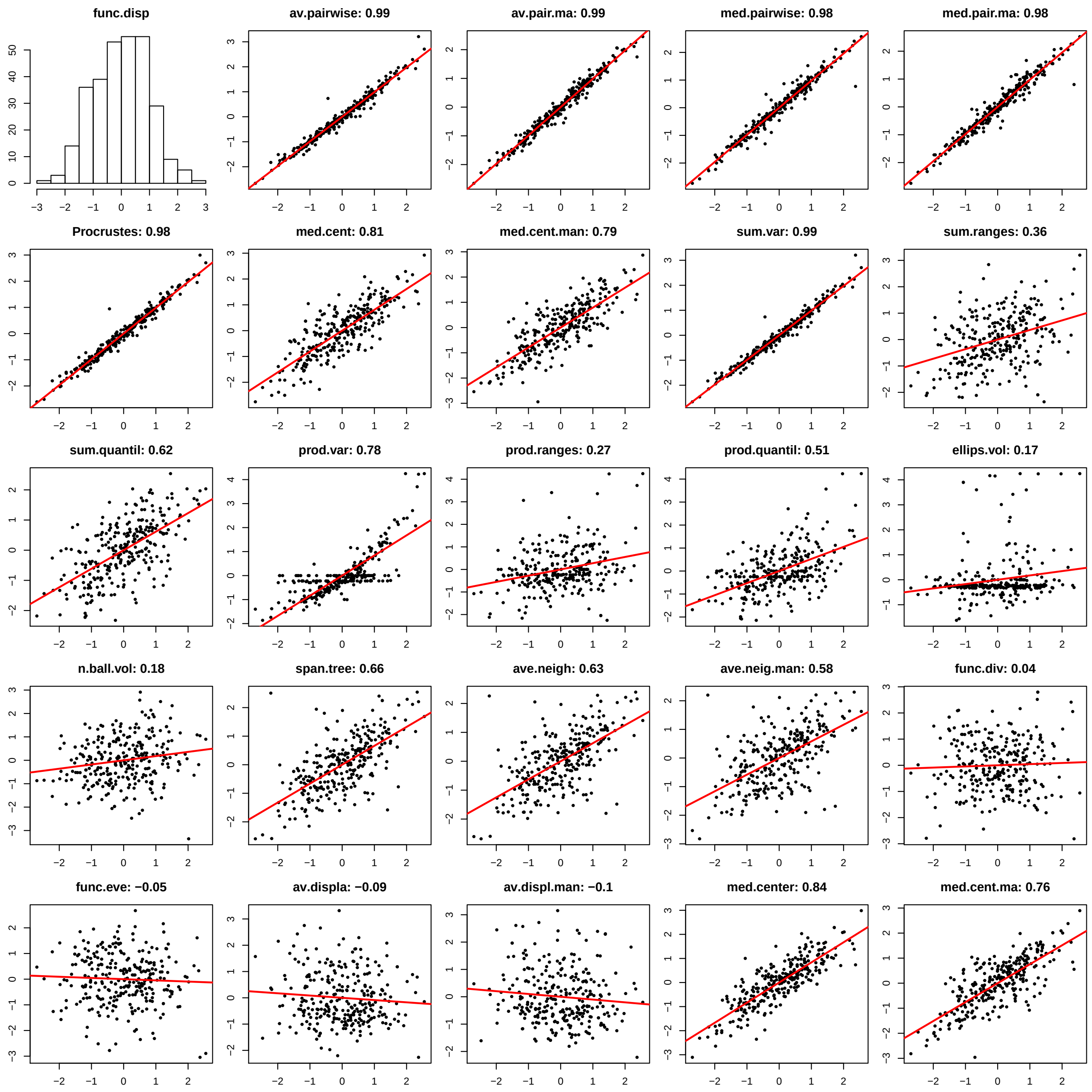


```
## Functional dispersion
```

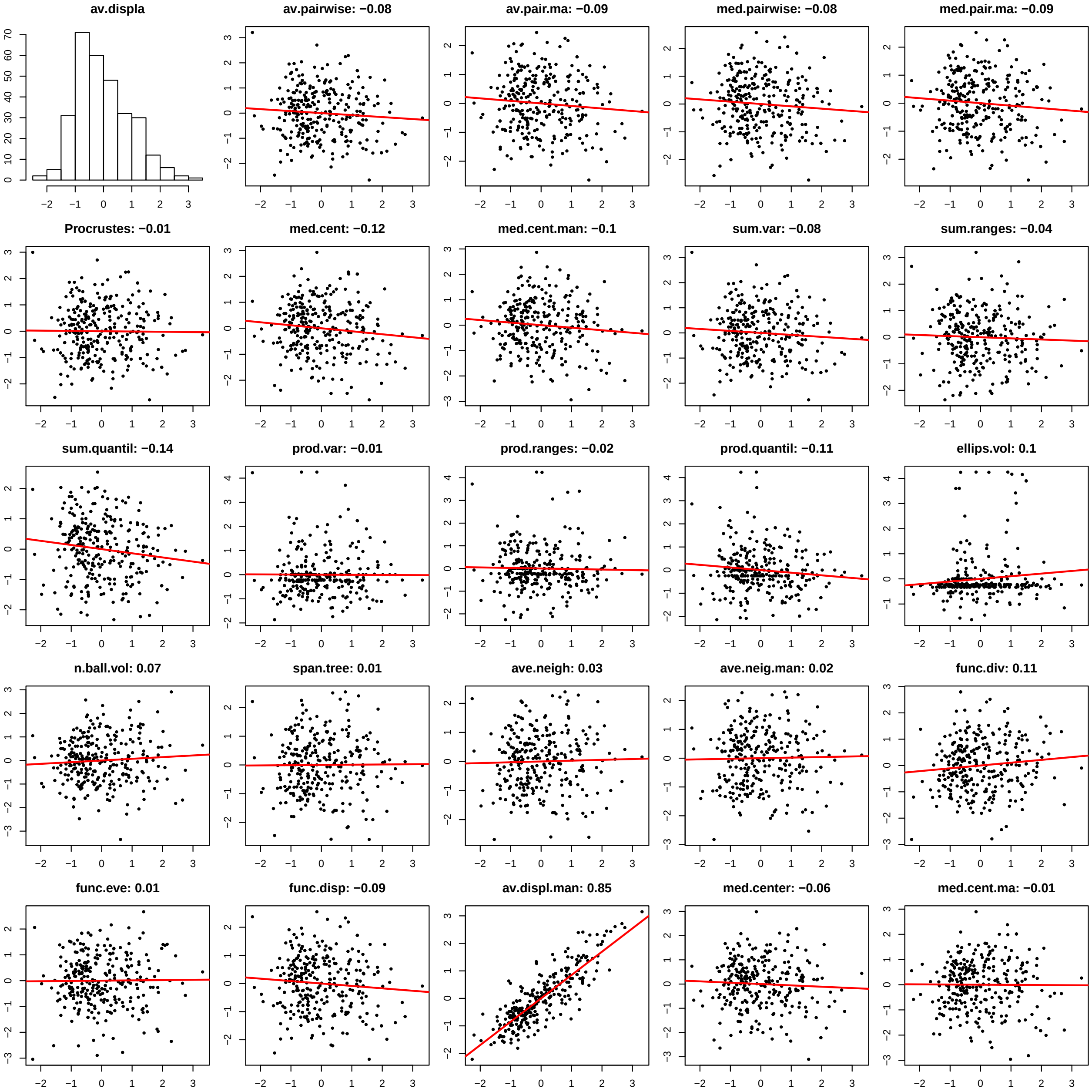

Average displacement

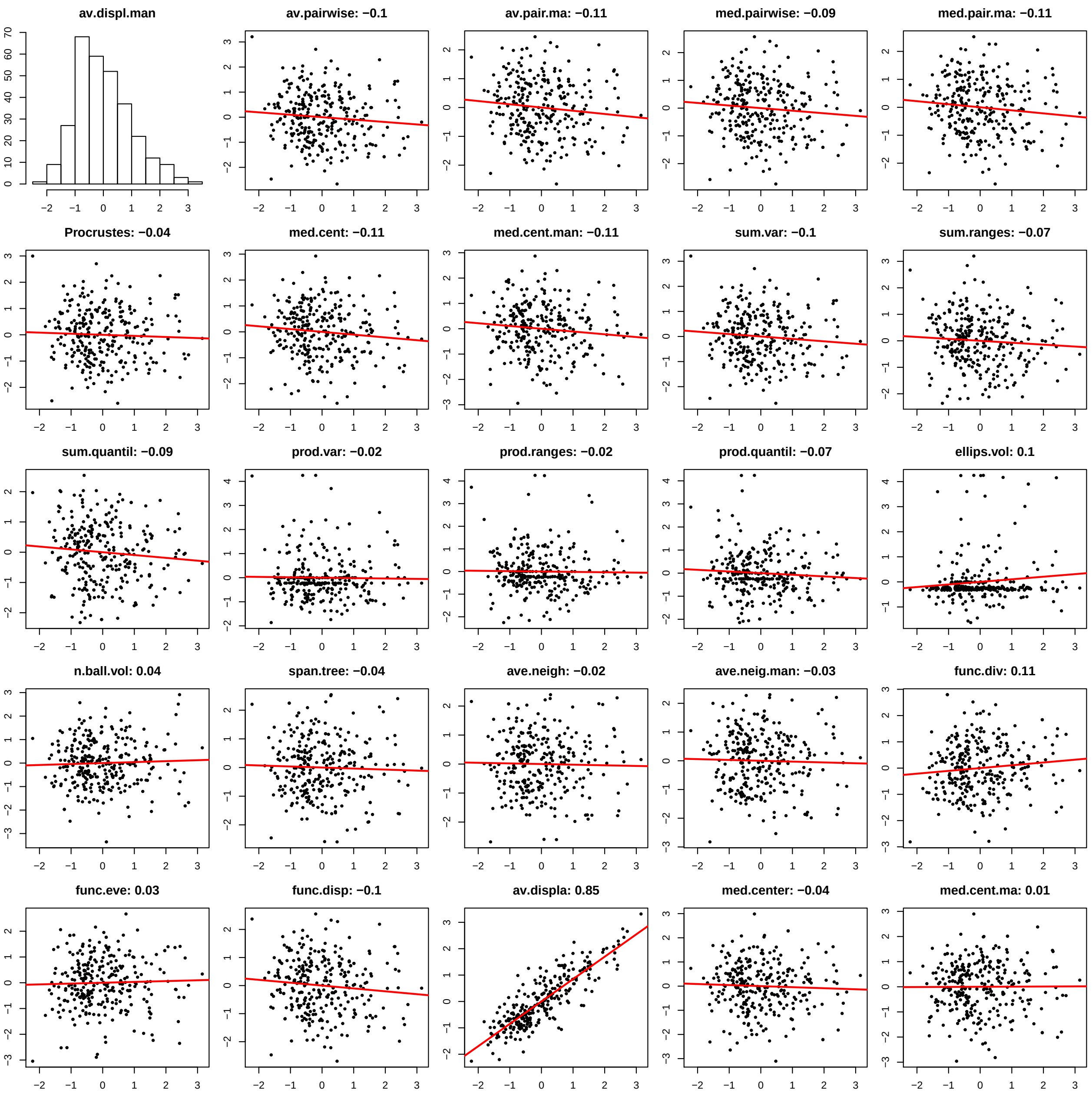


```
## Average manhattan displacement
```

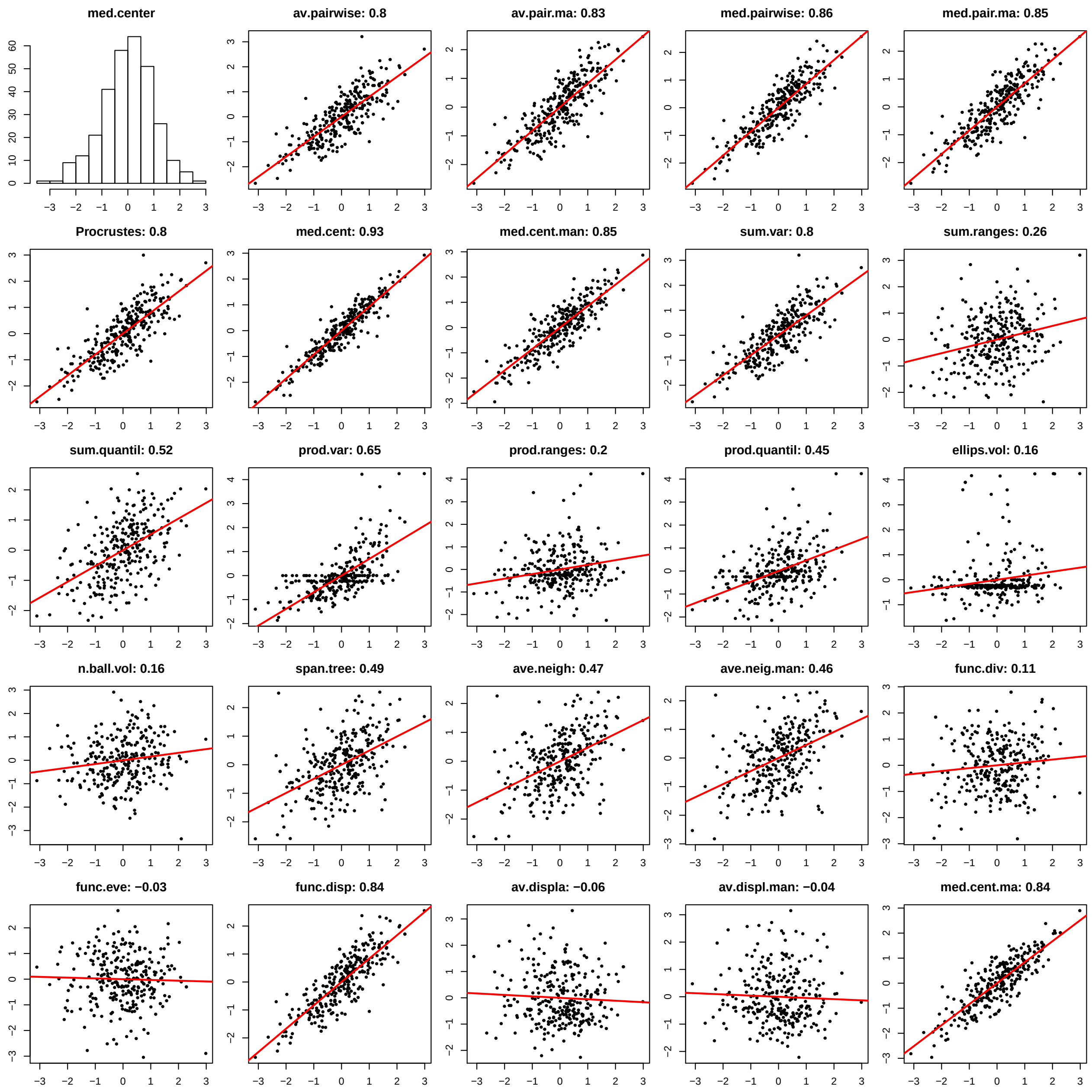


```
## Median distance from centre
```

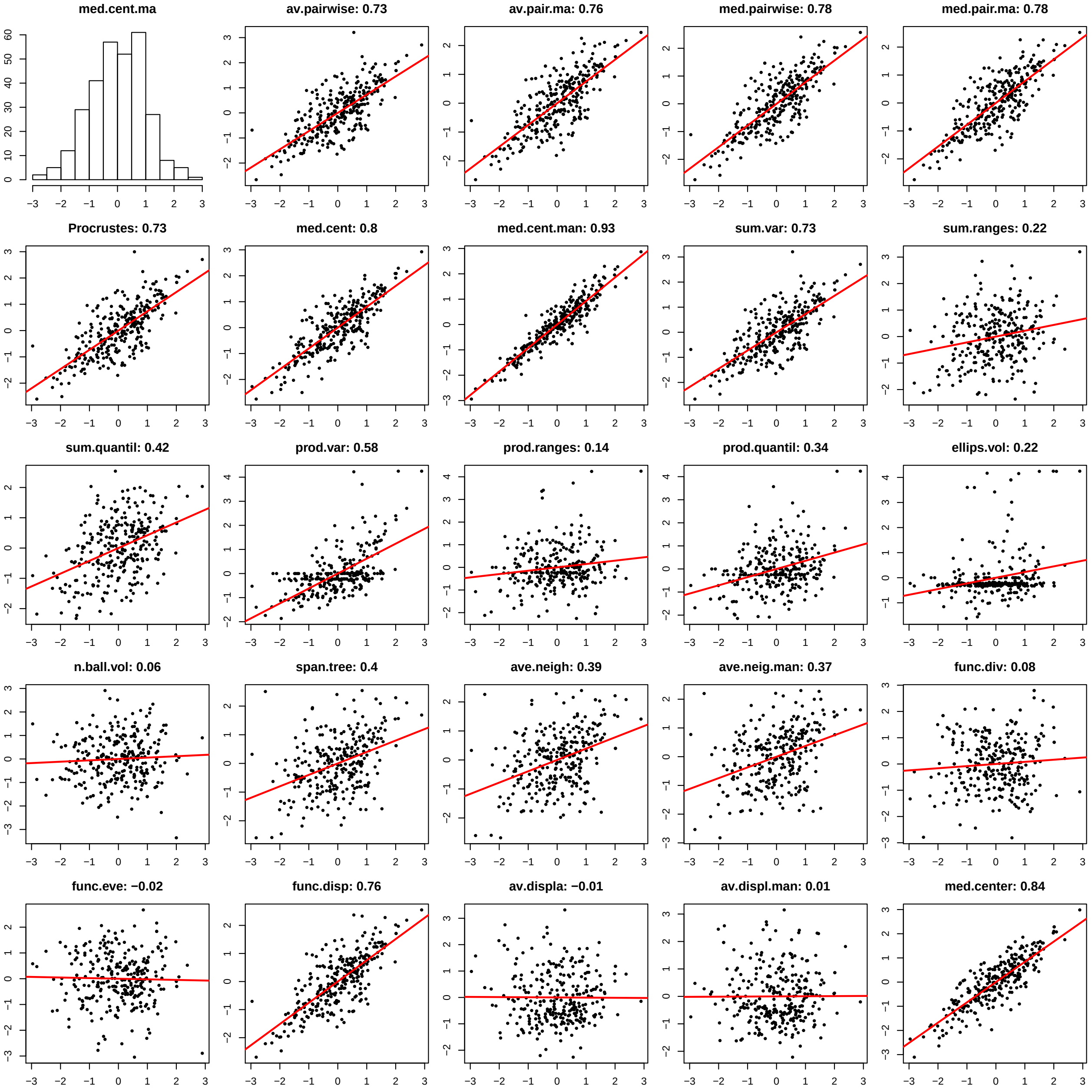


```
## Median manhattan distance from centre
```

The measurements analysed are the av.pairwise: Average squared pairwise distance (dtt::dtt); av.pair.ma: Average pairwise manhattan distance (dtt::dtt); med.pairwise: Median pairwise distance; med.pair.ma: Median pairwise manhattan distance; Procrustes: Procrustes variances (geomorph::morpho.disparity); med.cent: Median distance from centroid; med.cent.man: Median manhattan distance from centroid; sum.var: Sum of variances; sum.ranges: Sum of ranges; sum.quantil: Sum of quantiles; prod.var: Product of variances; prod.ranges: Product of ranges; prod.quantil: Product of quantiles; ellips.vol: Ellipsoid volume; n.ball.vol: nBall volume; span.tree: Minimum spanning tree average length; ave.neigh: Average minimum neighbours distance; ave.neig.man: Average minimum neighbours manhattan distance; func.div: Function diversity; func.eve: Functional evenness; func.disp: Functional dispersion; av.displa: Average displacement; av.displ.man: Average manhattan displacement; med.center: Median distance from centre; med.cent.ma: Median manhattan distance from centre.

Space shifting all measures

```
## The metrics names (shortened vector)
name <- all_metrics_names

## Making a list of parameters for each mini plot
plot.param <- list(scaler = 3,
                   bg.col = "black",
                   col = c("grey", "orange", "blue"),
                   quantiles = c(95, 50),
                   cent.tend = median,
                   pch = 19,
                   metric.max = length(all_metrics),
                   cex = 2)
```

Table 1: All measures results 20% removal

Metric	Size change	Density change	Position change	Distribution effect	Dimensions effect
Average squared pairwise distance (dtt::dtt)	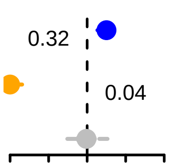	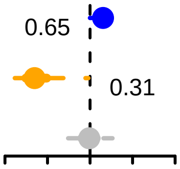	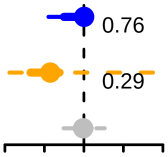	F = 7.302 ; p = <1e-3***	F = 0.169 ; p = 0.995
Average pairwise manhattan distance (dtt::dtt)	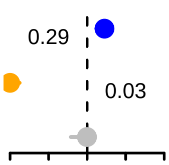	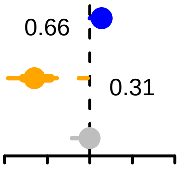	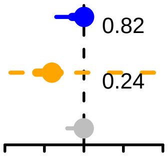	F = 3.133 ; p = 0.014*	F = 0.103 ; p = 0.999
Median pairwise distance	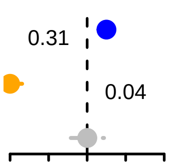	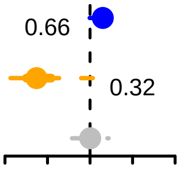	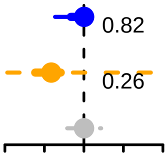	F = 5.163 ; p = <1e-3***	F = 0.09 ; p = 0.999

Metric	Size change	Density change	Position change	Distribution effect	Dimensions effect
Median pairwise manhattan distance	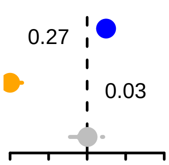	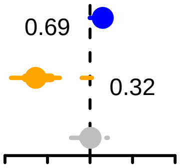	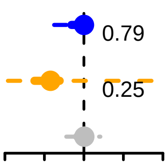	F = 4.043 ; p = 0.003**	F = 0.085 ; p = 1
Procrustes variances (geomorph::morpho.disparity)	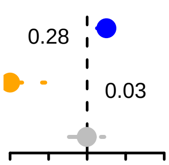	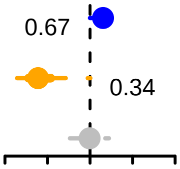	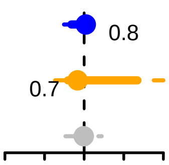	F = 5.255 ; p = <1e-3***	F = 0.764 ; p = 0.634
Median distance from centroid	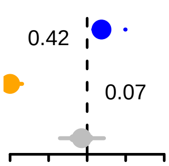	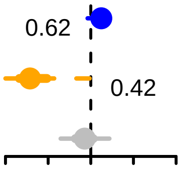	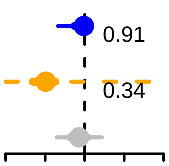	F = 7.995 ; p = <1e-3***	F = 0.219 ; p = 0.988
Median manhattan distance from centroid	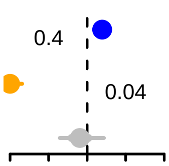	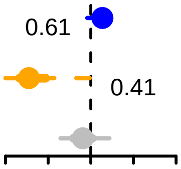	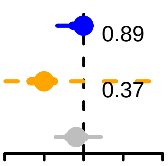	F = 4.726 ; p = 0.001**	F = 0.306 ; p = 0.964
Sum of variances	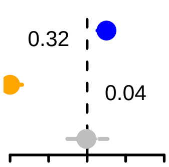	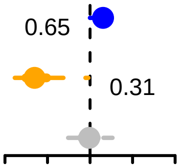	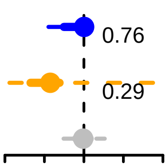	F = 7.302 ; p = <1e-3***	F = 0.169 ; p = 0.995
Sum of ranges	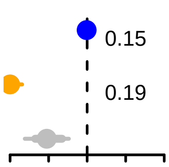	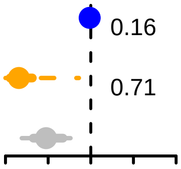	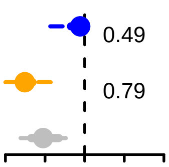	F = 0.778 ; p = 0.539	F = 4.162 ; p = <1e-3***

Metric	Size change	Density change	Position change	Distribution effect	Dimensions effect
Sum of quantiles	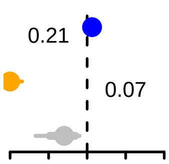	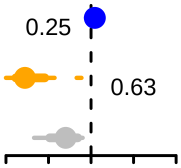	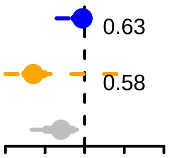	F = 3.643 ; p = 0.006**	F = 3.076 ; p = 0.002**
Product of variances	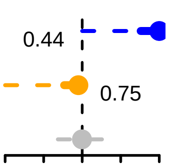	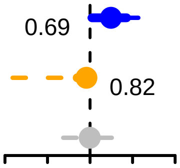	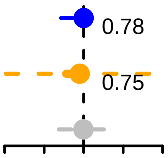	F = 7.564 ; p = <1e-3***	F = 22.082 ; p = <1e-3***
Product of ranges	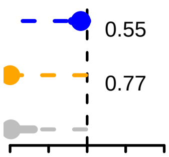	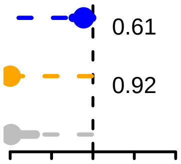	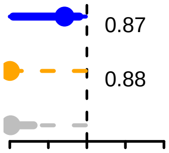	F = 155.586 ; p = <1e-3***	F = 30.835 ; p = <1e-3***
Product of quantiles	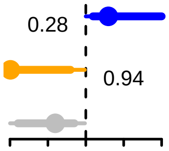	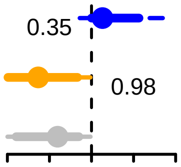	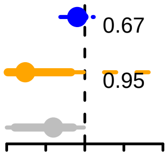	F = 39.186 ; p = <1e-3***	F = 27.112 ; p = <1e-3***
Ellipsoid volume	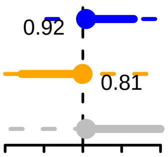	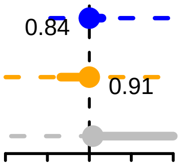	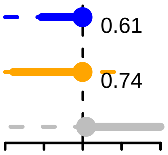	F = 6.354 ; p = <1e-3***	F = 30.018 ; p = <1e-3***
nBall volume	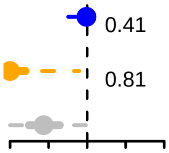	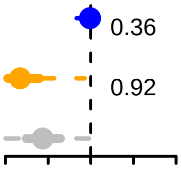	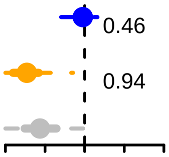	F = 2.349 ; p = 0.053.	F = 5.266 ; p = <1e-3***
Minimum spanning tree average length	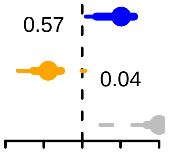	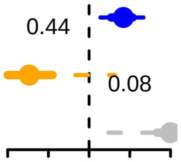	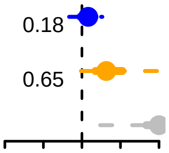	F = 0.971 ; p = 0.422	F = 1.335 ; p = 0.222

<table>
<thead>
<tr><th>Metric</th><th>Size change</th><th>Density change</th><th>Position change</th><th>Distribution effect</th><th>Dimensions effect</th></tr>
</thead>
<tbody>
<tr><td>Average minimum neighbours distance</td><td>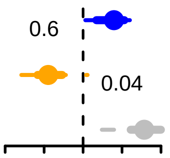
</td><td>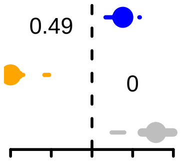
</td><td>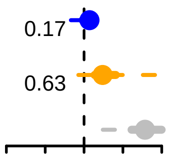
</td><td>$F = 0.21$; $p = 0.933$</td><td>$F = 0.699$; $p = 0.692$</td></tr>
<tr><td>Average minimum neighbours manhattan distance</td><td>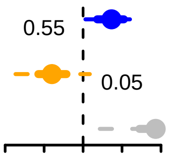
</td><td>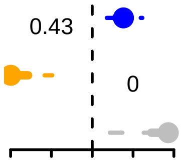
</td><td>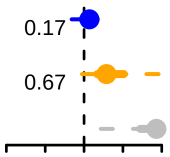
</td><td>$F = 0.854$; $p = 0.491$</td><td>$F = 1.386$; $p = 0.198$</td></tr>
<tr><td>Function diversity</td><td>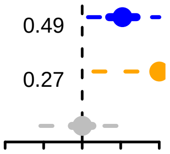
</td><td>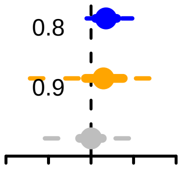
</td><td>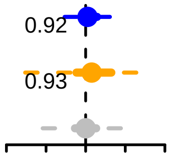
</td><td>$F = 1.368$; $p = 0.243$</td><td>$F = 4.113$; $p = <1e-3$***</td></tr>
<tr><td>Functional evenness</td><td>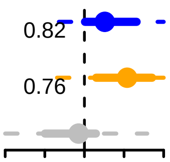
</td><td>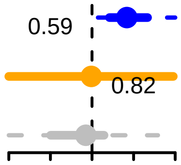
</td><td>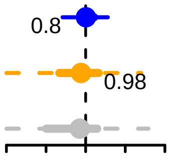
</td><td>$F = 36.357$; $p = <1e-3$***</td><td>$F = 32.573$; $p = <1e-3$***</td></tr>
<tr><td>Functional dispersion</td><td>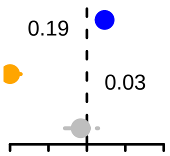
</td><td>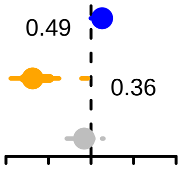
</td><td>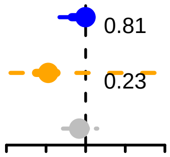
</td><td>$F = 3.911$; $p = 0.004$**</td><td>$F = 0.116$; $p = 0.999$</td></tr>
<tr><td>Average displacement</td><td>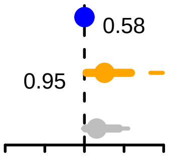
</td><td>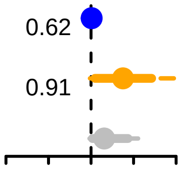
</td><td>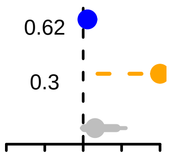
</td><td>$F = 5.809$; $p = <1e-3$***</td><td>$F = 14.553$; $p = <1e-3$***</td></tr>
</tbody>
</table>

Metric	Size change	Density change	Position change	Distribution effect	Dimensions effect
Average manhattan displacement	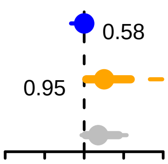	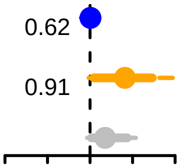	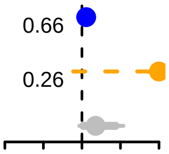	F = 5.381 ; p = <1e-3***	F = 13.378 ; p = <1e-3***
Median distance from centre	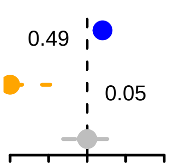	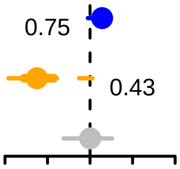	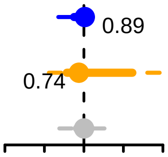	F = 6.027 ; p = <1e-3***	F = 0.918 ; p = 0.501
Median manhattan distance from centre	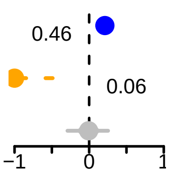	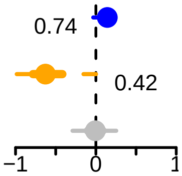	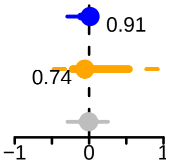	F = 5.467 ; p = <1e-3***	F = 0.978 ; p = 0.451

Table 2: All measures results 50% removal

Measurements	Size change	Density change	Position change	Distribution effect	Dimensions effect
Average squared pairwise distance (dtt::dtt)	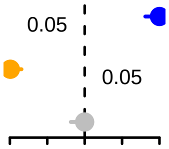	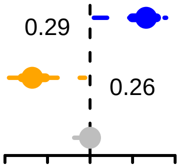	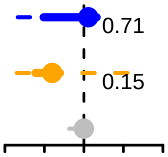	F = 1.23 ; p = 0.297	F = 0.615 ; p = 0.766

Measurements	Size change	Density change	Position change	Distribution effect	Dimensions effect
Average pairwise manhattan distance (dtt::dtt)	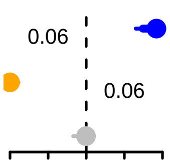	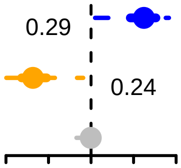	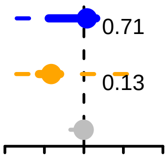	F = 1.717 ; p = 0.144	F = 1.105 ; p = 0.357
Median pairwise distance	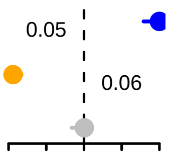	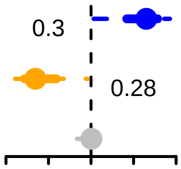	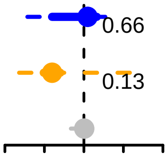	F = 1.054 ; p = 0.378	F = 0.548 ; p = 0.821
Median pairwise manhattan distance	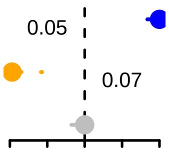	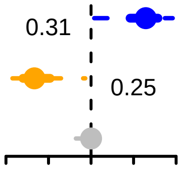	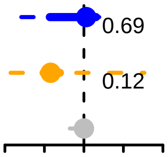	F = 1.132 ; p = 0.34	F = 0.984 ; p = 0.446
Procrustes variances (geomorph::morpho.disparity)	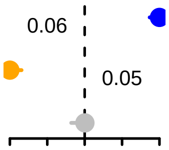	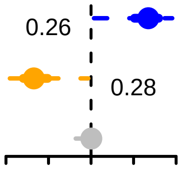	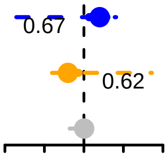	F = 0.016 ; p = 0.999	F = 0.021 ; p = 1
Median distance from centroid	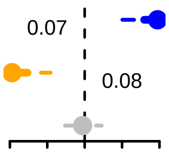	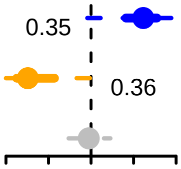	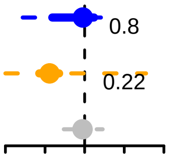	F = 2.251 ; p = 0.062.	F = 1.033 ; p = 0.409

Measurements	Size change	Density change	Position change	Distribution effect	Dimensions effect
Median manhattan distance from centroid	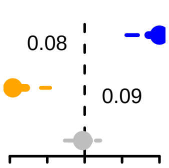	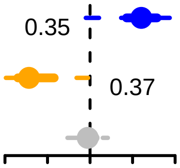	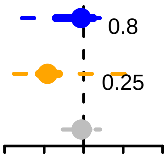	F = 1.108 ; p = 0.352	F = 0.631 ; p = 0.752
Sum of variances	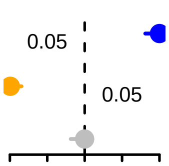	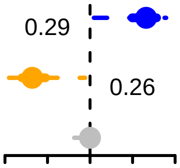	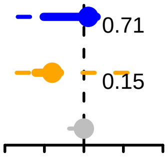	F = 1.23 ; p = 0.297	F = 0.615 ; p = 0.766
Sum of ranges	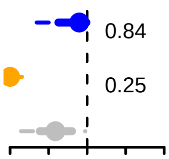	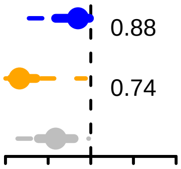	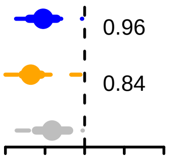	F = 6.12 ; p = <1e-3***	F = 41.228 ; p = <1e-3***
Sum of quantiles	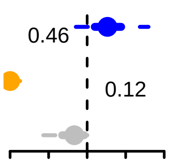	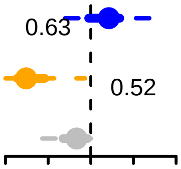	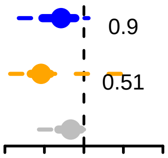	F = 4.332 ; p = 0.002**	F = 9.229 ; p = <1e-3***
Product of variances	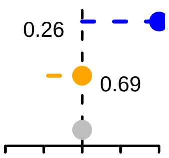	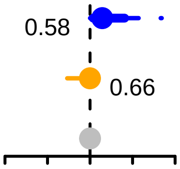	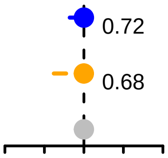	F = 11.612 ; p = <1e-3***	F = 3.476 ; p = 0.001**
Product of ranges	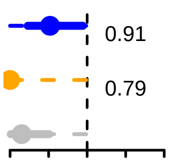	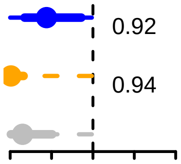	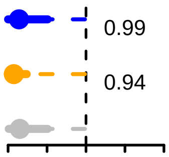	F = 1289.266 ; p = <1e-3***	F = 123.479 ; p = <1e-3***
Product of quantiles	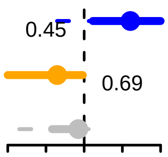	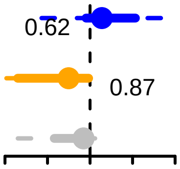	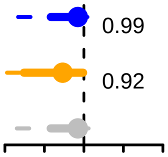	F = 72.676 ; p = <1e-3***	F = 83.723 ; p = <1e-3***

Measurements	Size change	Density change	Position change	Distribution effect	Dimensions effect
Ellipsoid volume	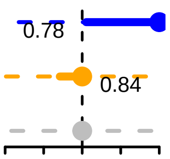	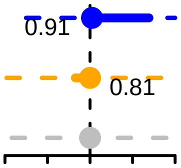	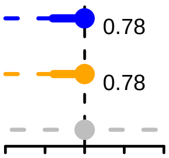	F = 10.662 ; p = <1e-3***	F = 13.492 ; p = <1e-3***
nBall volume	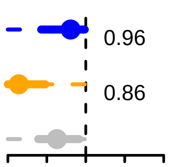	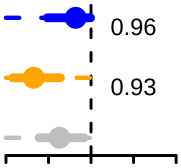	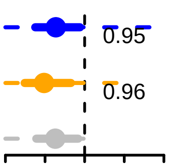	F = 8.106 ; p = <1e-3***	F = 49.126 ; p = <1e-3***
Minimum spanning tree average length	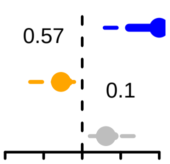	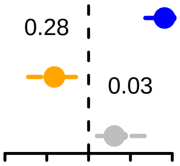	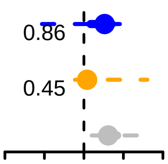	F = 1.167 ; p = 0.324	F = 0.99 ; p = 0.442
Average minimum neigh-bours distance	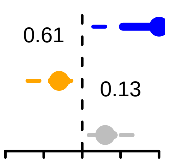	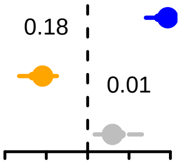	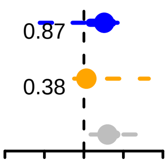	F = 1.54 ; p = 0.189	F = 1.013 ; p = 0.424
Average minimum neigh-bours manhat-tan distance	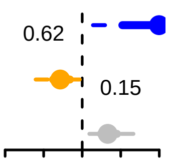	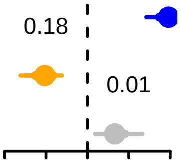	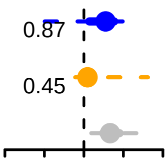	F = 1.842 ; p = 0.119	F = 1.672 ; p = 0.101
Function diversity	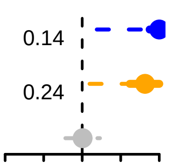	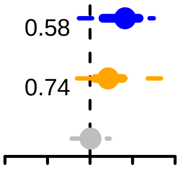	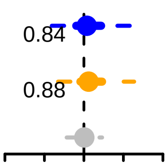	F = 1.274 ; p = 0.279	F = 10.59 ; p = <1e-3***

Measurements	Size change	Density change	Position change	Distribution effect	Dimensions effect
Functional evenness	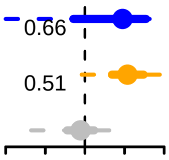	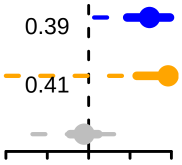	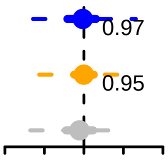	F = 8.117 ; p = <1e-3***	F = 26.957 ; p = <1e-3***
Functional dispersion	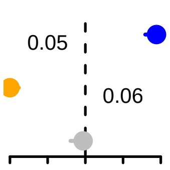	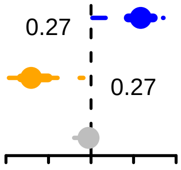	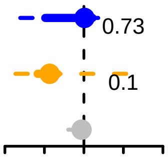	F = 0.817 ; p = 0.514	F = 0.395 ; p = 0.924
Average displacement	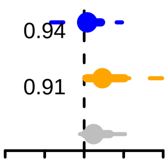	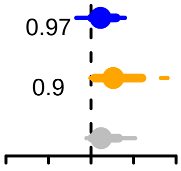	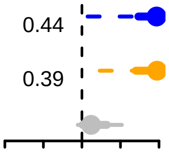	F = 9.066 ; p = <1e-3***	F = 29.094 ; p = <1e-3***
Average manhattan displacement	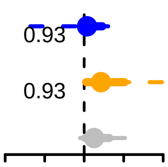	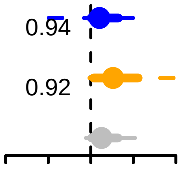	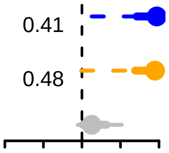	F = 12.223 ; p = <1e-3***	F = 27.991 ; p = <1e-3***
Median distance from centre	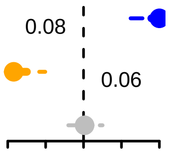	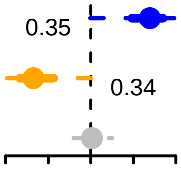	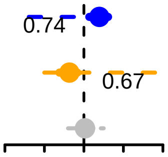	F = 0.582 ; p = 0.676	F = 0.121 ; p = 0.998
Median manhattan distance from centre	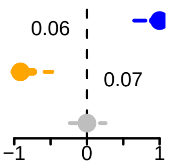	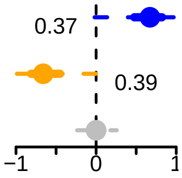	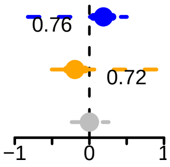	F = 0.343 ; p = 0.849	F = 0.15 ; p = 0.997

Table 3: All measures results 80% removal

Measurements	Size change	Density change	Position change	Distribution effect	Dimensions effect
Average squared pairwise distance (dtt::dtt)	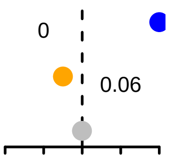	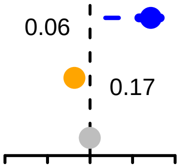	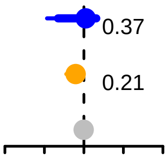	F = 1.036 ; p = 0.388	F = 1.688 ; p = 0.097.
Average pairwise manhattan distance (dtt::dtt)	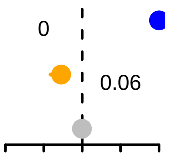	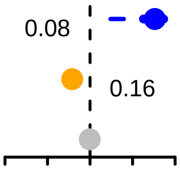	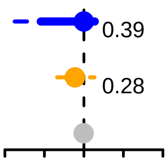	F = 1.76 ; p = 0.135	F = 3.338 ; p = 0.001**
Median pairwise distance	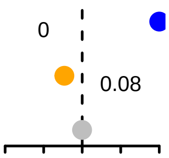	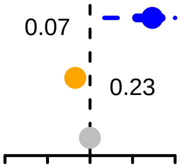	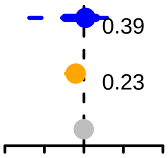	F = 1.195 ; p = 0.312	F = 2.16 ; p = 0.028*
Median pairwise manhattan distance	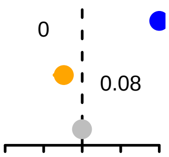	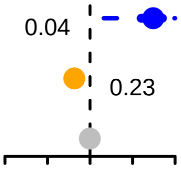	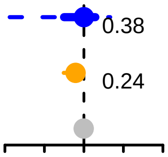	F = 1.027 ; p = 0.392	F = 3.207 ; p = 0.001**
Procrustes variances (geomorph::morpho.disparity)	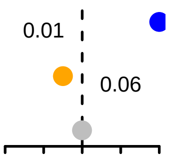	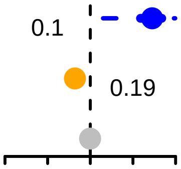	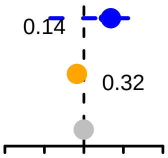	F = 0.444 ; p = 0.776	F = 0.217 ; p = 0.988

Measurements	Size change	Density change	Position change	Distribution effect	Dimensions effect
Median distance from centroid	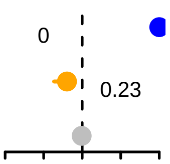	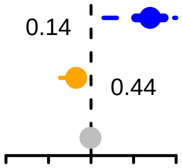	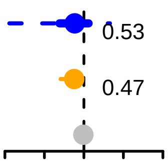	F = 0.967 ; p = 0.425	F = 3.473 ; p = 0.001**
Median manhattan distance from centroid	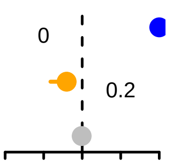	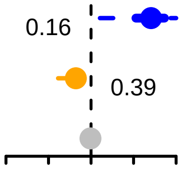	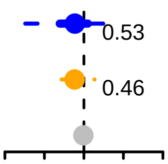	F = 0.579 ; p = 0.678	F = 2.653 ; p = 0.007**
Sum of variances	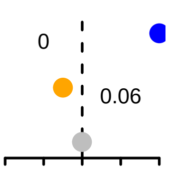	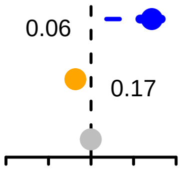	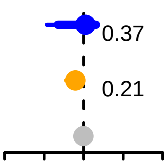	F = 1.036 ; p = 0.388	F = 1.688 ; p = 0.097.
Sum of ranges	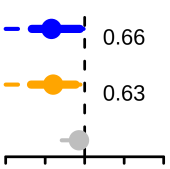	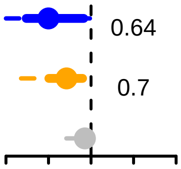	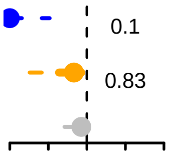	F = 3.066 ; p = 0.016*	F = 5.962 ; p = <1e-3***
Sum of quantiles	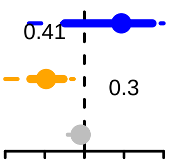	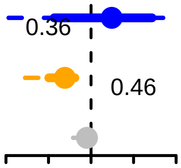	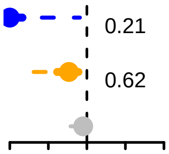	F = 10.889 ; p = <1e-3***	F = 14.988 ; p = <1e-3***
Product of variances	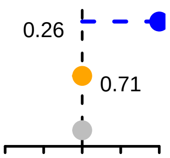	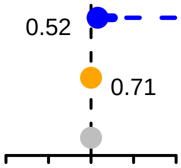	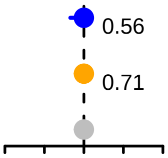	F = 9.465 ; p = <1e-3***	F = 1.115 ; p = 0.35
Product of ranges	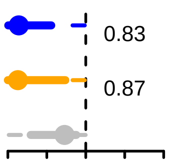	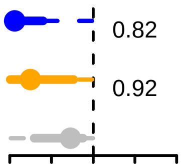	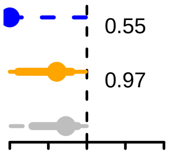	F = 562.45 ; p = <1e-3***	F = 92.288 ; p = <1e-3***

Measurements	Size change	Density change	Position change	Distribution effect	Dimensions effect
Product of quantiles	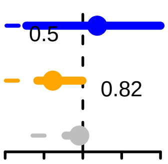	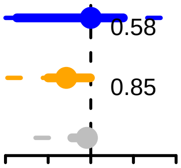	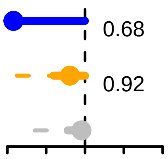	F = 102.197 ; p = <1e-3***	F = 129.722 ; p = <1e-3***
Ellipsoid volume	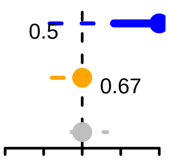	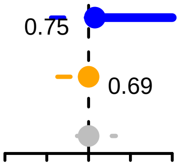	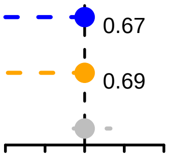	F = 1.479 ; p = 0.207	F = 5.213 ; p = <1e-3***
nBall volume	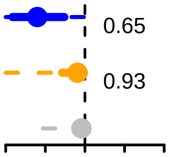	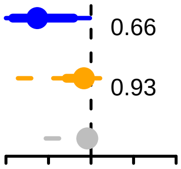	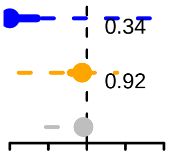	F = 2.839 ; p = 0.024*	F = 33.623 ; p = <1e-3***
Minimum spanning tree average length	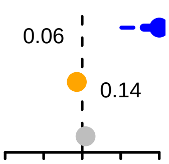	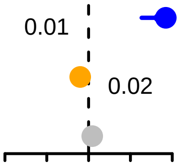	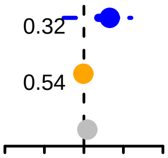	F = 1.123 ; p = 0.345	F = 0.113 ; p = 0.999
Average minimum neigh-bours distance	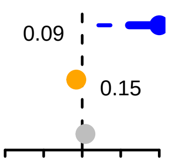	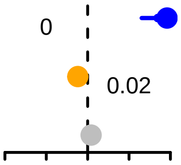	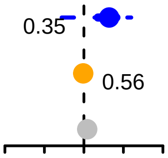	F = 1.637 ; p = 0.163	F = 0.751 ; p = 0.646
Average minimum neigh-bours manhat-tan distance	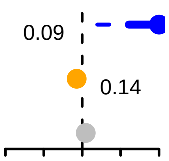	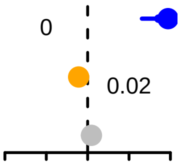	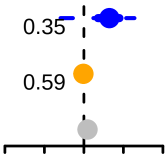	F = 1.816 ; p = 0.124	F = 1.004 ; p = 0.431

Measurements	Size change	Density change	Position change	Distribution effect	Dimensions effect
Function diversity	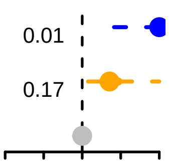	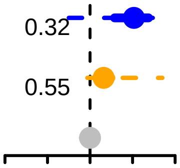	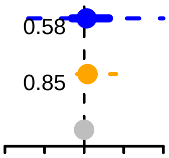	F = 7.744 ; p = <1e-3***	F = 6.619 ; p = <1e-3***
Functional evenness	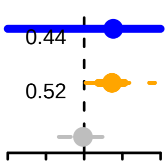	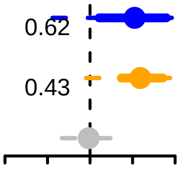	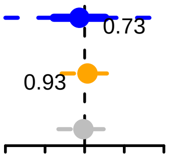	F = 22.732 ; p = <1e-3***	F = 26.826 ; p = <1e-3***
Functional dispersion	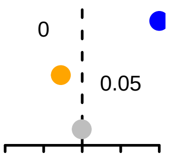	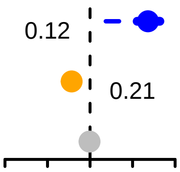	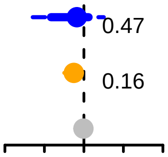	F = 0.975 ; p = 0.42	F = 1.788 ; p = 0.075.
Average displacement	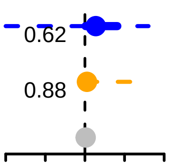	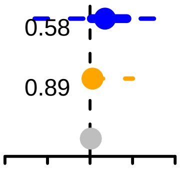	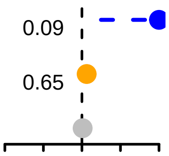	F = 8.548 ; p = <1e-3***	F = 6.475 ; p = <1e-3***
Average manhattan displacement	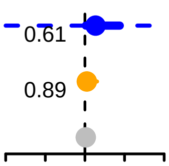	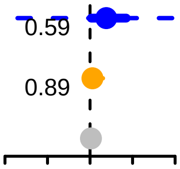	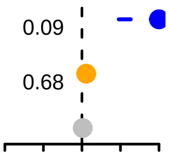	F = 16.025 ; p = <1e-3***	F = 6.537 ; p = <1e-3***
Median distance from centre	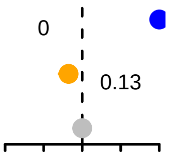	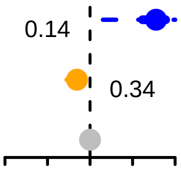	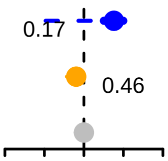	F = 0.421 ; p = 0.793	F = 0.424 ; p = 0.907

Measurements	Size change	Density change	Position change	Distribution effect	Dimensions effect
Median manhattan distance from centre	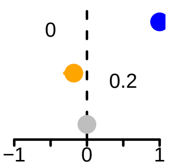	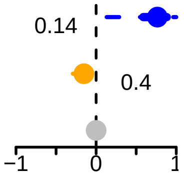	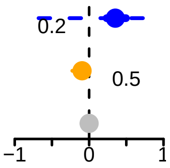	F = 0.83 ; p = 0.506	F = 0.412 ; p = 0.914

Empirical results (all measurements)

Measurement	Beck and Lee 2014	Wright 2017	Marcy et al. 2016	Hopkins et al. 2016	Jones et al. 2015	Healy et al. 2019
Average squared pairwise distance (dtt::dtt)	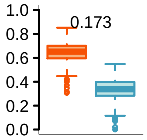	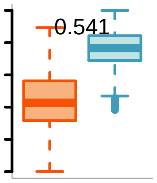	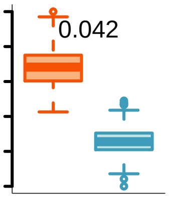	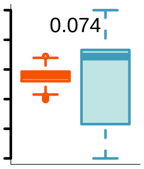	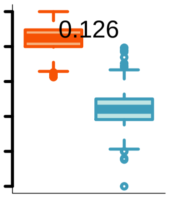	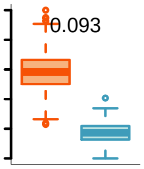
Average pairwise manhattan distance (dtt::dtt)	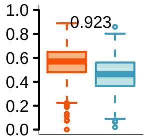	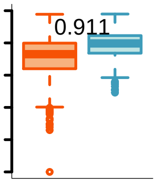	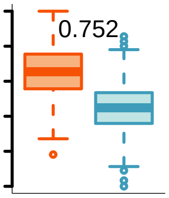	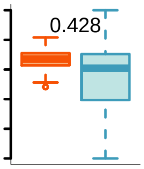	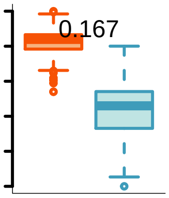	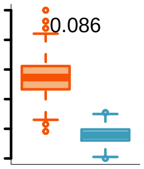
Median pairwise distance	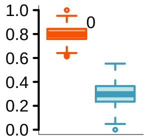	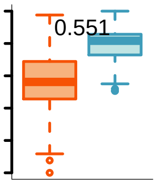	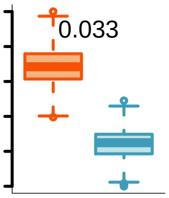	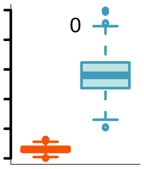	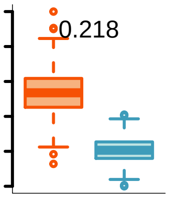	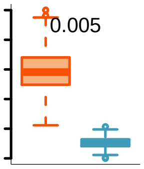

Measurement	Beck and Lee 2014	Wright 2017	Marcy et al. 2016	Hopkins et al. 2016	Jones et al. 2015	Healy et al. 2019
Median pairwise manhattan distance	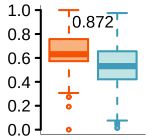	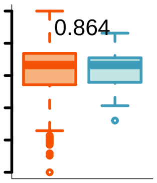	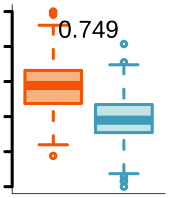	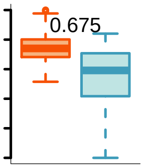	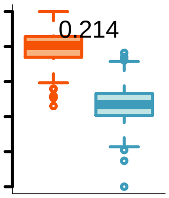	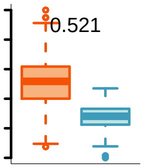
Procrustes variances (geomorph::morpho.disparity)	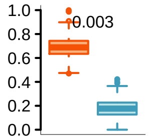	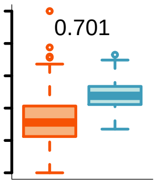	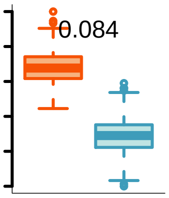	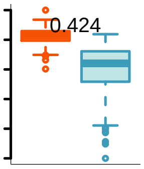	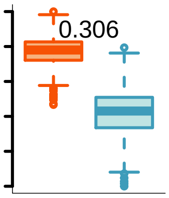	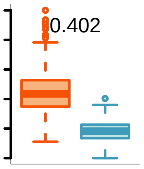
Median distance from centroid	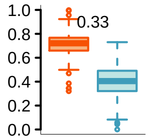	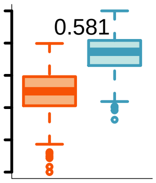	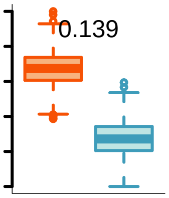	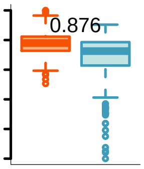	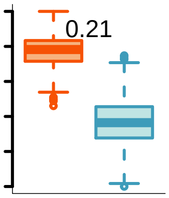	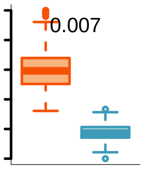
Median manhattan distance from centroid	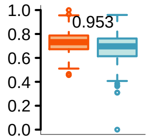	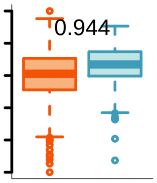	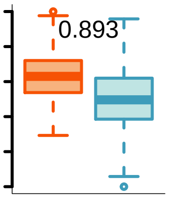	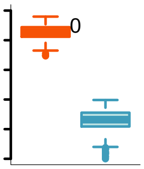	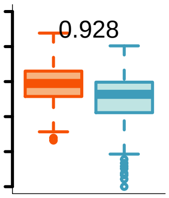	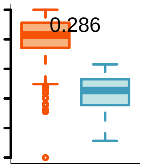
Sum of variances	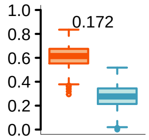	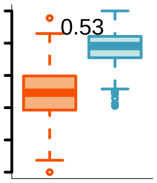	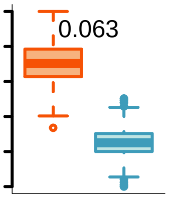	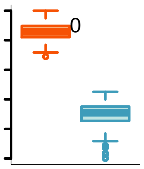	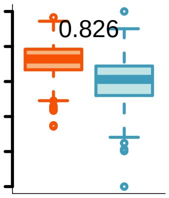	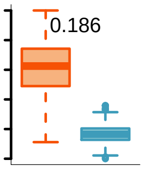

Measurement	Beck and Lee 2014	Wright 2017	Marcy et al. 2016	Hopkins et al. 2016	Jones et al. 2015	Healy et al. 2019
Sum of ranges	0.975	0.179	0.847	0	0	0.141
Sum of quantiles	0.968	0.157	0.558	0	0	0.307
Product of variances	0	0	NA	0	0	0.215
Product of ranges	0.926	0	NA	0	0	0.232
Product of quantiles	0.885	0	0	0	0	0.142
Ellipsoid volume	0	0	0.917	0.857	0.791	0.003

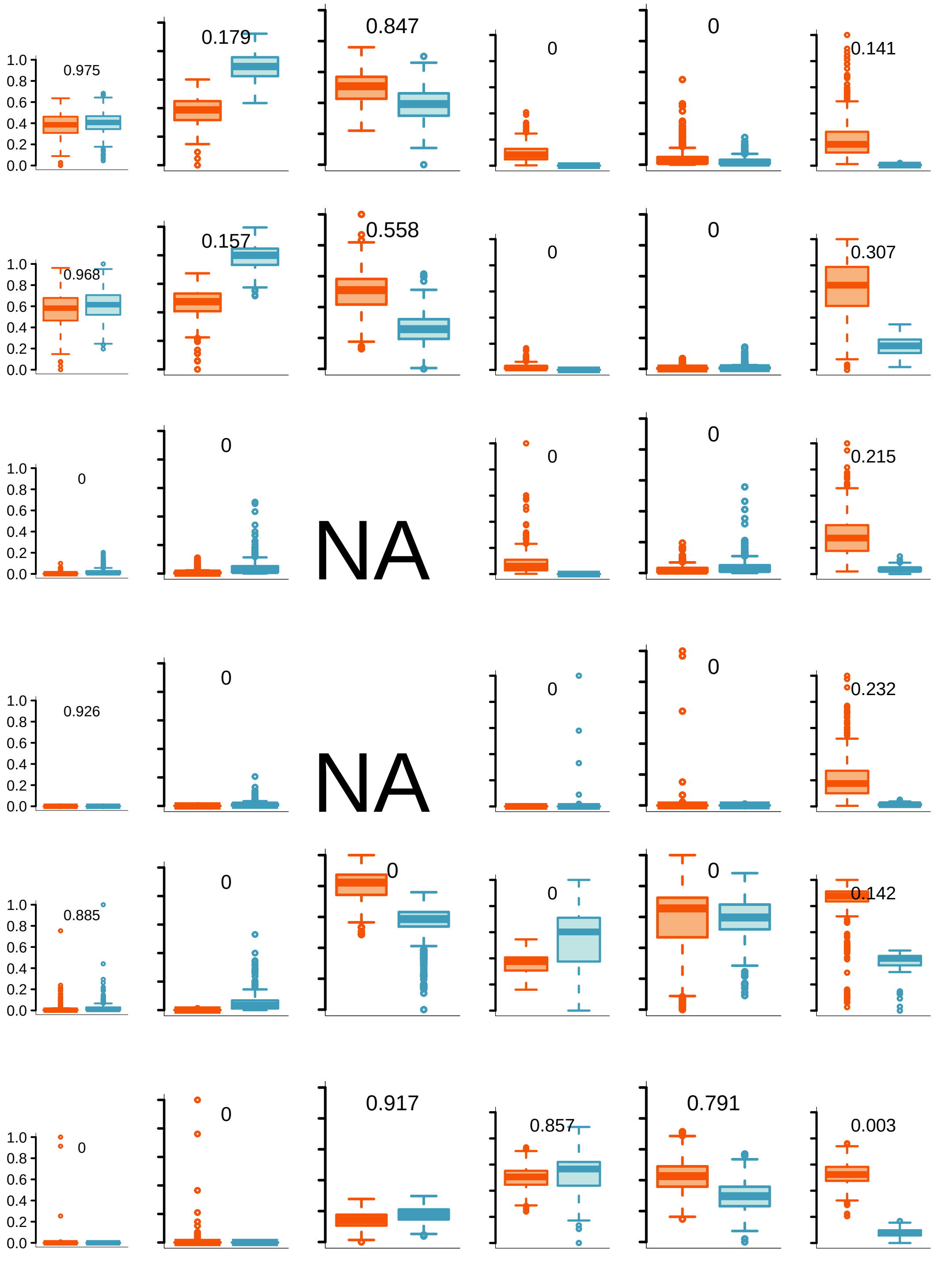

Measurement	Beck and Lee 2014	Wright 2017	Marcy et al. 2016	Hopkins et al. 2016	Jones et al. 2015	Healy et al. 2019
nBall volume	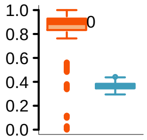	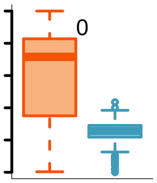	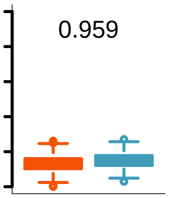	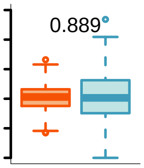	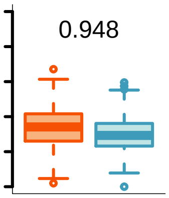	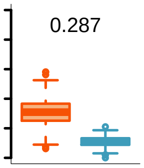
Minimum spanning tree average length	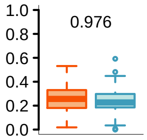	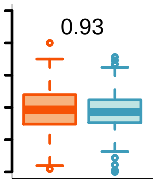	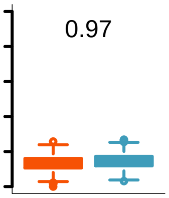	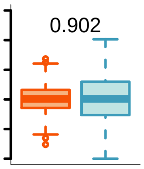	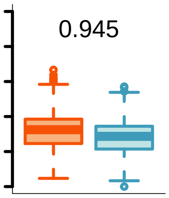	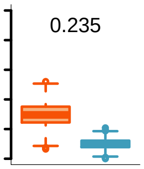
Average minimum neighbours distance	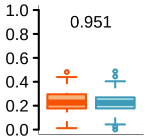	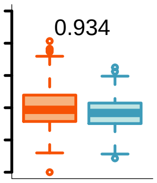	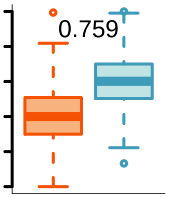	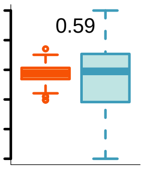	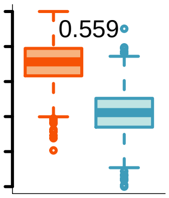	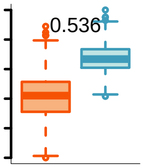
Average minimum neighbours manhattan distance	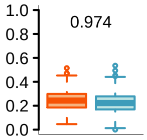	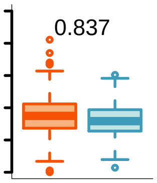	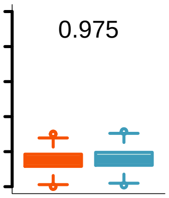	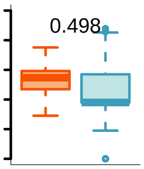	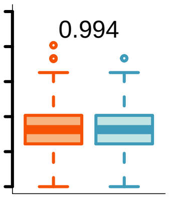	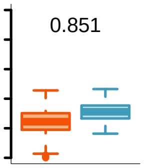
Function diversity	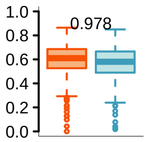	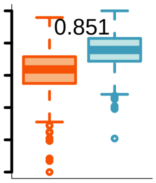	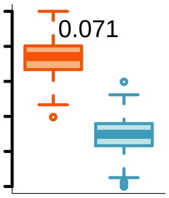	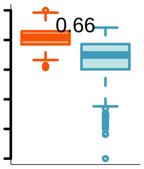	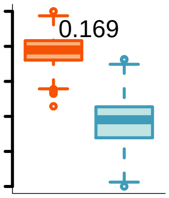	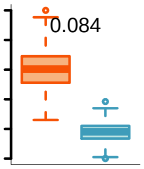

Measurement	Beck and Lee 2014	Wright 2017	Marcy et al. 2016	Hopkins et al. 2016	Jones et al. 2015	Healy et al. 2019
Functional evenness	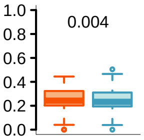	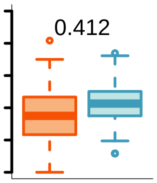	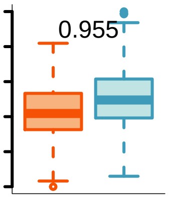	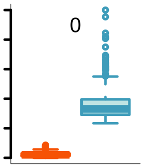	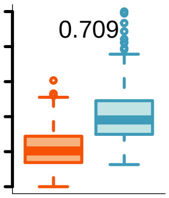	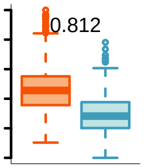
Functional dispersion	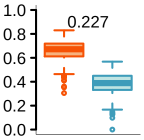	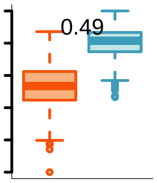	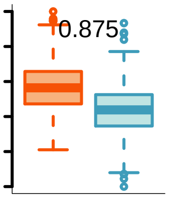	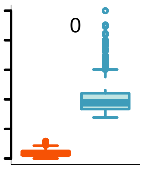	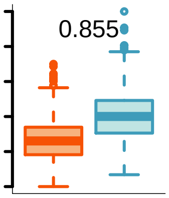	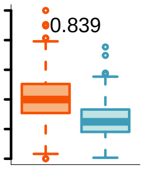
Average displacement	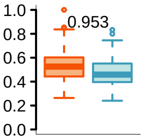	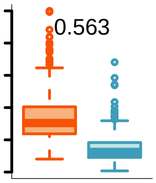	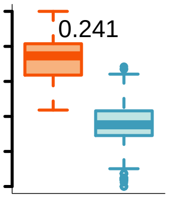	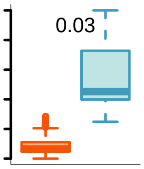	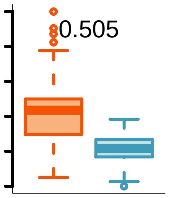	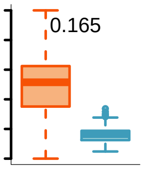
Average manhattan displacement	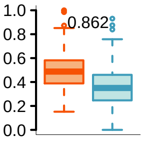	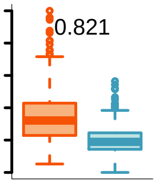	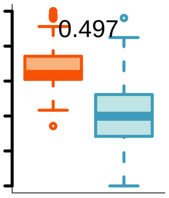	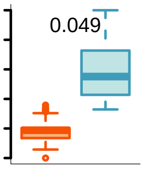	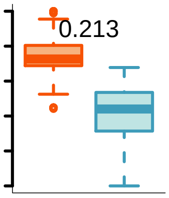	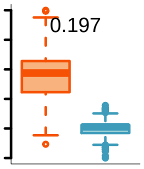
Median distance from centre	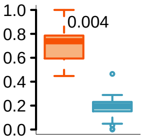	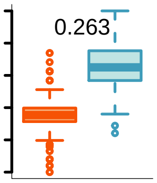	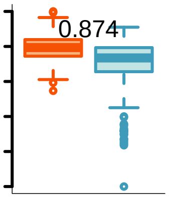	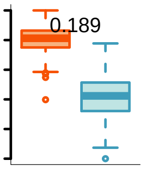	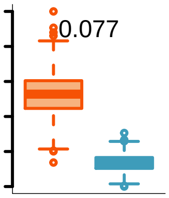	#r plot.id(149)
Median manhattan distance from centre	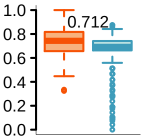	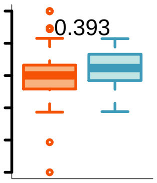	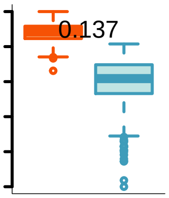	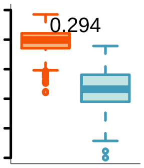	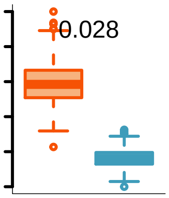	#r plot.id(150)

Measurement	Beck and Lee 2014	Wright 2017	Marcy et al. 2016	Hopkins et al. 2016	Jones et al. 2015	Healy et al. 2019
